# Implementing a competency-based acupuncture training program in Korean Medicine education

**DOI:** 10.1371/journal.pone.0345289

**Published:** 2026-03-20

**Authors:** Eunbyul Cho, Yeonkyeong Nam, Jiseong Hong, Yejin Han, Jae-Hyo Kim

**Affiliations:** 1 Department of Diagnostics, College of Korean Medicine, Wonkwang University, Iksan, Republic of Korea; 2 Research Center of Traditional Korean Medicine, Wonkwang University, Iksan, Republic of Korea; 3 Department of Meridian & Acupoint, College of Korean Medicine, Wonkwang University, Iksan, Republic of Korea; 4 7Days Research & Development Center, Seoul, Republic of Korea; 5 Department of Medical Education & Humanities, College of Medicine, Yeungnam University, Daegu, Republic of Korea; Endeavour College of Natural Health, AUSTRALIA

## Abstract

**Background:**

Acupuncture has gained global recognition as complementary and integrative therapy, but standardized education and training methods have not kept pace with its increasing application. Studies on systematic and effective acupuncture training approaches remain limited. We implemented and evaluated a newly designed acupuncture training program using a rapid prototyping instructional systems design (RPISD) model.

**Methods:**

An acupuncture training program was developed using the RPISD model through needs analysis, prototype design, and development, and was then implemented with undergraduate Korean Medicine students (n = 88) in 2022/2023. Program effectiveness was evaluated through a mixed-methods design incorporating pre- and post-program assessments using a satisfaction survey, the Korean self-efficacy questionnaire measuring clinical communication skills (KSE-12), students’ practical examination score, and in-depth interviews. Quantitative and qualitative findings were integrated through a convergent mixed-methods approach to provide a comprehensive evaluation of educational effectiveness and to identify areas for improvement.

**Results:**

Satisfaction was high, with 97.8% of 45 respondents expressing positive reactions to the program. Thirty-five students completed the communication questionnaire, with the total KSE-12 score significantly increasing after the course (*p* = 0.028). Students especially valued the hands-on practice and peer role-play, which improved their competencies in point location and clean needle technique. Interview findings also revealed areas for improvement, including allocating more time for practice and adopting a more gradual increase in learning content.

**Conclusion:**

The acupuncture training program improved students’ clinical competencies including clean needle technique, point location finding, and communication skills. This systematic approach to acupuncture education offers a replicable model for integrating traditional practices with evidence-based instructional design. The findings support broader implementation in acupuncture education programs and warrant investigation of long-term skill retention and cross-cultural applicability to advance global acupuncture training standards.

## Introduction

Acupuncture involving the insertion and manipulation of fine needles into specific points on the body has gained popularity worldwide as an integrative and complementary treatment in medical practice [1]. Systematic reviews have revealed various therapeutic areas of acupuncture therapy, including neurological disorders, connective tissue disorders, mental health, gastrointestinal disorders, and cancer [2]. Acupuncture is practiced not only in East Asian countries including China, Korea, and Japan, but also by physicians and professional acupuncturists in Europe according to regulations and insurance systems for acupuncture [3]. In the US, acupuncture by licensed acupuncturists and physicians for indications such as pain and allergies is covered by insurance in multiple states [4].

As acupuncture becomes increasingly institutionalized across global healthcare systems, growing attention has been directed toward how acupuncture is taught and learned. Recent literature has highlighted several structural limitations in existing acupuncture education, including fragmented curricula, lacking standardized competency frameworks, and limited opportunities for contextual and interactive learning [5,6]. Such challenges indicate the need for more systematic, learner-centered, and clinically grounded training models that can cultivate essential practical competencies.

In East Asia, acupuncture has traditionally been embedded within formal education systems of traditional medicine. [7]. Among these, South Korea offers a nationally accredited six-year Korean Medicine (KM) curriculum in which acupuncture is taught as a core component during one or two intensive years. Licensed KM doctors who complete this curriculum are authorized to practice acupuncture with national insurance coverage [8], and acupuncture remains one of the most commonly used treatment modalities in KM clinical practice [9]. However, despite its centrality in KM education, well-developed instructional methodologies for systematically cultivating clinical acupuncture competencies remain limited.

Within this curriculum, meridian and acupuncture point practice—where students gain foundational experience in palpation, point location, and basic needling—plays a crucial role. However, conventional practice sessions largely rely on instructor-centered demonstrations and basic point-location exercises, resulting in insufficient structured guidance, limited iterative practice, and inconsistent feedback mechanisms. In our prior analysis within the initial RPISD stages, we identified recurring challenges such as difficulty in locating acupoints, low confidence during needling, inadequate adherence to clean needle technique, and insufficient communication and coordination among students during practice [10,11].

To address these limitations and enhance students’ acupuncture proficiency, we adopted an instructional system design model from the field of educational technology and created a novel training program. The rapid prototyping instructional systems design (RPISD) model allows iterative and simultaneous development of educational materials through stakeholder feedback, in contrast to traditional linear models. Its flexible and feedback-driven structure makes it suitable for developing competency-based clinical training programs [12]. The present study aims to evaluate the implementation and effectiveness of a novel acupuncture training program designed using the RPISD model in KM education context. The findings will provide evidence-based teaching and learning strategies that can be applied in various acupuncture education settings.

## Materials and methods

**Fig 1:**
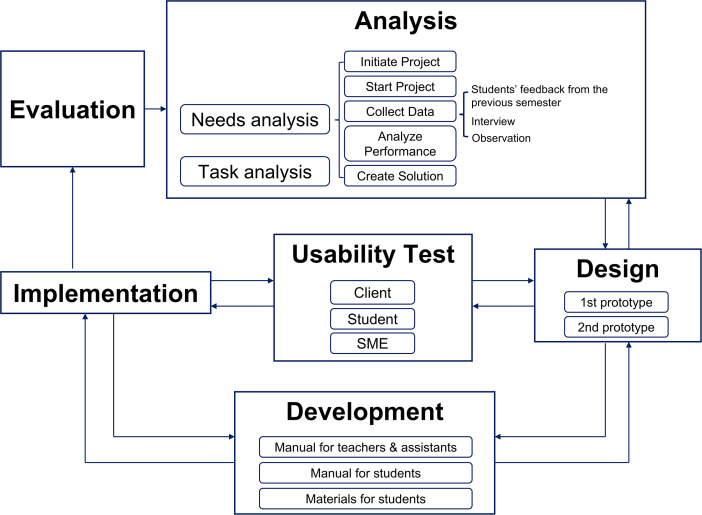
Rapid Prototyping Instructional System Design (RPISD) model applied to program development.

This study applied the RPISD model to design and evaluate an acupuncture training program. The model includes five overlapping phases: analysis, design and usability testing of early prototypes, development, implementation, and evaluation [12]. Rather than proceeding sequentially, these phases were conducted iteratively, with continuous refinement based on feedback from students, faculty, and subject matter experts. The early phases of analysis, design, and prototype development were previously described in detail [10,11]. This study focuses on the subsequent implementation and evaluation of the training program. Fig 1 outlines how each phase was applied in this study.

### Design and development of the program

In the analysis phase, we conducted needs and task analyses to identify gaps between the current status (as-is) and ideal educational situation (to-be) regarding students’ knowledge, skills, and attitudes. Data was collected through student feedback, interviews, and direct classroom observations. Identified key performance gaps included inadequate infection control practices, low confidence in point location and needling techniques, and poor communication between the physician and patient role students. The resulting educational solutions incorporated individual CNT procedure performance, structured role-play guidelines for physician-patient-observer interactions, and peer-based assessment strategies [13]. Additionally, through task analysis, five learning objectives were established: 1) measure vital signs of the patient, 2) accurately locate acupuncture points on the 14 meridians, 3) perform acupuncture procedures hygienically and safely, 4) appropriately utilize medical tools and devices required for the procedure, and 5) accurately record the procedural details.

We designed the first prototype lesson plan using peer role-play, George’s five methods for teaching clinical skills [14], and the Objective Structured Clinical Examination (OSCE) as instructional strategies. The initial prototype was developed for the topic of ‘auricular acupuncture’, and usability testing was conducted through interviews with clients, students, and subject matter experts. After the usability testing, the revised lesson plan included more specific roles for doctors and observers in each class session [10].

Incorporating client requirements, the 1-year acupuncture training program was organized into four major topics: Clean needle technique and needling techniques, Measuring vital signs, Locating acupuncture points and needling, and Acupuncture techniques. Subsequently, we developed teaching and learning manuals including lesson plans for each of the four topics, class guidelines, and equipment utilization protocols [15]. Clean needle technique and needling techniques and measuring vital signs are covered in the early part of each semester, with locating acupuncture points and needling on the 14 meridians comprising the majority of the curriculum. Additionally, various acupuncture, moxibustion, and cupping techniques are addressed (S1 File).

### Implementation of the acupuncture training

The program was implemented as a regular one-year course at the College of Korean Medicine, Wonkwang University, South Korea. The course was conducted over two semesters: the second semester of 2022 (45 students) and the first semester of 2023 (43 students), with different student cohorts participating in each semester. The two cohorts did not differ significantly in baseline characteristics, including age (p = .767), gender (p = .546), and overall GPA in the preceding semester (p = .158).

Manuals were provided via Google Classroom, and paper versions were provided in class. Students watched a video lecture with hands-on demonstration before class and took notes on the tasks they would perform during class. Roles were assigned in each class: one observer, at least one patient, and the rest physicians.

**Fig 2:**
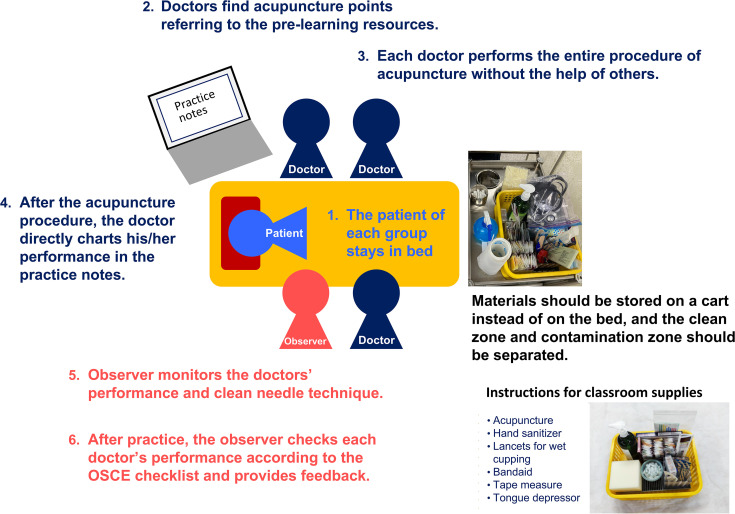
Group practice structure showing peer role-play and OSCE-based acupuncture training. Students are divided into groups with assigned roles: observers (who evaluate performance using checklists), patients (who provide feedback on communication and comfort), and physicians (indicated as ‘doctor’ in the figure, who practice acupuncture point location and needling techniques). This role rotation system allows all students to experience different perspectives while developing clinical competencies in a safe learning environment.

**Fig 3:**
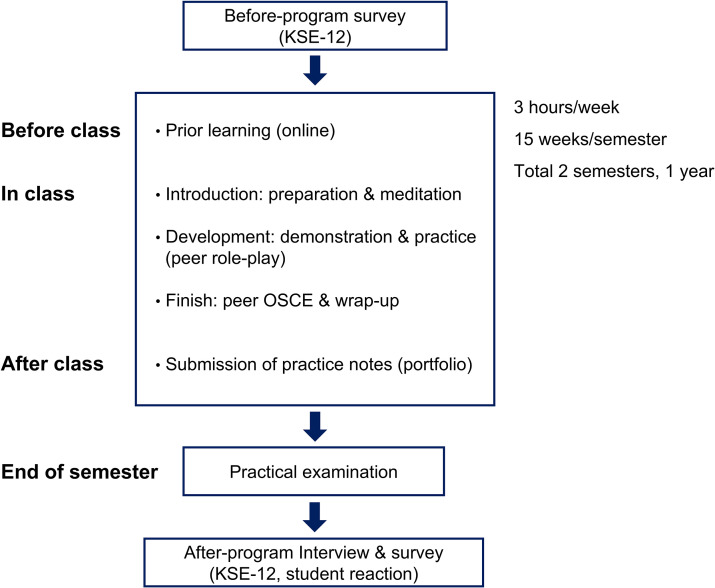
Overall structure of the training program and research process. The one-year acupuncture training program (3 hours/week, 15 weeks/semester, 2 semesters) follows a systematic approach with pre-class online learning, structured in-class activities, and post-class portfolio submission. Each class session includes three phases: introduction (preparation and meditation), development (teacher demonstration followed by peer role-play practice), and finish (peer OSCE assessment and wrap-up). Communication skills were assessed using the Korean Self-Efficacy questionnaire before and after the program, with practical examinations conducted at semester end. Student reactions and feedback were collected through surveys and interviews to evaluate program effectiveness.

Each class session was structured into three phases: introduction, development, and wrap-up. In the “introduction” phase, students did preparations: washing their hands, putting on bed sheets, placing the group supplies near the bed, and obtaining informed consent from the patient. The teacher presented the class’s learning objectives. In the “development” phase, the teacher demonstrated the procedure for finding acupuncture points and performing acupuncture, following which students practiced finding acupuncture points and performing acupuncture in groups (Fig 2). Students then conducted peer OSCE to check their performance and receive feedback from students acting as observers, teachers, and teaching assistants. In the “wrap-up” phase, students cleaned up, organized the supplies used, and completed a post-procedure checklist. After class, each group was given practice notes that included the results of the observer’s hygiene and safety checks, how the physicians found and needled each acupuncture point, and the patient’s experience (Fig 3).

### Evaluation of the acupuncture training program

This study was approved by the Institutional Review Board of Wonkwang University (WKIRB-202208-SB-069). All participants were recruited via student representative announcements and provided written informed consent prior to participation. The recruitment periods for survey participants were August 30, 2022 (the second semester of 2022, conducted over a single day), and March 7, 2023 (the first semester of 2023, conducted over a single day), while the recruitment period for interview participants was from July 1 to July 12, 2023. A convergent mixed-methods research design was employed to evaluate whether the training program achieved its intended learning objectives. We used Kirkpatrick’s reaction evaluation [16] to judge the program’s effectiveness by conducting surveys and interviews to acquire both overall and in-depth responses. Written informed consent was obtained from students who participated in surveys and interviews.

### Data collection and analysis

To collect students’ reactions to the program, we used survey items from previous literature, adapted to suit the present study [13,17,18]. The survey included 27 multiple-choice (program’s usefulness, adequacy of learning materials, etc.) rated on a 5-point Likert scale (strongly agree to strongly disagree) and five open questions (S2 File). Surveys were done at the end of each semester with participants. Responses with missing items were excluded for reliability. The internal consistency of the instrument was high (Cronbach’s α = .923).

Communication skills were assessed using the validated Korean version of the self-efficacy questionnaire (KSE-12) [19], administered in the first and last weeks of each semester for pre-post comparison. Axboe et al.’s SE-12 questionnaire [20] measures healthcare providers’ communication skills during patient interactions. The 12 items in KSE-12 are rated on a scale of 1–10 (1 = very uncertain, 10 = very certain). The internal consistency of the KSE-12 in this study was high (Cronbach’s α = .934). A paired t-test or Wilcoxon signed-rank test was used to compare KSE-12 scores before and after class.

To determine our program’s effectiveness, we examined students’ scores of practical examination pre- and post-implementations. Although the test was conducted at the same grade level using the same checklist, the results were only used as reference data due to the different target students. All quantitative data were analyzed and presented using R statistical software (version 4.2.2, R Core Team, 2024) and GraphPad Prism (version 10.2.2; GraphPad Software, Boston, MA, USA).

For qualitative interviews, purposeful sampling initially selected participants representing diverse academic performance levels and program engagement, followed by snowball sampling to recruit additional participants with varied backgrounds. This combined approach was employed to ensure varied perspectives on the training experience. The main interview questions were set as “What are the strengths of this course?” and “What are the weaknesses and improvements of this course?” In-depth, one-on-one interviews were conducted online, each lasting approximately one hour. All interviews were audio-recorded and transcribed verbatim with participants’ informed consent.

The interview data were analyzed according to Braun and Clarke’s six steps of thematic analysis: Familiarization of data, generation of codes, combining codes into themes, reviewing themes, determining significant themes, and reporting of findings [21]. In the event of disagreement among researchers when generating codes or themes for the interview data, the codes were revised through discussion and consensus.

Finally, qualitative and quantitative results were integrated and compared in accordance with the objectives of this program.

## Results

### Satisfaction with the program

Overall satisfaction with the program reached 97.8%, with most items receiving positive responses above 80%, including usefulness of learning materials, appropriateness of learning activities, and communication in class. However, positive responses were fewer than 50% regarding the adequacy of time allocated to each training stage and pre-class videos (Table 1). Open-ended responses on reasons for satisfaction included the class being “well organized,” “writing practice notes,” and it would be “helpful in clinical practice.” Regarding the effectiveness of the teaching method, students stated they were “able to practice efficiently through flipped learning” and they favored “the practice based on the professor’s demonstration.” Almost 98% of respondents agreed that the purpose and content of the acupuncture training were in line with the objectives. For improvement, “more time for students to practice in class” was recorded.

**Table 1:** Survey results on satisfaction and instructional components.

No.	Survey questions	Strongly disagree	Disagree	Neutral	Agree	Strongly agree
1	Was the class interesting?	0 (0)	0 (0)	1 (2.2)	25 (55.6)	19 (42.2)
2	Was the level of training appropriate?	0 (0)	0 (0)	2 (4.4)	24 (53.3)	19 (42.2)
3	Was the learning material directly related to the learning objectives?	0 (0)	0 (0)	1 (2.2)	15 (33.3)	29 (64.4)
4	Was the learning material easy to understand?	0 (0)	0 (0)	7 (15.6)	24 (53.3)	14 (31.1)
5	Was the learning material useful?	0 (0)	0 (0)	4 (8.9)	18 (40.0)	23 (51.1)
6	Was the content of learning material clear?	0 (0)	0 (0)	3 (6.7)	16 (35.6)	26 (57.8)
7	Was the practice opportunity sufficient?	0 (0)	1 (2.2)	7 (15.6)	19 (42.2)	18 (40.0)
8	Do you think the peer OSCE activity in the class is appropriate?	0 (0)	0 (0)	1 (2.2)	20 (44.4)	24 (53.3)
9	How satisfied are you with the training program overall?	0 (0)	0	1 (2.2)	20 (44.4)	24 (53.3)
10	Was the training method effective?	0 (0)	0 (0)	5 (11.1)	25 (55.6)	15 (33.3)
11	Was the purpose and content of the training aligned in your opinion?	0 (0)	1 (2.2)	0 (0)	18 (40)	26 (57.8)
12	Was the amount of time given for this class during this semester adequate?	0 (0)	1 (2.2)	6 (13.3)	25 (55.6)	13 (28.9)
13	Was the time allotted for preparation, presentation of learning objectives, and meditation (introduction session, 20 minutes) sufficient?	0 (0)	5 (11.1)	21 (46.7)	10 (22.2)	9 (20)
14	Was the time allotted for the teacher’s presentation (demonstration) (development session, 30 minutes) sufficient?	4 (8.9)	6 (13.3)	19 (42.2)	7 (15.6)	9 (20)
15	Was the time allotted for the group exercises and wrap-up (development and wrap-up session, 100 minutes) sufficient?	1 (2.2)	8 (17.8)	17 (37.8)	10 (22.2)	9 (20)
16	Was the average class time for online prior learning appropriate?	1 (2.2)	11 (24.4)	13 (28.9)	13 (28.9)	7 (15.6)
17	Are learning videos attractive to promote learning?	0 (0)	7 (15.6)	14 (31.1)	16 (35.6)	8 (17.8)
18	Did you watch the learning videos for review?	2 (4.4)	6 (13.3)	7 (15.6)	17 (37.8)	13 (28.9)
19	Does prior online learning increase your understanding of classes?	0 (0)	0 (0)	4 (8.9)	25 (55.6)	16 (35.6)
20	Was it convenient to access the online learning management system?	0 (0)	0 (0)	3 (6.7)	18 (40)	24 (35.6)
21	Were you able to study freely at your desired time and place?	0 (0)	0 (0)	0 (0)	18 (40)	27 (60)
22	Did learning activities make learning more interesting?	1 (2.2)	1 (2.2)	5 (11.1)	24 (53.3)	14 (31.1)
23	Were the learning activities appropriate to achieve the learning objectives?	0 (0)	0 (0)	3 (6.7)	26 (57.8)	16 (35.6)
24	Was communication between the instructor and the student communication good?	0 (0)	0 (0)	7 (15.6)	17 (37.8)	21 (46.7)
25	Was communication between the students good?	0 (0)	0 (0)	5 (11.1)	17 (37.8)	23 (51.1)
26	Was the learning activity appropriately assessed?	0 (0)	0 (0)	1 (2.2)	24 (53.3)	20 (44.4)
27	Was the content alignment between pre-learning and classroom learning appropriate?	0 (0)	1 (2.2)	4 (8.9)	20 (44.4)	20 (44.4)

Responses are presented in frequency (%).

### Communication self-efficacy

The average KSE-12 total score significantly increased from 82.43 ± 17.09 before the course to 88.89 ± 13.27 after the course (*p* = 0.028). The mean scores for each of the 12 items all increased after the course, but the statistically significant results were shown in items 5 (“encourage the patient to express thoughts and feelings”’) (*p* = 0.029), 6 (“structure the conversation with the patient”) (*p* = 0.015), and 10 (“check patient’s understanding of the information given”) (*p* = 0.044) (Table 2, S3 File).

**Table 2:** Pre-post scores on the Korean version of self-efficacy questionnaire for communication skills.

No.	Items of KSE-12	Before class	After class	*p*-value
1	How certain are you that you are able to successfully identify the issues the patient wishes to address during the conversation?	6.37 ± 2.06	6.77 ± 1.52	0.436
2	How certain are you that you are able to successfully make an agenda/plan for the conversation with the patient?	7.14 ± 1.85	7.77 ± 1.46	0.090
3	How certain are you that you are able to successfully urge the patient to expand on his or her problems/worries?	7.29 ± 2.02	7.57 ± 1.50	0.516
4	How certain are you that you are able to successfully listen attentively to the patient?	8.09 ± 1.70	8.40 ± 1.31	0.222
5	How certain are you that you are able to successfully encourage the patient to express thoughts and feelings?	7.17 ± 1.79	7.86 ± 1.44	0.029*
6	How certain are you that you are able to successfully structure the conversation with the patient?	6.71 ± 1.81	7.57 ± 1.48	0.015*
7	How certain are you that you are able to successfully demonstrate appropriate nonverbal behaviors (eye contact, facial expression, placement, posture, and voicing)?	7.34 ± 1.61	7.71 ± 1.58	0.254
8	How certain are you that you are able to successfully show empathy (acknowledge the patient’s views and feelings)?	7.09 ± 1.90	7.69 ± 1.55	0.109
9	How certain are you that you are able to successfully clarify what the patient knows in order to communicate the right amount of information?	6.03 ± 1.92	6.54 ± 1.70	0.166
10	How certain are you that you are able to successfully check patient’s understanding of the information given?	6.14 ± 1.93	6.91 ± 1.50	0.044*
11	How certain are you that you are able to successfully make a plan based on shared decisions between you and the patient?	6.46 ± 1.72	6.94 ± 1.61	0.170
12	How certain are you that you are able to successfully close the conversation by assuring, that the patient’s questions have been answered?	6.6 ± 2.08	7.14 ± 1.52	0.175
Total		82.43 ± 17.09	88.89 ± 13.27	0.026*

Data are presented as mean±standard deviation. KSE-12, the Korean version of Self-efficacy Questionnaire. *P*-values were calculated using the paired t-test or the Wilcoxon signed rank test. *p < 0.05.

### Changes in practical examination scores

The total score on the practical examination was higher after implementing the newly designed program, although different students took the test. Items that increased after the program included the process and accuracy of acupuncture point location, safety of the patient, and communication with the patient. Specifically, the total practical examination score increased from 48.47 ± 10.10 to 54.63 ± 8.28. The most substantial improvements were observed in the process of acupuncture point location (13.76 ± 3.59 to 16.09 ± 3.24, *p* < 0.001) and accuracy of acupuncture point location (13.45 ± 3.41 to 16.30 ± 3.01, *p* < 0.001). Notable enhancements were also seen in communication with the subject (3.13 ± 1.06 to 3.80 ± 0.48, *p* < 0.001) and safety of the subject (3.16 ± 0.98 to 3.75 ± 0.49, *p* < 0.001), indicating improved clinical competencies aligned with the program’s learning objectives (Table 3, S4 File).

**Table 3:** Pre-post changes in practical examination scores after implementation of the training program.

Score	Before class	After class	*p*-value
Process of acupuncture point location (0–20)	13.76 ± 3.59	16.09 ± 3.24	<0.001*
Accuracy of acupuncture point location (0–20)	13.45 ± 3.41	16.30 ± 3.01	<0.001*
Process of acupuncture and ability to use tools (0–4)	2.41 ± 1.48	2.14 ± 1.77	0.462
Accuracy of acupuncture procedure (0–4)	2.46 ± 1.59	2.14 ± 1.88	0.400
Appropriateness of patient positioning (0–4)	3.42 ± 0.80	3.60 ± 0.69	0.126
Safety of the subject (0–4)	3.16 ± 0.98	3.75 ± 0.49	<0.001*
Communication with the subject (0–4)	3.13 ± 1.06	3.80 ± 0.48	<0.001*
Hygiene management (0–4)	3.07 ± 1.17	3.51 ± 0.76	0.014*
Post-procedure cleanup (0–4)	3.62 ± 0.80	3.31 ± 0.89	0.014*
Total	48.47 ± 10.10	54.63 ± 8.28	<0.001*

P-values were obtained by the Wilcoxon rank sum test. *p < 0.05.

### Qualitative results

Thematic analysis of the interviews revealed four main themes related to the strengths of the course and four themes concerning weaknesses and suggested improvements (Table 4).

**Table 4:** Themes from student interviews.

Classification	Theme	Frequency*
Strengths of the course	Self-reflection through the role (physician, patient, observer)	4
	Improved competency in finding acupuncture points and acupuncture through practical exercises	3
	Systematic learning progress through learning manuals	3
	Improved habits of hygiene and safety	3
Limitations and solutions of the class	More time to practice acupuncture point finding and acupuncture	6
	Gradual increase in the amount of learning content per week	4
	More opportunities to observe instructor demonstrations up close	2
	Strategies to facilitate pre-class learning	2

* Number of participants.

**Strengths of the course.** First, students experienced self-reflection in various roles. Observers watched and recorded other students’ performance, enabling them to ascertain what others did well or not, which stimulated reflection on their own practices. Students who played the role of ‘patients’ realized the importance of communication, such as providing reassurance to help them relax during treatment.

*I thought it was really important to relax the patient when I am doing acupuncture as a physician, so I thought a lot about the importance of communicating with the patient, so I tried to do a lot more of that. (Student 3)*

Second, students’ ability to locate acupuncture points and perform acupuncture improved due to hands-on practice. Repeated practice in a safe, supervised environment appeared to enhance their clinical performance.

*Actually, I think the biggest benefit of the practice is that we’ve never held a needle before, so it’s good to be able to practice the most basic acupuncture skill, and (...) I think I was really nervous at first putting the needle on someone else’s body, but I think it’s good to be able to practice it over and over again and get a little more comfortable with it. (Student 2)*

Third, the learning was well structured by the course’s learning manual. Students said that, since this was their first experience with acupuncture practice, they might have been confused about the practical steps they should perform. The manual assisted them to optimize their performance because it was organized systematically.

*I felt very strongly that everything was programmed in a very structured way, so I think that was a huge benefit. I think that (the manual) allowed us to do more standardized and optimal practice in a way. (Student 6)*

Fourth, students established hygiene and safety habits. An objective of the course was “to be able to perform acupuncture procedures hygienically and safely.” Students had to check and confirm their compliance with hygiene and safety management (hand sanitizing, wearing lab coats, preparing and organizing supplies, etc.). The students reported that these habits were naturally formed through repeated hygiene and safety management activities, and that they felt confident with the Clean Needle Technique (CNT).

*Now I know what it takes to do a procedure on a patient in a more stable environment, I can do it if I follow the same steps as in the practice settings (...) It’s a great way to learn more about CNT. (Student 2).*

**Limitations and solutions of the class.** Four suggested weaknesses and improvements of the course have been received from students. First, more time was needed to practice acupuncture point finding and acupuncture. Sometimes, 20 acupuncture points had to be found in a single class, leaving insufficient time for all students to practice it. Reducing the time spent on other class activities and increasing time for acupuncture practice was identified for improvement.

*I think we were a little more pressed for time because we were discussing and trying to figure out where to put the needle. I think we’re running out of time. (Student 3)*

Second, it is necessary to gradually increase the volume of learning content per week. Students were at first unfamiliar with the practice, so activities such as CNT took a long time. Toward the end of the course, they have adapted to the relevant practices. Students consequently suggested reducing the amount of learning content at the beginning and increasing it toward the end. Thus, more time should be allocated for preparation and adaptation to the practice of acupuncture at the beginning of the course and the extent of learning content should be increased toward the end of the course.

*In the beginning it can be time consuming with the CNTs, but we get more comfortable with the CNTs and the phases of practice towards the end of the course, so I would like them to be a little bit flexible and elastic with the time, for example reducing the number of acupuncture points at the beginning of the course and increasing it a little bit towards the end of the course. (Student 6)*

Third, more opportunities for students to observe instructor demonstrations should be allowed. Further to instructors’ demonstration, students wanted to observe instructors’ demonstrations up close. For optimal practice, instructors and tutors should rotate among groups to demonstrate procedures and provide detailed feedback based on individuals or groups’ learning needs.

*When we practiced on our own, we felt stuck, but when the professor held our hands and placed the needles, the needles really went in, so I think if the professor helped us more at the side, we would have gotten better at placing the needles. (Student 1)*

Fourth, strategies to facilitate pre-class learning are required. Students watched instructional videos and filled out practice notes as pre-class learning activities. These pre-class learning activities were not included in the assessment score, and students were encouraged to learn on their own. However, students who lack self-directed learning skills may perform poorly in pre-class learning, so it is necessary to develop special strategies such as quizzes and rewards for pre-class learning. The volume of pre-class learning should be adjusted so that students do not feel overburdened, but pre-class learning should be encouraged by promoting students’ internal or external motivation.

*I think if there was an assignment to make sure that everyone had done their pre-class learning, it would have made the practice go smoother (...) Sometimes I couldn’t watch the video and I was a little bit unprepared, so I wish there was something to do pre-class learning before the practice. (Student 5)*

One respondent said of peer OSCE: “There were a lot of things I didn’t realize, and I was able to get feedback on a lot of things from a peer’s perspective, which helped me fill in the gaps.” (Student 1). The practice notes they recorded after classes were helpful because students were provided with “an objective evaluation of their performance, communication, and attitude” (Student 2) by their peers, and the feedback and reflection helped them understand what they needed to improve (S5 File).

### Data integration

**Fig 4:**
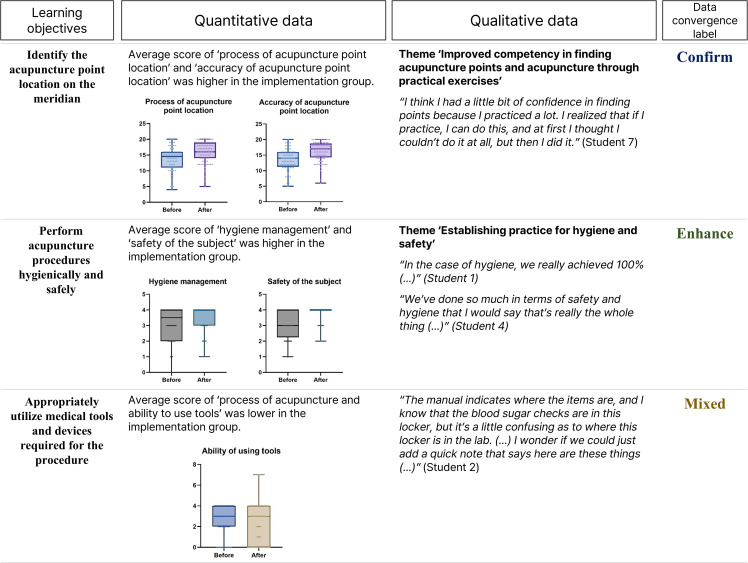
Integration of quantitative and qualitative findings by learning objectives.

Integration of quantitative and qualitative data revealed convergent, enhancing, and mixed findings across the three main objectives of the training program (Fig 4). First, the ability to ‘identify the acupuncture point location on the meridian’ was confirmed to be achieved based on both in the survey and interview. Second, improvements were seen in the outcomes on ‘performing acupuncture procedures hygienically and safely’, indicating that the program was effective in teaching students to perform acupuncture in hygienic and safe ways. Third, the findings on the ‘ability to use tools and devices’ were mixed, with neutral responses and small score differences.

## Discussion

This study aimed to provide an in-depth understanding of the implementation methods, effectiveness, and future improvements of a novel acupuncture training program in South Korea. The integrated findings show that the program is especially effective for performing hygienic and safe acupuncture. It is the first study to evaluate the effectiveness of a program developed using an instructional systems design model in undergraduate KM education. The findings of this study may provide an essential reference for future acupuncture training curricula.

The program included several strategies: flipped learning, a 5-step model for teaching procedural skills, peer role-play, and peer OSCE. Flipped learning is necessary for efficient skill training and hands-on practice in intensive healthcare curricula [22]. Our pre-class learning videos focused on finding acupuncture point locations, clean needle techniques, and acupuncture procedures. The survey results indicate overall satisfaction with the flipped classroom. In class, George’s 5-step model was used except for step 4 (“Students talk through the skill”) [14] to account for insufficient practice time. Breaking down each part of a skill into smaller units and teaching them sequentially in structured steps [23] has been effective in teaching clinical skills in KM education [24,25].

In acupuncture training, practice with human subjects is essential because using a model provides limited authenticity [26]. Therefore, we established groups comprising students as patients and observers to ensure adherence to procedures. This role-playing experience encouraged students to engage in self-reflection. We used peer OSCE to relieve anxiety associated with OSCE and to allow eight groups to work simultaneously with only one teacher [27]. Our interviews suggest that peer OSCE enables students to “fill the gaps” from peers’ viewpoints. This supplements traditional apprenticeship methods for skill acquisition by learning from both teachers and peers [28].

Another major benefit of the program is that it could enhance students’ communication skills. Communication skills are essential in acupuncture practice as practitioners are required to effectively explain procedures, obtain informed consent, provide reassurance during treatment, and establish therapeutic relationships with patients. Since inadequate communication between students in physician and patient roles emerged as a major performance concern in our ‘analysis’ phase, we evaluated communication self-efficacy at the beginning and end of the semester. Total communication self-efficacy scores improved after the course. Notable improvements were seen particularly in three areas: encouraging the patient to express thoughts and feelings, structuring the conversation with the patient, and checking patient’s understanding of the information given. These specific enhancements indicate that patient-physician role-play effectively develops essential communication skills required throughout the overall clinical encounter [29]. The mean total score (88.89 ± 13.27) in our study was similar to those measured in a previous study of senior students at the same university who had clinical practice training by peer role-play (89.37 ± 15.22) [30]. This is because students learn to communicate respectfully through role-playing [31,32], which becomes habitual, and they continually reflect on their attitudes.

The significant improvements in practical examination scores, particularly in point location accuracy and communication skills, align directly with the core learning objectives of our designed program using RPISD model. The convergent findings (Fig 4), where quantitative improvements correspond with qualitative reports of enhanced point location competency and safety practices, suggest these changes reflect systematic training effects rather than random variation. While different student cohorts in this study present a limitation, the specific pattern of improvements in areas directly targeted by our structured training approach supports the program’s effectiveness.

Student satisfaction survey revealed specific strengths and areas for improvement in program implementation. High satisfaction rates for learning material usefulness (91.1%) and appropriateness of learning activities (93.4%) indicate that the designed content effectively met students’ educational needs. The positive responses to peer OSCE activities (97.7%) and communication quality (84.5% for instructor-student, 88.9% for student-student) validate the collaborative learning approach in our design.

Notably, students did not have sufficient time to practice. This finding is supported by satisfaction scores for time allocation during different class phases, where only 42.2% of students found the introduction session time adequate, while 20% felt the group exercises and wrap-up time was insufficient. To address this issue, teachers’ demonstrations and explanations must be completed within the allocated time. As some students felt hands-on training in a group was more effective than demonstrations to the whole class, demonstration and hands-on practice should be balanced.

While most students indicated that the content alignment between pre-class and classroom learning was appropriate and enhanced their understanding of the classroom practice, 26.6% responded that the average class time for online learning (typically over one hour per session) was inadequate. Furthermore, 15.6% found the learning videos insufficiently engaging to facilitate learning, and interviews revealed similar concerns about pre-class motivation. These responses suggests that practical content should be redesigned into shorter microlearning modules to reduce student burden while enhancing engagement [33]. Future designs should segment content into 5–10 minute modules focusing on anatomical regions, where each video demonstrates point location techniques for related acupoints within the same body area, with interactive gamified quizzes between modules to reinforce learning and reduce cognitive burden [34]. This approach aligns with evidence showing microlearning effectiveness for novel skill acquisition [35].

Although this study provides valuable insights into the effectiveness and areas for improvement of a novel acupuncture training program, its limitations are acknowledged. A limitation of the study is that the effectiveness of the training program was evaluated using different student cohorts. Accordingly, future studies should follow the same cohort of students to more accurately examine whether individual students’ procedural competencies genuinely improve as a result of the program. Furthermore, future longitudinal studies should investigate long-term skill retention and clinical application of the competencies acquired through this training program. Follow-up assessments during students’ clinical rotations would provide valuable evidence regarding the transfer of learned skills to actual patient care settings.

Despite these limitations, this study presents an educational model and strategies that can be utilized in acupuncture curricula and general clinical skills training. It is recommended that experts and stakeholders review the validity of this model. Objective evaluations of students’ performance would be required. As acupuncture continues to gain global acceptance, establishing standardized yet culturally appropriate training methodologies becomes increasingly important. This study demonstrates that systematic instructional design can enhance traditional teaching methods without compromising their essence. Further research is needed to assess the feasibility and effectiveness of the program when applied to other regions and professions that use acupuncture.

## Conclusion

This novel acupuncture training program supports the development of core competencies for acupuncture in clinical practice. However, the primary contribution of this study lies not in demonstrating effectiveness per se, but in detailing how an acupuncture curriculum was systematically designed, implemented, and iteratively refined using the RPISD framework. Through surveys and in-depth interviews, we demonstrated that the program facilitated the establishment of hygiene and safety practices and enhanced students’ communication skills and competency in locating acupuncture points. The design principles and implementation insights presented in this study may be useful for acupuncturists, traditional Chinese medicine practitioners, and conventional physicians who require additional training in acupuncture.

## Supporting information

S1 File Lesson plans and manuals for teachers and students. (DOCX)

S2 File Survey questionnaire and script of semi-structured interview. (DOCX)

S3 File Survey data. (XLSX)

S4 File Practical examination score. (XLSX)

S5 File Interview transcript. (DOCX)

## References

[1] Zhuang Y, Xing J, Li J, Zeng B-Y, Liang F. History of acupuncture research. Int Rev Neurobiol. 2013;111:1–23. doi: 10.1016/B978-0-12-411545-3.00001-8 24215915

[2] Lu L, Zhang Y, Ge S, Wen H, Tang X, Zeng JC, et al. Evidence mapping and overview of systematic reviews of the effects of acupuncture therapies. BMJ Open. 2022;12(6):e056803. doi: 10.1136/bmjopen-2021-056803 35667716 PMC9171228

[3] Robinson N, Lorenc A, Ding W, Jia J, Bovey M, Wang X-M. Exploring practice characteristics and research priorities of practitioners of traditional acupuncture in China and the EU-A survey. J Ethnopharmacol. 2012;140(3):604–13. doi: 10.1016/j.jep.2012.01.052 22338645

[4] Bleck R, Marquez E, Gold MA, Westhoff CL. A scoping review of acupuncture insurance coverage in the United States. Acupunct Med. 2021;39(5):461–70. doi: 10.1177/0964528420964214 33307728

[5] Hou Z, Sun Z. The grass is always greener: a critical look at the lessons and challenges of acupuncture education in America. Front Med (Lausanne). 2025;12:1556346. doi: 10.3389/fmed.2025.1556346 40330785 PMC12052708

[6] Smith CL, Reddy B, Wolf CM, Schnyer RN, St John K, Conboy L, et al. The State of 21st Century Acupuncture in the United States. J Pain Res. 2024;17:3329–54. doi: 10.2147/JPR.S469491 39403098 PMC11472758

[7] Kim D, Shih C-C, Cheng H-C, Kwon S-H, Kim H, Lim B. A comparative study of the traditional medicine systems of South Korea and Taiwan: Focus on administration, education and license. Integr Med Res. 2021;10(3):100685. doi: 10.1016/j.imr.2020.100685 33665088 PMC7903058

[8] Kim M, Han C. A Survey on the Educational Status of Basic Korean Medicine and Basic Medical Science in Colleges of Korean Medicine in 2020. J Korean Med. 2020;41(3):98–124. doi: 10.13048/jkm.20028

[9] Won J, Lee J-H, Bang H, Lee H. Safety of acupuncture by Korean Medicine Doctors: a prospective, practice-based survey of 37,490 consultations. BMC Complement Med Ther. 2022;22(1):300. doi: 10.1186/s12906-022-03782-z 36401264 PMC9675262

[10] Cho E, Kim J-H, Hong J. Application of the Rapid Prototyping Instructional Systems Design in Meridianology Laboratory. Korean J Acupunct. 2022;39(3):71–83. doi: 10.14406/acu.2022.012

[11] Cho E, Hong J, Nam Y, Shin H, Kim J-H. Development of Teaching and Learning Manual for Competency-Based Practice for Meridian & Acupuncture Points Class. Korean J Acupunct. 2022;39(4):184–90. doi: 10.14406/acu.2022.023

[12] Lim C, Song Y, Hong S, Park C. A Study on the Application and Improvement of the Rapid Prototyping to Instructional Systems Design (RPISD) Model. JOET. 2020;36(3):589–617. doi: 10.17232/kset.36.3.589

[13] Lim C, Yeon E. Development of corporate training programs and instructional systems design. Paju: Kyoyookbook. 2015.

[14] George JH, Doto FX. A simple five-step method for teaching clinical skills. Fam Med. 2001;33(8):577–8. 11573712

[15] Iqbal MdH, Siddiqie SA, Mazid MdA. Rethinking theories of lesson plan for effective teaching and learning. Social Sciences & Humanities Open. 2021;4(1):100172. doi: 10.1016/j.ssaho.2021.100172

[16] Smidt A, Balandin S, Sigafoos J, Reed VA. The Kirkpatrick model: A useful tool for evaluating training outcomes. J Intellect Dev Disabil. 2009;34(3):266–74. doi: 10.1080/13668250903093125 19681007

[17] Lee S. Development of evaluation scale of flipped-learning course operation suitability in a university. The Korean Journal of Educational Methodology Studies. 2018;30:1–20. doi: 10.17927/tkjems.2018.30.2.1

[18] Lee S. “Dimension Classification and Questionnaire Design Criteria for Reaction Evaluation of Educational/Training Programs”. KSET. 2005;21(3):187–214. doi: 10.17232/kset.21.3.187

[19] Gil CR, Sung KM. Validity and reliability of the Korean version of self-efficacy questionnaire (KSE-12). Journal of Digital Convergence. 2020;18:337–45. doi: 10.14400/JDC.2020.18.5.337

[20] Axboe MK, Christensen KS, Kofoed P-E, Ammentorp J. Development and validation of a self-efficacy questionnaire (SE-12) measuring the clinical communication skills of health care professionals. BMC Med Educ. 2016;16(1):272. doi: 10.1186/s12909-016-0798-7 27756291 PMC5069791

[21] Braun V, Clarke V. Using thematic analysis in psychology. Qualitative Research in Psychology. 2006;3(2):77–101. doi: 10.1191/1478088706qp063oa

[22] Zhang W, Gu J, Li F, Feng F, Chen H, Xing X, et al. The effect of flipped classroom in multiple clinical skills training for clinical interns on Objective Structured Clinical Examinations (OSCE). Med Educ Online. 2022;27(1):2013405. doi: 10.1080/10872981.2021.2013405 34898400 PMC8676640

[23] Burgess A, van Diggele C, Roberts C, Mellis C. Tips for teaching procedural skills. BMC Med Educ. 2020;20(Suppl 2):458. doi: 10.1186/s12909-020-02284-1 33272273 PMC7712522

[24] Cho E, Han Y-M, Kang Y, Kim J-H, Shin M-S, Oh M, et al. Implementation of Objective Structured Clinical Examination on Diagnostic Musculoskeletal Ultrasonography Training in Undergraduate Traditional Korean Medicine Education: An Action Research. Diagnostics (Basel). 2022;12(7):1707. doi: 10.3390/diagnostics12071707 35885609 PMC9323213

[25] Cho E, Jeon H, Kwon OS, Hong J, Lee J, Jung E, et al. Training future Korean medicine doctors to perform bee venom acupuncture and obtain informed consent using an objective structured clinical examination. J Korean Med. 2022;43(1):6–17. doi: 10.13048/jkm.22002

[26] Cho E, Lee J-H, Kwon OS, Hong J, Cho NG. Assessment of acupuncture and moxibustion medicine clinical practice using the objective structured clinical examination. J Acupunct Res. 2021;38:219–26. doi: 10.13045/jar.2021.00122

[27] Young I, Montgomery K, Kearns P, Hayward S, Mellanby E. The benefits of a peer-assisted mock OSCE. The Clinical Teacher. 2014;11:214–8. 24802924 10.1111/tct.12112

[28] Zhou X, Yang Q, Bi L, Wang S. Integrating traditional apprenticeship and modern educational approaches in traditional Chinese medicine education. Med Teach. 2024;46(6):792–807. doi: 10.1080/0142159X.2023.2284661 38052086

[29] Haq C, Steele DJ, Marchand L, Seibert C, Brody D. Integrating the art and science of medical practice: innovations in teaching medical communication skills. Fam Med. 2004;36 Suppl:S43–50. 14961402

[30] Cho E, Jung H-J, Leem J. Peer Role-Play in a College of Korean Medicine to Improve Senior Students’ Competencies in Patient Care and Communication: A Case Analysis and Proposal for a Model. J Korean Med. 2022;43(3):49–60. doi: 10.13048/jkm.22030

[31] Nestel D, Tierney T. Role-play for medical students learning about communication: guidelines for maximising benefits. BMC Med Educ. 2007;7:3. doi: 10.1186/1472-6920-7-3 17335561 PMC1828731

[32] Bosse HM, Nickel M, Huwendiek S, Schultz JH, Nikendei C. Cost-effectiveness of peer role play and standardized patients in undergraduate communication training. BMC Med Educ. 2015;15:183. doi: 10.1186/s12909-015-0468-1 26498479 PMC4619415

[33] Fidan M. The effects of microlearning-supported flipped classroom on pre-service teachers’ learning performance, motivation and engagement. Educ Inf Technol (Dordr). 2023;1–28. doi: 10.1007/s10639-023-11639-2 37361810 PMC10011779

[34] Abdulmajed H, Park YS, Tekian A. Assessment of educational games for health professions: a systematic review of trends and outcomes. Med Teach. 2015;37 Suppl 1:S27–32. doi: 10.3109/0142159X.2015.1006609 25803590

[35] De Gagne JC, Park HK, Hall K, Woodward A, Yamane S, Kim SS. Microlearning in Health Professions Education: Scoping Review. JMIR Med Educ. 2019;5(2):e13997. doi: 10.2196/13997 31339105 PMC6683654

