# Supplementary material for: Implementing a competency-based acupuncture training program in Korean Medicine education
Source: PLoS One. 2026 Mar 20;21(3):e0345289. doi: 10.1371/journal.pone.0345289 (PMC13004403; doi:10.1371/journal.pone.0345289)
Supplement: S1 File — (DOCX) [file pone.0345289.s001.docx]

**Curriculum of the 1-year acupuncture training program**

| Week | Contents in the 1st semester | Contents in the 2nd semester |
| --- | --- | --- |
| 1 | Introduction of the class | Clean needle technique and needling techniques |
| 2 | Clean needle technique and needling techniques | Measuring vital signs |
| 3 | Measuring vital signs | Locating acupuncture points and Needling: Pericardium Meridian |
| 4 | Acupuncture technique: Moxibustion | Locating acupuncture points and Needling: Triple Energizer Meridian |
| 5 | Locating acupuncture points and Needling: Lung Meridian | Locating acupuncture points and Needling: Governor Vessel and Bladder Meridian (1) |
| 6 | Locating acupuncture points and Needling: Large Intestine Meridian | Locating acupuncture points and Needling: Governor Vessel and Bladder Meridian (2) |
| 7 | Locating acupuncture points and Needling: Conception Vessel (1) | Locating acupuncture points and Needling: Governor Vessel and Bladder Meridian (3) |
| 8 | Regular exam period (self-directed learning) | Regular exam period (self-directed learning) |
| 9 | Locating acupuncture points and Needling: Conception Vessel (2) | Locating acupuncture points and Needling: Kidney Meridian  Acupuncture technique: Warm needling |
| 10 | Locating acupuncture points and Needling: Stomach Meridian (1) | Locating acupuncture points and Needling: Gallbladder Meridian (1) |
| 11 | Locating acupuncture points and Needling: Stomach Meridian (2) | Acupuncture technique: Wet & Dry cupping |
| 12 | Acupuncture technique: Electroacupuncture | Acupuncture technique: Scalp acupuncture |
| 13 | Locating acupuncture points and Needling: Spleen Meridian | Locating acupuncture points and Needling: Gallbladder Meridian (2) |
| 14 | Locating acupuncture points and Needling: Heart Meridian | Locating acupuncture points and Needling: Liver Meridian |
| 15 | Locating acupuncture points and Needling: Small Intestine Meridian | Acupuncture technique: Auricular acupuncture |
| 16 | Practical examination | Practical examination |

Each class lasts for three hours.

**1. Acupuncture practice lesson plan**

**1) Clean needle technique and needling techniques**

| **Subject name** | | Meridian & Acupoint Practice | | | |
| --- | --- | --- | --- | --- | --- |
| **Target** | | Korean Medicine (KM) students (first and second year) | | | |
| **Lesson topics** | | - Infection prevention, acupuncture safety, clean needle technique, needling techniques, needling practice | | | |
| **Class types** | | Practice (learn locating acupuncture points and needling techniques in small group) | | | |
| **Learning objectives** | | - Perform acupuncture procedures hygienically and safely. - Properly utilize the medical tools and devices required for the procedure. - Record precisely what has been done during procedures. | | | |
| **Teaching methods** | | - Practice - Flipped learning | | | |
| **Flow of lessons** | | | | | |
| **Steps** | | **Instructor** | **Student** | **Learning time**  **(minutes)** | **Manual** |
| Before class | | - Utilize Google Classroom to upload video lectures and make them accessible to students | - Access Google Classroom and watch the video lectures. - Record the learning contents in groups (clean needle technique, skin disinfection, needle technique, overview of needling and needling process) in a practice note. | - | #Practice note  #Rubric for grading practice note |
| In class | Introduction | - Show group seating arrangements on the monitor | - Enter the lab and change into lab coats in the locker room. | 15 | #Positioning example  #Examples of lab coats |
|  |  |  | - Wash hands at a sink. |  | #Hand washing protocol |
|  |  |  | - Cover a bedcover on bed. |  | #How to use bedcover |
|  |  |  | - Bring group supplies to bed. |  | #Supply user guide |
|  |  |  | - Every student completes the Safety checklist for preparing for practice and acupuncture procedures (using themselves as the patient). |  | #Pre-procedure checklist |
|  |  |  | - Prepare to record the practice note collaboratively with group members at the worktable. |  |  |
|  |  | - Present learning objectives |  | 2 |  |
|  |  | - Attract attention (meditation) |  | 3 |  |
|  | Development | Instructor presentation   - Explain key aspects of the clean needle technique, including how to use the cart and how to distinguish between clean and contamination areas. - Needling practice tools, procedure guides (including how to hold the needle, how to measure needle depth, and how to use items) - Live stream the demonstration on screen. |  | 30 |  |
|  |  | - Break | | 10 |  |
|  |  |  | - Bring supplies for the practice (alcohol swabs, latex gloves, etc.). | 60 |  |
|  |  | - Look around each group and coach them through the practice, making sure they're doing a good job of - Related to clean needle technique: handwashing, cart use, and clean/contamination area distinction - Related to needling techinque: needle holding position, piercing the epidermis quickly | Group practice   - All students practice needling with materials, and after sufficient practice, practice on their own body. - Each person performs the entire needling procedure without assistance from another member of the group and records their needling practice in a practice note. - Check own hygiene and acupuncture safety during the practice and record them in the practice note. |  | #Conversation guideline during group practice |
|  |  | - Teaching assistants are assigned groups of students to observe their performance and provide feedback. - Demonstrate the procedure if a student asks. |  |  |  |
|  | Wrap-up |  | - Every student should complete <Post-procedure Checklist>. |  | #Post-procedure checklist |
|  |  |  | - Organize supplies in groups, record group supply logs. |  |  |
|  |  | - Check cleaning status | - Sort and discard wastes. |  | #Waste disposal manual |
|  |  |  | - Clean up around. |  |  |
|  |  |  | - The group in charge of cleaning get instructor confirmation after completing cleaning. |  | #Cleaning manual  #Lab cleanup manual |
|  |  |  | - After the practice, get instructor confirmation. |  |  |
|  |  |  | - Change clothes in the locker room and leave. |  |  |
| **Tools** | | | | | |
| Google classroom, Powerpoint, Word | | | | | |
| **Keep in mind** | | | | | |
| - Groups of 4 to 6 people. - All students practice needling on themselves, regardless of whether they are the physician, patient, or observer. | | | | | |

**2) Measuring vital signs**

| **Subject name** | | Meridian & Acupoint Practice | | | |
| --- | --- | --- | --- | --- | --- |
| **Target** | | Korean Medicine (KM) students (first and second year) | | | |
| **Lesson topics** | | - Measuring blood pressure, body temperature, and blood glucose | | | |
| **Class types** | | Practice (learn locating acupuncture points and needling techniques in small group) | | | |
| **Learning objectives** | | - Measure the vital signs of the patient. - Properly utilize the medical tools and devices required for the procedure. - Record precisely what has been done during procedures. | | | |
| **Teaching methods** | | - Practice - Flipped learning | | | |
| **Flow of lessons** | | | | | |
| **Steps** | | **Instructor** | **Student** | **Learning time**  **(minutes)** | **References** |
| Before class | | - Utilize Google Classroom to upload video lectures and make them accessible to students | - Access Google Classroom and watch the videos. - Record vital signs measurements in groups in a practice note. - Establish physician-patient roles so that every group member experiences both physician and patient roles during blood pressure, body temperature, and blood glucose measurement. | - | #Practice note  #Rubric for grading practice note |
| In class | Introduction | - Show group seating arrangements on the monitor | - Enter the lab and changing into lab coats in the locker room. | 15 | #Positioning example  #Examples of lab coats |
|  |  |  | - Wash hands at a sink. |  | #Hand washing protocol |
|  |  |  | - Cover a bedcover on bed. |  | #How to use bedcover |
|  |  |  | - Bring group supplies to bed. |  | # Supply user guide |
|  |  |  | - Students should explain the contents of the <Acupuncture practice consent form> to the patient and obtain their consent. |  | #Acupuncture practice consent form |
|  |  |  | - Every student should complete the Safety checklist for preparing for practice and acupuncture procedures. |  | #Pre-procedure checklist |
|  |  |  | - Prepare to record the practice note collaboratively with group members at the worktable. |  |  |
|  |  | - Present learning objectives |  | 2 |  |
|  |  | - Attract attention (meditation) |  | 3 |  |
|  | Development | Instructor presentation   - Demonstrate blood pressure/body temperature/blood glucose measurement process. - Live stream the demonstration on screen. |  | 30 | #Detailed lesson plan (PT) |
|  |  | - Break | | 10 |  |
|  |  |  | - Bring supplies for the practice (alcohol swabs, latex gloves, etc.). | 50 |  |
|  |  | - Look around each group to ensure the practice procedures are working well. | Group practice   - The patient lies on the bed. - Measure vital signs by referring to the vital signs measurement method you recorded before the practice. - When not acting as a physician or patient, take on the role of observer. Observe the physician's performance, monitor hygiene and safety procedures, and provide feedback. - Each physician performs the entire vital signs measurement process without the assistance of another group members, and records the process, vital signs measurement results, and feedback received in the practice note. |  | #Conversation guideline during group practice |
|  |  | - Teaching assistants are assigned groups of students to observe their performance and provide feedback. - Demonstrate the procedure if a student asks. |  |  |  |
|  |  |  |  |  |  |
|  | Wrap-up |  | - Every student should complete <Post-procedure Checklist>. |  | #Post-procedure checklist |
|  |  |  | - Organize supplies in groups, record group supply logs. |  |  |
|  |  | - Check cleaning status | - Sort and discard wastes. |  | #Waste disposal manual |
|  |  |  | - Clean up around. |  |  |
|  |  |  | - The group in charge of cleaning get instructor confirmation after completing cleaning. |  | #Cleaning manual  #Lab cleanup manual |
|  |  |  | - After the practice, get instructor confirmation. |  |  |
|  |  |  | - Change clothes in the locker room and leave. |  |  |
| **Tools** | | | | | |
| Google classroom, Powerpoint, Word | | | | | |
| **Keep in mind** | | | | | |
| - Groups of 4 to 6 people. - Measure blood pressure/body temperature/blood glucose. Each group member should take on the roles of physician, patient, and observer in turn. The observer is the one who does not take on the roles of physician or patient. | | | | | |

**3) Locating acupuncture points and Needling**

| **Subject name** | | Meridian & Acupoint Practice | | | |
| --- | --- | --- | --- | --- | --- |
| **Target** | | Korean Medicine (KM) students (first and second year) | | | |
| **Lesson topics** | | - Acupuncture points on the 14 meridian | | | |
| **Class types** | | Practice (learn locating acupuncture points and needling techniques in small group) | | | |
| **Learning objectives** | | - Measure the vital signs of the patient. - Locate the acupuncture point of the corresponding meridian. - Perform acupuncture procedures hygienically and safely. - Properly utilize the medical tools and devices required for the procedure. - Record precisely what has been done during procedures. | | | |
| **Teaching methods** | | - Practice - Flipped learning | | | |
| **Flow of lessons** | | | | | |
| **Steps** | | **Instructor** | **Student** | **Learning time**  **(minutes)** | **References** |
| Before class | | - Utilize Google Classroom to upload video lectures and make them accessible to students | - Access Google Classroom and watch the videos - Record the locating procedure for acupuncture points in the group's practice note. | - | #Practice note  #Example of practice note  #Rubric for grading practice note |
|  |  |  | - Distribute the roles of physician, patient, and observer for the practice. | - | #Each role manual |
| In class | Introduction | - Show group seating arrangements on the monitor | - Enter the lab and changing into lab coats in the locker room. | 15 | #Positioning example  #Examples of lab coats |
|  |  |  | - Wash hands at a sink. |  | #Hand washing protocol |
|  |  |  | - Cover a bedcover on bed. |  | #How to use bedcover |
|  |  |  | - Bring group supplies to bed. |  | #Supply user guide |
|  |  |  | - Students should explain the contents of the <Acupuncture practice consent form> to the patient and obtain their consent (If the consent form is not completed, the practice will be stopped). |  | #Acupuncture practice consent form |
|  |  |  | - Measure the patient’s blood pressure, body temperature, and blood glucose. |  |  |
|  |  |  | - Observer completes a pre-procedure checklist. |  | #Pre-procedure checklist |
|  |  |  | - Prepare to record the practice note collaboratively with group members at the worktable. |  |  |
|  |  | - Present learning objectives |  | 2 |  |
|  |  | - Attract attention (meditation) |  | 3 |  |
|  | Development | Instructor presentation   - Select acupuncture points - Demonstrate locating acupuncture points - Live stream the demonstration on screen. |  | 30 | #Detailed lesson plan (PT) |
|  |  | - Break | | 10 |  |
|  |  |  | - Bring supplies for the practice (alcohol swabs, latex gloves, etc.). | 100 |  |
|  |  | - Look around each group to ensure the practice procedures are working well. | Group practice   - The patient lies on the bed. - The physician should refer to the locating procedure documented before the practice to locate acupuncture points. - Each physician performs the entire needling process without the assistance of another group members and records their performance in the practice note. - The observer monitors the physician's performance, check for hygiene and safety procedures, and record them in the practice note. |  | #Conversation guideline during group practice |
|  |  | - TAs are assigned groups of students to observe their performance and provide feedback. - Demonstrate the procedure if a student asks. |  |  |  |
|  |  | - Present OSCE instruction (including acupuncture points) | - At the end of the practice session, each physician performs the acupuncture procedure on the acupuncture points suggested by the instructor one time. The observer assesses and provides feedback to the physician according to the OSCE checklist. - The observer records OSCE results in practice note. |  | #OSCE checklist (needling procedures) |
|  | Wrap-up |  | - Every student should complete <Post-procedure checklist>. |  | #Post-procedure checklist |
|  |  |  | - Organize supplies in groups, record group supply logs. |  |  |
|  |  | - Check cleaning status | - Sort and discard wastes. |  | #Waste disposal manual |
|  |  |  | - Clean up around. |  |  |
|  |  |  | - The group in charge of cleaning get instructor confirmation after completing cleaning. |  | #Cleaning manual  #Lab cleanup manual |
|  |  |  | - After the practice, get instructor confirmation. |  |  |
|  |  |  | - Change clothes in the locker room and leave. |  |  |
| **Tools** | | | | | |
| Google classroom, Powerpoint, Word | | | | | |
| **Keep in mind** | | | | | |
| - Groups of 4 to 6 people. - Measure blood pressure/body temperature/blood glucose. Each group member should take on the roles of physician, patient, and observer in turn. The observer is the one who does not take on the roles of physician or patient. | | | | | |

**4) Acupuncture techniques**

| **Subject name** | | Meridian & Acupoint Practice | | | |
| --- | --- | --- | --- | --- | --- |
| **Target** | | Korean Medicine (KM) students (first and second year) | | | |
| **Lesson topics** | | - Acupuncture and Moxibustion procedure (moxibustion/warm needling/cupping/electroacupuncture/scalp acupuncture/ear acupuncture) | | | |
| **Class types** | | Practice (learn locating acupuncture points and needling techniques through small group practice) | | | |
| **Learning objectives** | | - Measure the vital signs of the patient. - Perform acupuncture procedures hygienically and safely. - Properly utilize the medical tools and devices required for the procedure. - Record precisely what has been done during procedures. | | | |
| **Teaching methods** | | - Practice - Flipped learning | | | |
| **Flow of lessons** | | | | | |
| **Steps** | | **Instructor** | **Student** | **Learning time**  **(minutes)** | **References** |
| Before class | | - Utilize Google Classroom to upload video lectures and make them accessible to students | - Access Google Classroom and watch the videos. - Record the process, indicants, and precautions for the procedures in a practice note. |  | #Practice note  #Rubric for grading practice note |
|  |  |  | - Distribute the roles of physician, patient, and observer for the practice. |  | #Each role manual |
| In class | Introduction | - Show lab group seating arrangements on the monitor | - Enter the lab and change into lab coats in the locker room. | 15 | #Positioning example  #Examples of lab coats |
|  |  |  | - Wash hands at a sink. |  | #Hand washing protocol |
|  |  |  | - Cover a bedcover on bed. |  | #How to use bedcover |
|  |  |  | - Bring group supplies to Bed. |  | #Supply user guide |
|  |  |  | - Students should explain the contents of the <Acupuncture practice consent form> to the patient and obtain their consent (If the consent form is not completed, the practice will be stopped). |  | #Acupuncture practice consent form |
|  |  |  | - Measure the patient’s blood pressure, body temperature, and blood glucose. |  |  |
|  |  |  | - The observer complete a pre-procedure checklist. |  | #Pre-procedure checklist |
|  |  |  | - Prepare to record the practice note collaboratively with group members at the worktable. |  |  |
|  |  | - Present learning objectives |  | 2 |  |
|  |  | - Attract attention (meditation) |  | 3 |  |
|  | Development | Instructor presentation   - Demonstrate the whole procedure - Show without explanation, then repeat while explaining the same process. - Live stream the demonstration on your screen. |  | 30 | #Detailed lesson plan (PT) |
|  |  | - Break | | 10 |  |
|  |  |  | - Bring supplies for the practice (alcohol swabs, latex gloves, etc.). | 100 |  |
|  |  | - Look around each group to ensure the practice procedures are working well. | Group practice   - The patient lies on the bed. - Perform the entire procedure without the assistance of another group members, referring to the procedure documented before the practice, and record the procedure in the practice note. - The observer monitors the physician's performance, check for hygiene and safety procedures, and record them in the practice note. |  | #Conversation guideline during practice |
|  |  | - Teaching assistants are assigned groups of students to observe their performance and provide feedback. - Demonstrate the procedure if a student asks. |  |  |  |
|  |  | - Present OSCE instruction (including acupuncture points) | - At the end of the practice session, each physician performs the procedure one time. The observer assesses and provides feedback to the physician according to the OSCE checklist. - The observer records OSCE results in practice note. |  | #OSCE checklist |
|  | Wrap-up |  | - Every student should complete <Post-procedure checklist>. |  | #Post-procedure checklist |
|  |  |  | - Organize supplies in groups, record group supply logs. |  |  |
|  |  | - Check cleaning status | - Sort and discard wastes. |  | #Waste disposal manual |
|  |  |  | - Clean up around. |  |  |
|  |  |  | - The group in charge of cleaning get instructor confirmation after completing cleaning. |  | #Cleaning manual  #Lab cleanup manual |
|  |  |  | - After the practice, get instructor confirmation. |  |  |
|  |  |  | - Change clothes in the locker room and leave. |  |  |
| **Tools** | | | | | |
| Google classroom, Powerpoint, Word | | | | | |
| **Keep in mind** | | | | | |
| - Groups of 4 to 6 people. - Measure blood pressure/body temperature/Blood glucose. Each group member should take on the roles of physician, patient, and observer in turn. The observer is the one who does not take on the roles of physician or patient. | | | | | |

**2. Acupuncture practice consent form**

**Practice date**

|  |
| --- |

**Practice topics and scope**

Briefly describe the practice topic for the day and the scope of the group practice.

|  |
| --- |

**Objective, definition, and process of acupuncture practice**

(Objective) This consent form is intended to obtain informed consent for the exposure, contact, and treatment of the body of the patient in the acupuncture practice, a basic training course to train undergraduate students majoring Korean medicine on a non-profit basis, and to understand and resolve situations expected of the physician in the process of achieving the objective of the training.

□ Confirmed

(Definitions) The following terms have the following meanings 1. "Instructor" means a professor of the Meridian & Acupoint subject who is a licensed Korean medicine doctor, or a person delegated by the professor as the person in charge of the practice. 2. "Assistant" means a person who assists in the preparation and conduct of the practice under the guidance of the practice supervisor in the practice and is a graduate of a graduate course or degree in Meridian & Acupoint (acupuncture) at a college of Korean medicine or equivalent. 3. "Physician" refers to a student who is taking the acupuncture practice course and detects or treats acupuncture points on the body of the patient with the consent of the patient. 4. "Patient" refers to a student who is taking the acupuncture practice and has given prior consent to the locating acupuncture points or acupuncture procedure on the body during the practice in accordance with the procedures defined in this document. 5. "Observer" refers to a student who is taking the acupuncture training program and observes the process and technique of the physician and the patient during the practice.

□ Confirmed

(Practice course) Acupuncture practice consists of an introduction to the anatomical structure, bone proportional cun measurement, demonstration of locating acupuncture points according to surface anatomy and palpation, and group locating practice of acupuncture points. In the process of bone proportional cun measurement, superficial anatomy, palpation, and locating acupuncture points, there may be exposure, touching, and use of markers on the patient. However, with the consent of the patient, the instructor and assistant may optionally guide the practice of invasive Korean medicine procedures such as needling, moxibustion, and bloodletting for the purpose of education.

□ Confirmed

**Participant obligations and patient rights**

(Obligations of the instructor or physician) The instructor or physician is obligated to prepare the following to minimize the embarrassment and discomfort caused by the exposure and contact of the patient's body and to prevent adverse events and side effects of the training process. 1. sterilized practice supplies to prevent infection and medical supplies writerized for human use. 2. supplies and disinfecting instruments for the safety of the patient. e.g.) Forceps, stainless cans, alcohol swab, surgical gloves, disposable bedcover. 3. Efforts to minimize the exposure of the patient and related supplies. Examples.) Blankets, etc.

(Rights of the patient) Even if the practice is conducted according to the consent of the patient, the embarrassment and discomfort caused by the exposure and contact of the patient's body should be minimized, and if the rights of the patient are violated, the practice shall be stopped immediately upon the expression of the patient's intention or the instruction of the practice instructor and assistant.
1. If a "serious adverse event" presumed to be caused by the practice occurs, the practice instructor shall provide medical treatment and necessary first aid immediately upon receiving the report.
2. However, the physician shall be responsible for any serious adverse events caused by the practice and procedures performed at the physician's discretion, regardless of the instructions and consent of the practice instructor and assistants or the contents of the practice on the day.
3. All persons participating in acupuncture practice must complete the "Laboratory Safety Education" prescribed by Wonkwang University, and shall be legally protected and compensated for any damage caused in accordance with the regulations prescribed by Wonkwang University.
4. If the patient agrees to live broadcasting or recording through photography and video recording and is utilized as materials for public purposes for acupuncture education, personal identification information shall be protected from leakage.

(Adverse Event) The physician should follow the evidence in the Acupuncture Safety Guide (Acupuncture Safety Guide. Gunja Publishing House. 2011). Physicians should proceed safely with acupuncture practice under the guidance of a instructor or assistant. The physician should proceed with the practice under the guidance of the instructor and assistant, with the consent of the patient. However, the following minor and major adverse events may occur depending on the individual's physical reaction and the procedure.
1. "Minor adverse reactions (common)": Skin sensitization on contact, rash, redness, subcutaneous hemorrhage, transient pain after needling, mild faint during acupuncture treatment, muscle cramps, first-degree burns, stuck needle, etc.
2. "Serious adverse reactions (rare)": Infection (including cross-infection), vascular injury, localized hematoma, peripheral nerve injury, central nerve injury, pneumothorax, organ damage, chronic pain after needling, severe faint during acupuncture treatment, paralysis, loss of consciousness, third-degree burns, needle breakage, etc.

□ Confirmed

**Practice Group**

|  |
| --- |

**Consent to scope of body exposure for locating acupuncture points and procedures**

Check all areas where the patient has consented to expose. Unchecked areas are regarded as indicating that the patient does not consent.

□ Shoulder to elbow joint region: axillary, scapular, and brachial region

□ Hip to knee joints: femoral region

□ Anterior superior iliac spine to pubic symphysis: inguinal region

□ Sacrum to gluteal fold: gluteal region

□ Pectoral region (thoracic cavity)

□ Xiphisternal joint to umbilicus: upper abdominal region

□ Umbilicus to pubic symphysis: lower abdominal region

□ 7th cervical vertebra (C7) to 5th lumbar vertebra (L5): back and lumbar region

**Consent to body contacting the patient for locating acupuncture points and procedures**

Check all the areas where the patient has consented to body contact. Unchecked areas are regarded as indicating that the patient does not consent.

□ Head, face, and cervical area

□ Shoulder to elbow joint area: axilla, shoulder, and brachial region

□ Elbow joint to finger region

□ Hip to knee joints: femoral region

□ Knee joint to toe region

□ Anterior superior iliac spine to pubic symphysis: inguinal region

□ Sacrum to gluteal fold: gluteal region

□ Pectoral region (thoracic cavity)

□ Xiphisternal joint to umbilicus: upper abdominal region

□ Umbilicus to pubic symphysis: lower abdominal region

□ 7th cervical vertebra (C7) to 5th lumbar vertebra (L5): back and lumbar region

**Consent to procedures and techniques on the patient**

|  | Yes | No | N/A |
| --- | --- | --- | --- |
| Acupuncture needling | □ | □ | □ |
| Direct Moxibustion | □ | □ | □ |
| Indirect Moxibustion | □ | □ | □ |
| Bloodletting | □ | □ | □ |
| Cupping | □ | □ | □ |
| Electroacupuncture | □ | □ | □ |

**Other procedures and techniques that the patient consents to**

If encountering a procedure or technique in your acupuncture practice that is not included in the procedures and techniques section above, please describe it and whether or not the patient has consented.

**Filming for live streaming and recording practice video lecture**

Live broadcasting means that the practice scene is filmed in the meridian acupuncture laboratory and broadcasted in real time through the projector and monitor. However, this agreement does not include the consent of students to take photos of each other for portfolio and report.

|  | Yes | No |
| --- | --- | --- |
| Live in-lab broadcast | □ | □ |
| Record video | □ | □ |
| Use recorded video for in-school student teaching | □ | □ |

(Consent of the patient) The acupuncture practice is conducted for the educational purpose and learning effect of the students in the non-profit and public interest. In consideration of this, civil and criminal liability shall not be imposed on the instructor, assistant, and physician for any damage such as adverse reactions of the patient caused during the practice. In addition, I agree that the recorded footage will be utilized for non-profit and public interest purposes only under conditions that protect the privacy of the patients.

□ Confirmed

(Patient Consent) Each of the items expected during the acupuncture practice has been explained to the patient by the instructor or physician, and the patient has been fully informed of the above and consents to the practice.

□ Confirmed

**Practice participants name and signature**

Create a list of students participating in the practice by role.

**Physician list**

*Filling in your name replaces signing the practice consent form.

|  |
| --- |

**Patient list**

*Filling in your name replaces signing the practice consent form. If you have multiple patients, you'll need to create a new practice consent form if the scope of the patients' consent changes.

|  |
| --- |

**Observer list**

*Filling in your name replaces signing the practice consent form.

|  |
| --- |

**3. Pre-procedure acupuncture checklist**

When performing an acupuncture procedure, follow the checklist below.

**Practice group**

|  |
| --- |

**Absent/Late/Dismissed student names**
Ex) None or Yes (list absent:name/late:name/dismissed:name)

|  |
| --- |

**Patient name**

|  |
| --- |

**Complete the patient consent form**

□ Have completed the patient consent form.

□ The consent form has been reviewed and signed by the patient and physician.

**Patient's body temperature (in degrees Celsius)**

|  |
| --- |

**Patient's blood pressure (high/low)**

|  |
| --- |

**The patient's blood glucose (mg/dL) and time since eating**

|  |
| --- |

**Patient's health condition of the day**

Ask about the condition of your patient today or something else you need to consider.

|  |
| --- |

**Identifying symptoms to look out for in acupuncture patient**

□ Have a medical condition (hemophilia, leukemia, etc.) that affects my clotting function. I am taking medication that affects blood clotting (aspirin, etc.)

□ Have an allergy to metals.

□ Have a contagious or infectious disease.

□ Currently pregnant or may become pregnant.

□ Never had acupuncture before or have experienced side effects from acupuncture.

□ None of the above. Do not have any symptoms that the physician to be aware of regarding acupuncture.

**Check the health of the patient to watch out for during acupuncture**

□ Drunken alcohol or taken stimulant drugs and being under the influence of them.

□ Currently very angry, surprised, or afraid.

□ Extremely tired or have no energy.

□ Excessive bleeding or blood donation within 24 hours (for women, menstrual bleeding)

□ Sweating profusely due to overexercise or physical weakness.

□ None of the above. Do not have any conditions that require caution with acupuncture.

**Ensure physician hand washing and hygiene**

□ All physicians washed their hands according to the handwashing manual.

□ The physician's nails are clean.

□ The physician checked handwashing and hand hygiene routinely before and after the procedure.

**Check the physician's pre-needling preparation**

□ The physician checks the needle site for wounds or skin disease.

□ Use an alcohol swab to wipe in one direction or in a spiral outward motion and wait for it to dry.

□ Sanitize the physician's left hand (the hand that is not holding the needle) with an alcohol swab and palpate the area to be treated.

**Manage the patient's posture before the procedure**

□ Guide the patient into an appropriate and safe position for needling the acupuncture points.

□ Guide the patient, taking care not to change position during the procedure.

**4. Post-procedure checklist**

After each acupuncture procedure, verify compliance with the checklist below.

**Practice group**

|  |
| --- |

**Absent/Late/Dismissed Student Names**
Ex) None or Yes (list absent:name/late:name/dismissed:name)

|  |
| --- |

**Patient name**

|  |
| --- |

**Complete the patient consent form**

□ Filled out an informed consent form.

□ The informed consent form has been reviewed and signed by the patient and the physician.

**Patient's body temperature (in degrees Celsius)**

|  |
| --- |

**Patient's blood pressure (high/low)**

|  |
| --- |

**The patient's blood glucose (mg/dL) and time since eating**

|  |
| --- |

**Patient's health condition of the day**

Ask about the health of your patient today or something else you need to consider

|  |
| --- |

**Identifying symptoms to look out for in acupuncture patient**

□ Have a medical condition (hemophilia, leukemia, etc.) that affects my clotting function. I am taking medication that affects blood clotting (aspirin, etc.)

□ Have an allergy to metals.

□ Have a contagious or infectious disease.

□ Currently pregnant or may become pregnant.

□ Never had acupuncture before or have experienced side effects from acupuncture.

□ None of the above. Do not have any symptoms that the physician to be aware of regarding acupuncture.

**Check the health of the patient to watch out for during acupuncture**

□ Drunken alcohol or taken stimulant drugs and being under the influence of them.

□ Currently very angry, surprised, or afraid.

□ Extremely tired or have no energy.

□ Excessive bleeding or blood donation within 24 hours (for women, menstrual bleeding)

□ Sweating profusely due to overexercise or physical weakness.

□ None of the above. Do not have any conditions that require caution with acupuncture.

**Ensure provider hand washing and hygiene**

□ All physicians washed their hands according to the handwashing manual.

□ The physician's nails are clean.

□ The physician checked handwashing and hand hygiene before and after the procedure.

**Check your physician's pre-needling preparation**

□ The physician checks the needle site for wounds or skin disease.

□ Wipe with an alcohol swab in one direction or in a spiral outward motion and wait for it to dry.

□ Sanitize the physician's left hand (the hand that is not holding the needle) with an alcohol swab and palpate the area to be treated.

**Managing the patient's posture before the procedure**

□ Guide the patient into an appropriate and safe position for needling.

□ Guide the patient, taking care not to change position during the procedure.

**5. Practice note forms**

**1) Clean needle technique and needling techniques practice**

| **Practice Classes and Groups:** ** Classes **Groups | | **Practice Topic**: Clean needle technique and needling techniques practice |
| --- | --- | --- |
| **Practice Date**: **March **, 20**. | |  |
| **Practice participants list:** | | |
| **Practice guides** | | |
| - Record the learning contents **(clean needle technique, skin disinfection, needle technique, outline of needle insertion and needle insertion process) in** advance in a practice note through pre-study in groups. - **Every student completes the Safety Checklist for Preparing for Practice and Acupuncture Procedures (in the perspective of patient)** - All students **practice needling with** their own **materials, and after sufficient practice, needle their own bodies.** - Each person performs the entire needling process without assistance from another member of the group and records **their needling practice in a** practice note. - Check your **own hygiene** and **safety practices and** record them in a practice note. - At the end of the practice, **each** student completes the **End of Practice and Acupuncture Procedure Checklist** | | |
| **Pre-Learning Content** | | |
| Clean Needle Technique (CNT) |  | |
| Preparation; Skin Disinfection |  | |
| Preparation; Needling Techniques |  | |
| An overview of needling and needling process |  | |
| **Writer** | **Practice Content** | |
| ***Guidelines for writing practice content: You might want to include the following**   - Materials and supplies used in the practice - What to do about clean needle technique - Needle size (in mm) used for needling practice - Needle direction, angle (perpendicular, oblique, transverse) - Feedback you've received from instructor or other members of your group, etc. | | |
|  |  | |
|  |  | |
|  |  | |
|  |  | |
|  |  | |
| **Writer** | **Self-examination on your learning** | |
| ***A guide to writing a self-examinationion: Self-examination on your learning, objectively evaluate it, and make any necessary improvements. You may want to include the following**   - How was my learning attitude today? - What could be improved (in your own practice performance or learning)? - What questions would you like to ask your instructor? | | |
|  |  | |
|  |  | |
|  |  | |
|  |  | |
|  |  | |

**2) Vital signs measurement practice**

| **Practice Classes and Groups:** ** Classes **Groups | | **Practice Topic**: Vital Signs Measurements Practice |
| --- | --- | --- |
| **Practice Date**: **March **, 20**. | |  |
| **Practice participants list:** | | |
| **Practice guides** | | |
| - Record vital signs measurements in advance in a practice note by working in groups. - Establish physician-patient roles so that every member of the team has one physician and one patient experience with each blood pressure, body temperature, and blood glucose measurement. - All students are required to explain the practice consent form to their patients in person and obtain their consent (practice stops if consent form is not completed) - All students should complete the Safety Checklist for Preparing for Practice and Acupuncture Procedures (completed for their own patient) - Be an observer when you're not acting as a physician or patient, observing the physician's performance, checking for hygiene and safety practices, and providing feedback. - Each physician performs the entire vital signs measurement process without the assistance of another group members, and records the process, vital signs measurement results, and feedback received in a practice note. - At the end of the practice, each student completes the Post-procedure Checklist | | |
| **Pre-Learning Content** | | |
| How to measure blood pressure |  | |
| How to measure body temperature |  | |
| How to measure blood glucose |  | |
| **Writer** | **Practice Description** | |
| ***Guidelines for writing practice descriptions: You might want to include the following**   - Materials and supplies used in the practice - As a physician, what vital signs were measured (patients’ name), how they were measured, and the results - Experiencing vital signs measurement as a patient, what it feels like - Feedback you've received from instructors or other members of your group, etc. | | |
|  |  | |
|  |  | |
|  |  | |
|  |  | |
|  |  | |
| **Writer** | **Self-examination on your learning** | |
| ***A guide to writing a self-examinationion: Self-examination on your learning, objectively evaluate it, and make any necessary improvements. You may want to include the following**   - How was my learning attitude today? - What could be improved (in your own practice performance or learning)? - What questions would you like to ask your instructor? | | |
|  |  | |
|  |  | |
|  |  | |
|  |  | |
|  |  | |

**3) Locating acupuncture points and needling**

| **Practice Classes and Groups:** ** Classes **Groups | | | **Practice Topic**: |
| --- | --- | --- | --- |
| **Practice Date**: **March **, 20**. | | |  |
| **Practice participants list:** | | | |
| **Practice guides** | | | |
| - Record locating procedure of each acupuncture points in a practice note by working in groups. - Assign roles for the practice day ahead of time as physician, patient, and observer. - Explain the consent form directly to the patient and obtain their consent (stop the practice if the consent form is not completed) - The physician checks the health of the patient and measures vital signs. - The observer complete a pre-procedure checklist. - Each physician performs the entire needling process without the assistance of another group members and records their performance in a practice note. - The observer monitors the physician's performance, check for hygiene and safety practices, and record them in the practice note. - At the end of the practice, the observer will have each physician perform one acupuncture needle insertion on the acupuncture points provided by the instructor, and grade and give feedback according to the checklist (OSCE). | | | |
| **Observer name:** | | **Sanitation checks** | \| **Checklist** \| 2 \| 1 \| 0 \| \| --- \| --- \| --- \| --- \| \| Are your nails filed before practice? \|  \|  \|  \| \| Did you wash your hands at the sink after entering the lab? \|  \|  \|  \| \| Did you wash your hands immediately before the acupuncture procedure? \|  \|  \|  \| \| Was the needling site disinfected before the procedure? \|  \|  \|  \| \| Was the needling site disinfected after the procedure? \|  \|  \|  \| \| Did you use a bedcover during the practice? \|  \|  \|  \| \| Did you properly dispose of used supplies after the procedure? \|  \|  \|  \|   2 points: Did. / 1 point: I did it incompletely / 0 points: I didn't do it.  **Check results & improvement points:** |
|  |  | **Safety Management Summary** | \| **Checklist** \| 2 \| 1 \| 0 \| \| --- \| --- \| --- \| --- \| \| Did the physician explain the procedure to the patient and obtain consent? \|  \|  \|  \| \| Have you checked the vital signs of the patient? \|  \|  \|  \| \| Did you complete the pre-procedure checklist? \|  \|  \|  \| \| Did you guide the patient to the proper acupuncture point locating posture? \|  \|  \|  \| \| Did you consider the anatomical structure around the needling site when performing the locating acupuncture points and needling procedure? \|  \|  \|  \| \| Is the needle inserted at the proper depth? \|  \|  \|  \| \| Was the physician considerate of the patient during the procedure? \|  \|  \|  \| \| If bleeding occured, have efforts been made to hemostasis and prevent hematoma? \|  \|  \|  \| \| Did you follow the practice dialog guidelines? \|  \|  \|  \| \| Did you complete the post-procedure checklist? \|  \|  \|  \|   2 points: Did. / 1 point: I did it incompletely / 0 points: I didn't do it.  **Check results & improvement points:** |
|  |  | **Observations and feedback about your assistant's performance** |  |
| **Physician name:** | | **Proper acupuncture point locating and posture** |  |
|  |  | **Physician-specific performance, communication, and attitude** |  |
|  |  | **What I learned from the practice** |  |
| **Physician name:** | | **Patient vital sign** | - Body temperature: ℃. - Blood pressure: / mmHg - Blood glucose: mg/dL (time since meal: hours) |
|  |  | **Specific symptoms/conditions of the patient** |  |
| **Needle size used (in mm)** | | **Record by site (acupoint)** |  |
| **Practice Content** | | | |
| ***Guide to writing practice content**   \| **Item** \| **Essential** \| **Optional** \| \| --- \| --- \| --- \| \| **Prior learning** \| - Locating acupuncture points \| - Site and surrounding anatomical structure - Precautions for needling \| \| **Physician by acupoint** \| - Locating acupuncture points process (be specific) - Needle direction, angle (perpendicular/oblique/transverse) - Needle depth (in mm) - Targeted anatomical structures when needling - Adverse events (if they occur during the procedure) and treatment \| - Patient's reaction - How did you feel during the procedure (e.g., needle sensation)? - Photographs of needling (if adverse events occur due to the procedure, photographs and documentation are recommended as evidence in case of treatment) \| \| **OSCE Scoring Results** \| Attach an image captured from a Google Form (if you did an OSCE) \| \| | | | |
| **LU1** | **Prior learning** |  | |
|  | **Physician name:** |  | |
|  | **Physician name:** |  | |
| **LU2** | **Prior learning** |  | |
|  | **Physician name:** |  | |
|  |  |  | |
| **Self-examination on your learning** | | | |
| ***A guide to writing a self-examinationion: Self-examination on your learning, objectively evaluate it, and make any necessary improvements. You may want to include the following**   - How was my learning attitude today? - What could be improved (in your own practice performance or learning)? - What questions would you like to ask your instructor? | | | |
| **Name:** | |  | |
| **Name:** | |  | |
| **Name:** | |  | |
| **Name:** | |  | |
| **Name:** | |  | |
| **Observer OSCE Scoring Results** | | | |
| **Physician name:** | |  | |
| **Physician name:** | |  | |
| **Physician name:** | |  | |

**4) Acupuncture technique**

| **Practice Classes and Groups:** ** Classes **Groups | | | | **Practice Topic**: |
| --- | --- | --- | --- | --- |
| **Practice Date**: **March **, 20**. | | | |  |
| **Practice participants list:** | | | | |
| **Practice guides** | | | | |
| - Record locating procedure of each acupuncture points in a practice note by working in groups. - Assign roles for the practice day ahead of time as physician, patient, and observer. - Explain the consent form directly to the patient and obtain their consent (stop the practice if the consent form is not completed) - The physician checks the health of the patient and measures vital signs. - The observer completes a pre-procedure checklist. - Each physician performs the entire needling process without the assistance of another group members and records their performance in a practice log. - The observer monitors the physician's performance, check for hygiene and safety practices, and record them in the practice note. - At the end of the practice, the observer will have each physician perform one acupuncture needle insertion on the acupuncture points provided by the instructor, and grade and give feedback according to the checklist (OSCE). | | | | |
| **Prior learning** | | | | |
| **Technique procedure** | | |  | |
| **Indications** | | |  | |
| **Precaution** | | |  | |
| **Practice** | | | | |
| **Observer name:** | | **Sanitation checks** | | \| **Checklist** \| 2 \| 1 \| 0 \| \| --- \| --- \| --- \| --- \| \| Are your nails filed before practice? \|  \|  \|  \| \| Did you wash your hands at the sink after entering the lab? \|  \|  \|  \| \| Did you wash your hands immediately before the acupuncture procedure? \|  \|  \|  \| \| Was the needling site disinfected before the procedure? \|  \|  \|  \| \| Was the needling site disinfected after the procedure? \|  \|  \|  \| \| Did you use a bedcover during the practice? \|  \|  \|  \|   2 points: Did. / 1 point: I did it incompletely / 0 points: I didn't do it.  **Check results & improvement points:** |
|  |  | **Safety Management Summary** | | \| **Checklist** \| 2 \| 1 \| 0 \| \| --- \| --- \| --- \| --- \| \| Did the physician explain the procedure to the patient and obtain consent? \|  \|  \|  \| \| Have you checked the vital signs of the patient? \|  \|  \|  \| \| Did you complete the pre-procedure checklist? \|  \|  \|  \| \| Did you guide the patient to the proper acupuncture point locating posture? \|  \|  \|  \| \| Did you consider the anatomical structure around the needling site when performing the locating acupuncture points and needling procedure? \|  \|  \|  \| \| Is the needle inserted at the proper depth? \|  \|  \|  \| \| Was the physician considerate of the patient during the procedure? \|  \|  \|  \| \| If bleeding occurs, have efforts been made to hemostasis and prevent hematoma? \|  \|  \|  \| \| Did you follow the practice dialog guidelines? \|  \|  \|  \|   2 points: Did. / 1 point: I did it incompletely / 0 points: I didn't do it.  **Check results & improvement points:** |
|  |  | **Observations and feedback about your group members’ performance** | |  |
| **Physician name:** | | **Proper acupuncture point locating and posture** | |  |
|  |  | **Physician-specific performance, communication, and attitude** | |  |
|  |  | **What I learned from the practice** | |  |
| **Physician name:** | | **Patient vital sign** | | - Body temperature: ℃. - Blood pressure: / mmHg - Blood glucose: mg/dL (time since meal: hours) |
|  |  | **Specific symptoms/conditions of the patient** | |  |
| **Procedure type** | **Practice Content** | | | |
| ***Guide to writing practice content**   \| **Item** \| **Required** \| **Create selections** \| \| --- \| --- \| --- \| \| **Physician by acupoint** \| - Procedure site, locating acupuncture points process (be specific) - Used supplies, procedure process, procedure time - Adverse events (if they occur during the procedure) and treatment \| - Patient's reaction - How did you feel during the procedure (e.g., needle sensation)? - Photographs of needling (if adverse events occur due to the procedure, photographs and documentation are recommended as evidence in case of treatment) \| \| **OSCE Scoring Results** \| Attach an image captured from a Google Form (if you did an OSCE) \| \| | | | | |
| **Physician name:** | |  | | |
| **Physician name:** | |  | | |
| **Self-examination on your learning** | | | | |
| ***A guide to writing a self-examinationion: Self-examination on your learning, objectively evaluate it, and make any necessary improvements. You may want to include the following**   - How was my learning attitude today? - What could be improved (in your own practice performance or learning)? - What questions would you like to ask your instructor? | | | | |
| **Name:** | |  | | |
| **Name:** | |  | | |
| **Name:** | |  | | |
| **Name:** | |  | | |
| **Name:** | |  | | |
| **Observer OSCE Scoring Results** | | | | |
| **Physician name:** | |  | | |
| **Physician name:** | |  | | |
| **Physician name:** | |  | | |

**6. Rubric for grading practice note**

|  | Great  (5) | Good  (4) | Average  (3) | Insufficient  (2) | Very poor  (1) |
| --- | --- | --- | --- | --- | --- |
| Did students check the patient's vital signs and physical abnormalities? |  |  |  |  |  |
| Did the locating acupuncture points and the procedure appropriate? |  |  |  |  |  |
| Was hygiene and safety checked throughout the practice? |  |  |  |  |  |
| Did students properly utilize the necessary supplies and devices during the practice? |  |  |  |  |  |
| Did students specifically record what they did in the practice? |  |  |  |  |  |
| Was each practice participant's role and activity appropriate? |  |  |  |  |  |
