# Supplementary material for: Implementing a competency-based acupuncture training program in Korean Medicine education
Source: PLoS One. 2026 Mar 20;21(3):e0345289. doi: 10.1371/journal.pone.0345289 (PMC13004403; doi:10.1371/journal.pone.0345289)
Supplement: S2 File — (DOCX) [file pone.0345289.s002.docx]

**1. Survey questionnaire**

1) Korean version of Self-efficacy Questionnaire

| **No.** | **Items of KSE-12** | **Response** |
| --- | --- | --- |
| 1 | How certain are you that you are able to successfully identify the issues the patient wishes to address during the conversation? | \| Very uncertain \| \| \| \| \| Very certain \| \| \| \| \| \| --- \| --- \| --- \| --- \| --- \| --- \| --- \| --- \| --- \| --- \| \| 1 \| 2 \| 3 \| 4 \| 5 \| 6 \| 7 \| 8 \| 9 \| 10 \| |
| 2 | How certain are you that you are able to successfully make an agenda/plan for the conversation with the patient? | \| Very uncertain \| \| \| \| \| Very certain \| \| \| \| \| \| --- \| --- \| --- \| --- \| --- \| --- \| --- \| --- \| --- \| --- \| \| 1 \| 2 \| 3 \| 4 \| 5 \| 6 \| 7 \| 8 \| 9 \| 10 \| |
| 3 | How certain are you that you are able to successfully urge the patient to expand on his or her problems/worries? | \| Very uncertain \| \| \| \| \| Very certain \| \| \| \| \| \| --- \| --- \| --- \| --- \| --- \| --- \| --- \| --- \| --- \| --- \| \| 1 \| 2 \| 3 \| 4 \| 5 \| 6 \| 7 \| 8 \| 9 \| 10 \| |
| 4 | How certain are you that you are able to successfully listen attentively to the patient? | \| Very uncertain \| \| \| \| \| Very certain \| \| \| \| \| \| --- \| --- \| --- \| --- \| --- \| --- \| --- \| --- \| --- \| --- \| \| 1 \| 2 \| 3 \| 4 \| 5 \| 6 \| 7 \| 8 \| 9 \| 10 \| |
| 5 | How certain are you that you are able to successfully encourage the patient to express thoughts and feelings? | \| Very uncertain \| \| \| \| \| Very certain \| \| \| \| \| \| --- \| --- \| --- \| --- \| --- \| --- \| --- \| --- \| --- \| --- \| \| 1 \| 2 \| 3 \| 4 \| 5 \| 6 \| 7 \| 8 \| 9 \| 10 \| |
| 6 | How certain are you that you are able to successfully structure the conversation with the patient? | \| Very uncertain \| \| \| \| \| Very certain \| \| \| \| \| \| --- \| --- \| --- \| --- \| --- \| --- \| --- \| --- \| --- \| --- \| \| 1 \| 2 \| 3 \| 4 \| 5 \| 6 \| 7 \| 8 \| 9 \| 10 \| |
| 7 | How certain are you that you are able to successfully demonstrate appropriate nonverbal behaviors (eye contact, facial expression, placement, posture, and voicing)? | \| Very uncertain \| \| \| \| \| Very certain \| \| \| \| \| \| --- \| --- \| --- \| --- \| --- \| --- \| --- \| --- \| --- \| --- \| \| 1 \| 2 \| 3 \| 4 \| 5 \| 6 \| 7 \| 8 \| 9 \| 10 \| |
| 8 | How certain are you that you are able to successfully show empathy (acknowledge the patient’s views and feelings)? | \| Very uncertain \| \| \| \| \| Very certain \| \| \| \| \| \| --- \| --- \| --- \| --- \| --- \| --- \| --- \| --- \| --- \| --- \| \| 1 \| 2 \| 3 \| 4 \| 5 \| 6 \| 7 \| 8 \| 9 \| 10 \| |
| 9 | How certain are you that you are able to successfully clarify what the patient knows in order to communicate the right amount of information? | \| Very uncertain \| \| \| \| \| Very certain \| \| \| \| \| \| --- \| --- \| --- \| --- \| --- \| --- \| --- \| --- \| --- \| --- \| \| 1 \| 2 \| 3 \| 4 \| 5 \| 6 \| 7 \| 8 \| 9 \| 10 \| |
| 10 | How certain are you that you are able to successfully check patient’s understanding of the information given? | \| Very uncertain \| \| \| \| \| Very certain \| \| \| \| \| \| --- \| --- \| --- \| --- \| --- \| --- \| --- \| --- \| --- \| --- \| \| 1 \| 2 \| 3 \| 4 \| 5 \| 6 \| 7 \| 8 \| 9 \| 10 \| |
| 11 | How certain are you that you are able to successfully make a plan based on shared decisions between you and the patient? | \| Very uncertain \| \| \| \| \| Very certain \| \| \| \| \| \| --- \| --- \| --- \| --- \| --- \| --- \| --- \| --- \| --- \| --- \| \| 1 \| 2 \| 3 \| 4 \| 5 \| 6 \| 7 \| 8 \| 9 \| 10 \| |
| 12 | How certain are you that you are able to successfully close the conversation by assuring, that the patient’s questions have been answered? | \| Very uncertain \| \| \| \| \| Very certain \| \| \| \| \| \| --- \| --- \| --- \| --- \| --- \| --- \| --- \| --- \| --- \| --- \| \| 1 \| 2 \| 3 \| 4 \| 5 \| 6 \| 7 \| 8 \| 9 \| 10 \| |

2) Survey on the acupuncture training program

| **No.** | **Survey questions** | **Response** |
| --- | --- | --- |
| **Part 1. Reaction evaluation** | | |
| 1 | Was the class interesting? | \| Strongly agree \| Disagree \| Neutral \| Agree \| Strongly agree \| \| --- \| --- \| --- \| --- \| --- \| |
| 2 | Was the level of training appropriate? | \| Strongly agree \| Disagree \| Neutral \| Agree \| Strongly agree \| \| --- \| --- \| --- \| --- \| --- \| |
| 3 | Was the learning material directly related to the learning objectives? | \| Strongly agree \| Disagree \| Neutral \| Agree \| Strongly agree \| \| --- \| --- \| --- \| --- \| --- \| |
| 4 | Was the learning material easy to understand? | \| Strongly agree \| Disagree \| Neutral \| Agree \| Strongly agree \| \| --- \| --- \| --- \| --- \| --- \| |
| 5 | Was the learning material useful? | \| Strongly agree \| Disagree \| Neutral \| Agree \| Strongly agree \| \| --- \| --- \| --- \| --- \| --- \| |
| 6 | Was the content of learning material clear? | \| Strongly agree \| Disagree \| Neutral \| Agree \| Strongly agree \| \| --- \| --- \| --- \| --- \| --- \| |
| 7 | Was the practice opportunity sufficient? | \| Strongly agree \| Disagree \| Neutral \| Agree \| Strongly agree \| \| --- \| --- \| --- \| --- \| --- \| |
| 8 | Do you think the peer OSCE activity in the class is appropriate? | \| Strongly agree \| Disagree \| Neutral \| Agree \| Strongly agree \| \| --- \| --- \| --- \| --- \| --- \| |
| 9 | How satisfied are you with the training program overall? | \| Strongly agree \| Disagree \| Neutral \| Agree \| Strongly agree \| \| --- \| --- \| --- \| --- \| --- \| |
| 9-1 | Why do you think so? | (open-ended) |
| 10 | Was the training method effective? | \| Strongly agree \| Disagree \| Neutral \| Agree \| Strongly agree \| \| --- \| --- \| --- \| --- \| --- \| |
| 10-1 | Why do you think so? | (open-ended) |
| 11 | Was the purpose and content of the training aligned in your opinion? | \| Strongly agree \| Disagree \| Neutral \| Agree \| Strongly agree \| \| --- \| --- \| --- \| --- \| --- \| |
| 11-1 | Why do you think so? | (open-ended) |
| 12 | Was the amount of time given for this class during this semester adequate? | \| Strongly agree \| Disagree \| Neutral \| Agree \| Strongly agree \| \| --- \| --- \| --- \| --- \| --- \| |
| 13 | Was the time allotted for preparation, presentation of learning objectives, and meditation (introduction session, 20 minutes) sufficient? | \| Strongly agree \| Disagree \| Neutral \| Agree \| Strongly agree \| \| --- \| --- \| --- \| --- \| --- \| |
| 14 | Was the time allotted for the teacher’s presentation (demonstration) (development session, 30 minutes) sufficient? | \| Strongly agree \| Disagree \| Neutral \| Agree \| Strongly agree \| \| --- \| --- \| --- \| --- \| --- \| |
| 15 | Was the time allotted for the group exercises and wrap-up (development and wrap-up session, 100 minutes) sufficient? | \| Strongly agree \| Disagree \| Neutral \| Agree \| Strongly agree \| \| --- \| --- \| --- \| --- \| --- \| |
| **Part 2. Suitability of flipped learning course operation** | | |
| 16 | Was the average class time for online prior learning appropriate? | \| Strongly agree \| Disagree \| Neutral \| Agree \| Strongly agree \| \| --- \| --- \| --- \| --- \| --- \| |
| 17 | Are learning videos attractive to promote learning? | \| Strongly agree \| Disagree \| Neutral \| Agree \| Strongly agree \| \| --- \| --- \| --- \| --- \| --- \| |
| 18 | Did you watch the learning videos for review? | \| Strongly agree \| Disagree \| Neutral \| Agree \| Strongly agree \| \| --- \| --- \| --- \| --- \| --- \| |
| 19 | Does prior online learning increase your understanding of classes? | \| Strongly agree \| Disagree \| Neutral \| Agree \| Strongly agree \| \| --- \| --- \| --- \| --- \| --- \| |
| 20 | Was it convenient to access the online learning management system? | \| Strongly agree \| Disagree \| Neutral \| Agree \| Strongly agree \| \| --- \| --- \| --- \| --- \| --- \| |
| 21 | Were you able to study freely at your desired time and place? | \| Strongly agree \| Disagree \| Neutral \| Agree \| Strongly agree \| \| --- \| --- \| --- \| --- \| --- \| |
| 22 | What could be improved in online learning? | (open-ended) |
| 23 | Did learning activities make learning more interesting? | \| Strongly agree \| Disagree \| Neutral \| Agree \| Strongly agree \| \| --- \| --- \| --- \| --- \| --- \| |
| 24 | Were the learning activities appropriate to achieve the learning objectives? | \| Strongly agree \| Disagree \| Neutral \| Agree \| Strongly agree \| \| --- \| --- \| --- \| --- \| --- \| |
| 25 | Was communication between the instructor and the student communication good? | \| Strongly agree \| Disagree \| Neutral \| Agree \| Strongly agree \| \| --- \| --- \| --- \| --- \| --- \| |
| 26 | Was communication between the students good? | \| Strongly agree \| Disagree \| Neutral \| Agree \| Strongly agree \| \| --- \| --- \| --- \| --- \| --- \| |
| 27 | Was the learning activity appropriately assessed? | \| Strongly agree \| Disagree \| Neutral \| Agree \| Strongly agree \| \| --- \| --- \| --- \| --- \| --- \| |
| 28 | Was the content alignment between pre-learning and classroom learning appropriate? | \| Strongly agree \| Disagree \| Neutral \| Agree \| Strongly agree \| \| --- \| --- \| --- \| --- \| --- \| |
| 29 | What could be improved in offline learning? | (open-ended) |

**2. Script of semi-structured interview**

**Introduction**

Thank you for taking your time to participate in this study. This interview will take about 1 hour. I will ask you some questions about the acupuncture training program. There are no right answers. Your personal information will kept confidential. The interview will be audio recorded, and you may request to stop the recording during the interview. Do you have any questions before we begin?

**Main questionnaire**

- How was the acupuncture training experience?
- How did you use the learning manual before / during / after class?
- What are the strengths of this course?
- What are the weaknesses and improvements of this course?
