# Supplementary material for: Implementing a competency-based acupuncture training program in Korean Medicine education
Source: PLoS One. 2026 Mar 20;21(3):e0345289. doi: 10.1371/journal.pone.0345289 (PMC13004403; doi:10.1371/journal.pone.0345289)
Supplement: S5 File — (DOCX) [file pone.0345289.s005.docx]

**Interview with Student 1**

Interviewer

Well, you arrived quickly. So, did you know there's a learning manual for the acupuncture training program?

Student 1

Yes, I did.

Interviewer

How did you first find out about the manual?

Student 1

Our professor presented it to students during the orientation session.

Interviewer

Then, do you know what's in the manual?

Student 1

I can’t remember exactly, but I remember the gist of it.

Interviewer

Could you tell me what you know?

Student 1

I remember about hygiene and things like that—how you should set up the environment properly when doing procedures.

Interviewer

Is there anything else you remember?

Student 1

I'm afraid I don't remember the rest.

Interviewer

Well then, do you remember if there was a table that outlined what students should do in class?

Student 1

Yes, I remember that.

Interviewer

Then, could you tell me specifically how you used the manual?

Student 1

From the beginning, I followed the manual for all stages: prep before the lesson, during the lesson, and cleanup after. The manual consistently emphasized hygiene and patient safety, so following it helped reduce confusion and made the whole process run smoothly and efficiently.

Interviewer

Do you mean that you followed the manual throughout the entire training process?

Student 1

Yes, I did.

Interviewer

Were there any parts you found confusing or difficult when using the manual?

Student 1

Nothing was really that hard. I just got the hang of it as I kept using it.

Interviewer

Then, in addition to that table with the procedures, there were also examples of practice notes, hand-washing instructions, and equipment manuals. Do you know about those, or have you used them?

Student 1

Yes, that was my first time experiencing what the professor introduced. As I filmed training classes and kept practicing throughout the 16 weeks, it became almost like a habit.

Interviewer

What do you mean specifically?

Student 1

Starting from handwashing, placing the sanitary pad, obtaining the participant’s consent, and then proceeding and finishing the practice — everything was done following the manual, almost automatically.

Interviewer

Well then, looking back at the beginning of the acupuncture training class, did you ever find it confusing or difficult to know what to do during the class?

Student 1

In the very beginning, when we first started the practice sessions, the professor couldn't supervise everyone at once, so just the prep alone took over an hour. I remember it being really chaotic—things like separating waste during cleanup—because students had no idea what they were supposed to do. But as we kept practicing, we got so used to it that prep time eventually shortened to under 10 minutes.

Interviewer

So, what did you find most difficult during the early stage of the practice?

Student 1

The most difficult part was checking the participant’s vital signs.

Interviewer

Was there anything else you found difficult?

Student 1

Not really. I was just a bit confused about disposing of the medical waste.

Interviewer

Have you ever seen the procedure for disposing of medical waste in the manual?

Student 1

I’m not sure if this is exactly the manual, but I used the PDF file provided by the professor a lot.

Interviewer

Was it there?

Student 1

Yes, it was.

Interviewer

What did you enjoy about the practical training?

Student 1

At first, when we practiced on our own, we often did things without following any formal procedures. The professor would sometimes give instructions, almost like mandatory steps, but since the training was designed to be standardized and stable, it really helped us develop a routine. I found that helpful.

Interviewer

What exactly do you mean by ‘developing a routine’?

Student 1

I realized that if I do things the same way as in the training, I can treat a patient in a more stable setting. So, you know, I really got a clear understanding of hygienic needling techniques, like CNT.

Interviewer

You talked a bit about what’s needed for the procedure. Could you go into a little more detail?

Student 1

So basically, the most important thing is to make sure the environment doesn’t interfere with the patient’s hygiene. Even when using a bed, you have to cover it completely with a sanitary cover. And the instruments we use need to be properly disinfected in an infrared or UV sterilizer. Plus, after cleaning the treatment area with an alcohol swab, you have to disinfect your hands and the patient’s site both before and after the area.

Interviewer

So, you’re saying you learned these steps as the necessary procedures for the treatment?

Student 1

Yes.

Interviewer

Was there anything else you liked about the practical training?

Student 1

Well, that’s all.

Interviewer

So, over the semester, what did you find difficult during the acupuncture training?

Student 1

I didn’t think it was too difficult overall, but one thing I did find challenging was when we had to locate an acupuncture point by palpating anatomical structures. I couldn’t really tell whether I was palpating was the right structure or not, so I ended up asking the teaching assistants for help quite a lot. It’s not that getting help from them was hard — it’s just that part was tricky, so I had to rely on them a lot.

Interviewer

Then, could you give a specific example of that? For instance, was there any particular structure that you found especially difficult to palpate?

Student 1

When I had to locate ST36, I had to feel for the nerve under the tibialis anterior muscle, but it was hard to tell if what I was pressing was a blood vessel, a nerve, or a muscle — or if I was even stimulating it properly. I also got a bit confused when palpating the intercostal spaces.

Interviewer

So, what did you do when you faced those kinds of difficulties?

Student 1

For the intercostal spaces, I tried to find them as accurately as I could by using the xiphoid process and the xiphisternal joint as reference points. And when it came to stimulating structures like the tibialis anterior muscle, I got help from the professor.

Interviewer

So, did you get help from the professor? Or was it easy for you to ask for help in that environment?

Student 1

Actually, not only the professor but also the teaching assistants joined the class. They went around and answered our questions, so it felt a lot easier and more comfortable to go up to the professor and ask for help.

Interviewer

So, thinking about the whole practical training — like before class when you had to divide up roles, get things ready according to the manual, start the lesson, watch the professor’s demonstration, then do the practice yourself with acupuncture point location and needling, and finally the OSCE at the end — was there anything you found difficult during that whole process?”

Student 1

The professor’s demonstration tended to be a bit lengthy.

Interviewer

Really? How long did it usually go on for?

Student 1

I think it lasted around an hour to an hour and twenty minutes.

Interviewer

Was it that long every time?

Student 1

Yes. So, OSCE and the sessions where we practiced with each other weren’t too hard, since we were learning through hands-on experience. But the professor’s demonstrations tended to be a bit long, so I sometimes found it hard to stay fully focused.

Interviewer

What kind of things did you mainly learn during the professor’s demonstrations?

Student 1

About half of the time, the professor showed needling practice directly on acupuncture points, and the other half focused on showing anatomical structures using ultrasound.

Interviewer

When you watched the needling part, did you feel that you could understand it well?

Student 1

I could see examples of the depth, direction, and type of needles used, but it was difficult to actually feel what those sensations were like. So when I tried to perform the needling by myself later, I realized that there were many points where it was hard to reproduce the same technique just by watching.

Interviewer

So, you mean there were many points where it was hard to insert the needle in the same way as the professor did? Can you remember any specific acupuncture points?

Student 1

ST36 was little hard.

Interviewer

Okay. Please, go on.

Student 1

For ST36, the professor inserted the needles in two different directions, and I couldn’t really get a sense of how exactly they were targeting the structures or why they used those angles. There were also very sensitive points around the chest area. The professor would easily locate points like CV22—I think that’s the name?—and insert the needle from the neck to the chest, but when we tried it ourselves, it was hard to get the needle in the same way.

Interviewer

So, are you saying that even through the demonstration, you felt there were some limitations in learning the technique?

Student 1

Yes, through the demonstration.

Interviewer

So, you mean that it wasn’t very easy to follow along when watching the professor’s demonstration?

Student 1

Yes. I knew the method, but when I tried it myself, I got pretty confused about the directions and the structures, and I spent a lot of time thinking about how exactly to insert the needle. I kept wondering, ‘Am I needling it in the same direction as the professor did?’ a lot.

Interviewer

So, do you think there could be any other ways to make it a bit less confusing?

Student 1

Well, I think the best way would be to go around group by group and have someone demonstrate the needling right in front of a student, so the student could really feel it. But given the limited class time, I also realized that might not be possible.

Interviewer

Do you think it would help you understand better if it were demonstrated right in front of you?

Student 1

Yes. Actually, when I asked the professor and he demonstrated it right in front of me, I understood it much better.

Interviewer

It was really helpful when the professor come to that group and demonstrate there.

And you also mentioned the ultrasound — when the professor demonstrated, were you able to understand the positions of the structures shown on the ultrasound?

Student 1

Oh, it was my first time seeing ultrasound in this class, so I didn’t fully understand everything. But I could learn how to locate the structures and how to tell which is muscle and which is a blood vessel based on their movement.

Interviewer

So you got a sense of how to distinguish between muscles and blood vessels, and since the ultrasound shows structures in three dimensions, were you also able to understand how the probe was placed and how the structures appear on the screen?

Student 1

We were so focused on the images that even though the professor was explaining the probe carefully, we couldn’t really match it with what we were seeing on the screen. The explanation was given, but from our perspective, we couldn’t observe both screens at once — the monitors were a bit far apart. One monitor showed the image, and the other showed the probe view, so it was kind of hard to follow along properly.

Interviewer

Earlier, you mentioned that it helps to watch the professor demonstrate acupuncture points right in front of you. Did you also have the experience of the professor demonstrating ultrasound when you came to your group?

Student 1

I remember he did a demonstration once.

Interviewer

And when the professor showed it directly in your group, did you understand it better?

Student 1

Yes, I understood it that time.

Interviewer

So, did you watch the pre-class videos before the class?

Student 1

Yes, I watched all of them.

Interviewer

And how did you find those videos? Did you understand them well?

Student 1

Yes. In those videos, there was one where they showed a 3D image over the patient’s body to visually demonstrate the anatomical structures. I remember that being the easiest to understand.

Interviewer

Which area was that? The one where the professor visually showed the anatomical structures?

Student 1

It was the thigh. I think I understood the upper limb and lower limb really well, especially in the smaller, more detailed muscle regions.

Interviewer

Do you mean that you understood the smaller areas better?

Student 1

Yes. So, I understood the thigh, of course, but when the leg and distal areas were shown in 3D like that, it was even more helpful.

Interviewer

And was there anything else from the pre-class videos that you found helpful?

Student 1

I paid close attention to the cautions they mentioned about areas to be careful when needling.

Interviewer

Do you remember any of those cautions specifically?

Student 1

The one I remember most clearly was about the neck and shoulder areas, where there are a lot of nerves and the stimulation could be strong enough to cause fainting. So, during practice, we tried to avoid needling those points and used pressure instead whenever possible.

Interviewer

Ah, then what were the differences between the pre-class videos and the professor’s demonstrations during class?

Student 1

Well, during the demonstrations, sometimes the professor would add extra explanations if he wanted to expand on a point, so the content could get a bit scattered.
But the pre-class videos focused only on the essential content, explaining the acupuncture points and needling in a very concise and structured way, so they were easier to follow.

Interviewer

Were the pre-class videos newly recorded, or were they older demonstration recordings?

Student 1

I think they were from a few years ago.

Interviewer

Were those videos recorded with students participating?

Student 1

Yes.

Interviewer

But the content was more concise and structured.

Student 1

Yes. Since it was edited, a lot of the extra content was cut out.

Interviewer

Ah, so it was edited. Did the pre-class videos include ultrasound as well?

Student 1

No, not really. There were a few instances, but very rarely, and most of the videos just showed the needling scenes.

Interviewer

So, through the pre-class learning, were you able to get a better sense of the depth and direction of the needles compared to the live demonstration?

Student 1

I think it was similar. In the videos, there were more verbal explanations, so we could somewhat quantify it. For example, instead of saying it should be shallow, they would specify something like, ‘You can insert 20mm here, 30mm there.’ We would note that down during the pre-class learning and then try it out during the actual class.

Interviewer

Were the needling depths mentioned a lot in the pre-class videos?

Student 1

Yes, they were, so we noted that information and used it to study before class.

Interviewer

Since we’re talking about preparing for the acupuncture training class, what kind of things did you study before the class? Did you study or prepare anything on your own?

Student 1

I focused most on the anatomical structures. Besides that, I studied how to locate acupuncture points by bone standard and also studied the cautions or contraindications for needling those points.

Interviewer

You mentioned anatomical structures — how did you study those?

Student 1

Well, I looked up the structures mentioned in our textbooks and handbooks online, to learn how to palpate them or make them more visible.
I also studied how to apply resistance exercises to make the muscles stand out. Basically, I studied this information in a distributed way from multiple sources.

Interviewer

So, you searched that information online, not in the textbooks or handbook?

Student 1

Yes. I browsed online, and I also used the anatomy PPT from our second-year premedical courses to find information.

Interviewer

Was the second-year anatomy PPT helpful?

Student 1

Yes, it was actually quite helpful.

Interviewer

Then, for learning how to find the bone proportional points, what resources did you refer to?

Student 1

When the professor mentioned it during demonstrations, I noted that, and if it wasn’t mentioned, I often referred to the professor’s PPT.

Interviewer

You mean both the PPT and the pre-class videos?

Student 1

Yes. I watched the pre-class videos first, and for anything not mentioned there, I referred to the professor’s PPT next.

Interviewer

Then, what resources did you use for the cautions and contraindications?

Student 1

I mainly studied them that the professor mentioned.

Interviewer

From the pre-class videos?

Student 1

Yes.

Interviewer

So with that preparation, when you came to the acupuncture training class, how much did it help you with locating acupuncture points and needling?

Student 1

I wouldn’t say I did it perfectly, but I could find about 80% of the points on my own.

Interviewer

You mentioned about 80% — were there any areas where you felt it would have been helpful to have more pre-class learning. Maybe something more in advance, or a demonstration, or even just tips on how to palpate the muscles better?

Student 1

Yes, some guidance on how to feel the muscles more effectively would have made things easier.

Interviewer

And I’d like to ask a bit about the OSCE. Had you experienced OSCEs before?

Student 1

OSCE? No, I hadn’t done it outside of the class.

Interviewer

So you hadn’t done it before the acupuncture training class. How did it feel when you were told you would do an OSCE?

Student 1

At first, I didn’t even know what the term OSCE meant. But then I realized it was basically a self-test between students, with someone acting as the examiner and the other as the one being tested.

So I thought of it as practice we could do among ourselves, without worrying about the big fancy term. It felt like a light practice — somewhere between rehearsal and the real thing.

Interviewer

So you’ve both done the evaluations yourself and been evaluated?

Student 1

Yes, that’s right.

Interviewer

How did it feel when you were being evaluated?

Student 1

There were a lot of things I didn’t realize, and I was able to get feedback on a lot of things from a peer’s perspective, which helped me fill in the gaps.

Interviewer

Do you remember any specific feedback you received?

The audio’s muted right now, so just checking.

Student 1

I didn’t get much feedback on locating points itself, just that I pressed a little too hard during the palpation process.

Interviewer

So, when you received feedback, was it only based on the checklist?

Student 1

Yes, that’s right.

Interviewer

And how was it when you were the evaluator?

Student 1

When I was observing a friend, I felt there were a lot more things I needed to pay attention to.
At the same time, watching my friend also gave me a chance to reflect on what I had been missing myself.

Interviewer

Do you remember anything specific that made you reflect on your own practice?

Student 1

The biggest thing I noticed was during palpation. I should have palpated the patient’s area, then disinfected my hands and the area again before needling. But I went straight from palpation to needling, and I realized that could be seen as a hygiene issue.

Interviewer

Anything else?

Student 1

Nothing else comes to mind right now

Interviewer

You mentioned there was a lot to observe — do you mean as an evaluator, there were many things you had to watch for?

Student 1

Yes, a bit. Hygiene, of course, but also whether palpation was done correctly during the procedure, whether hygiene was maintained throughout, whether the patient interaction was good, if the patient’s condition was being monitored properly, whether the posture during needling was correct, and after finishing, whether disinfection was done properly and bleeding was checked.
I had to observe all of that overall, so checking both hygiene and the procedure at the same time was a bit challenging.

Interviewer

“I see. So how many times have you experienced the OSCE?

Student 1

I evaluated others two or three times, and I was evaluated about once, I think.

Interviewer

So in total, how many times did your group go through it?

Student 1

Around five or six times, I think.

Interviewer

And during those sessions, was everyone focused while observing?

Student 1

Yes, that was the atmosphere in our group.

Interviewer

So you also had the experience of just observing, not as an evaluator or examinee?

Student 1

Yes, that’s right.

Interviewer

While observing, did any other thoughts come to mind?

Student 1

Since I observed in the same way as the observer, I think I got an overall perspective on how an observer should watch, and also how the examinees should perform during evaluation.

Interviewer

Was there anything your group generally did well or poorly?

Student 1

What we did well as a group was focusing on checking the patient’s condition. And what we missed, we still didn’t do well was maintaining a clean field. For example, we sometimes left alcohol swabs or waste containers on the bed, which isn’t proper for hygiene

Interviewer

Ah, then moving on to the exam. You took the exam at the end of the semester, right? What were the parts you felt confident in?

Student 1

Well, during the semester, we mainly focused on locating anatomical structures, so I felt confident in being able to explain those. I also felt confident in giving reasons for the points I located

Interviewer

Anything else?

Student 1

Other than that… hygiene. I felt confident in maintaining proper hygiene.

Interviewer

Hygiene covers a lot — what specifically do you mean?

Student 1

Hand disinfection for the physician, proper care of the treatment area, and maintaining a clean field. These are simple steps, but being able to do them properly made me feel confident.

Interviewer

Were there any parts you found difficult during the practical exam?

Student 1

I struggled a bit with the depth and direction of needling. The professor always told us to set the direction based on our own reasoning, but I kept wondering if the way I was needling actually matched my reasoning.

Interviewer

You mean whether your reasoning was valid?

Student 1

Yes. For example, whether I was trying to stimulate a muscle or a nerve, even for just one acupuncture point, the direction, position, and depth change depending on the structure. I wondered if I could explain that properly.

Interviewer

So because the structure being targeted changes with the direction, it was difficult to explain which structure you were stimulating?

Student 1

Yes, exactly.

Interviewer

So you mean that even while needling, it was difficult to clearly explain which structure was being stimulated?

Student 1

It’s not exactly that. It’s more that I’m still not confident about connecting the dots — whether to stimulate a nerve or a muscle to treat a shoulder disorder, or which structure to target for a specific condition. For example, if I were needling the shoulder, I’d want to say something like, ‘I will needle along the direction of the muscle to improve its movement’. But I’m not sure whether to use a perpendicular, oblique, or horizontal insertion. I had a lot of doubts about that, and that was something I felt I lacked.

Interviewer

Did you learn this in class?

Student 1

Sometimes it was mentioned, so I knew that I should explain it that way.

Interviewer

So you mean that depending on which structure you’re targeting and the treatment goal, the needling method should change, and doing that correctly based on reasoning was difficult?

Student 1

Yes, that’s right.

Interviewer

Were there any other difficulties you experienced?

Student 1

Other than that, nothing major.

Interviewer

Nothing major? Then, were there any similarities between the exam and the OSCE?

Student 1

Well, a big difference was that the evaluator was the professor. We could give more honest feedback among ourselves during the OSCE, but the professor’s evaluation was more precise and strict.

Interviewer

So you mean the professor’s feedback was more detailed?

Student 1

Yes, exactly. Also, in the OSCE we didn’t have to explain while needling, but in the exam we had to accompany the needling with explanations. Since I sometimes didn’t explain enough, it was a bit hard to match the evaluation criteria.

Interviewer

By explanation, you mean what you just mentioned — like explaining why you are needling?

Student 1

Yes. During the OSCE among ourselves, we just focused on disinfect, palpate, needle, and finish. But for the exam, for example at ST36, we had to locate it based on the tibialis anterior and explain step by step: ‘I’m dividing it this way using bony landmarks, and I will needle here.’ We were supposed to give these explanations each time.

But before the exam, there wasn’t a strong notice that we had to explain while needling. One of my teammates got a significant deduction for not explaining during the exam.

Interviewer

So the instructions about the exam weren’t very clear?

Student 1

They were mentioned once, but I wish it had been emphasized more that explanations must accompany the needling.

Interviewer

You mean giving explanations while palpating and needling?

Student 1

Yes, yes, exactly.

Interviewer

So what kind of feedback did you receive from the professor?

Student 1

I didn’t really get direct feedback.

Interviewer

The professor didn’t give you feedback?

Student 1

No, I was just listening to the feedback given to others. From that perspective, I thought if the professor had emphasized that giving explanations is important, students would have made fewer mistakes, because that’s part of the evaluation criteria

Interviewer

Out of the five acupuncture points you located, how many do you think you got right?

Student 1

Honestly, I think I located all of them correctly. I actually thought I didn’t do the needling part very well, but the professor didn’t seem to focus much on that, so I got a good evaluation.

Interviewer

Why did you think your needling wasn’t good? What part do you think you didn’t do well?

Student 1

I said I was going to stimulate the muscle, but then I did a transverse insertion, so I don’t think I actually reached the muscle at all. But the professor still gave me a good evaluation. So I changed my words and said, ‘For muscle stimulation, I’ll perform transverse insertion instead of perpendicular,’ and the professor didn’t seem to think it was strange. Maybe it was also correct, so I didn’t say anything more.

Interviewer

So, the needling was done on just one acupuncture point, right?

Student 1

Yes, that’s right. Out of the five points. The professor gave the instruction like, ‘After locating all five points, perform needling on one of them.’

Interviewer

Then which one did you do the needling on?

Student 1

It was on the shoulder — a point around the supraspinatus muscle, but I can’t remember its name.

Interviewer

Does the professor choose the point for needling?

Student 1

Yes, that’s right. After we locate the five points, the professor says, ‘Try needling this one,’ and that’s when we perform the needling.

Interviewer

Alright. Then after taking the exam, did you have any thoughts like, ‘I should study in a certain way from now on’?

Student 1

Yes. I realized that I need to study more so that I can clearly explain the reasoning and logic behind my treatments. The professor always emphasized that even for the same acupuncture point, the needling method should change depending on the treatment purpose. But we still don’t know much about that, and I hadn’t really thought deeply about the treatment purpose before. So when I tried to explain it to the professor, I found it really hard to put into words. That’s why I thought I should keep studying while always considering my own style and reasoning behind the direction or depth of needling.

Interviewer

If you study that way in the future, what kind of materials do you think you’ll use?

Student 1

I’ll probably rely mostly on the professor’s lecture materials and notes from class, but I also plan to look at other textbooks or research papers as reference.

Interviewer

In the professor’s class, did he often talk about the reasoning or logic behind acupuncture techniques?

Student 1

He mentions it sometimes. For really important acupuncture points, he explains that they’re often discussed in research papers, and that they’re related to certain anatomical structures. He also shows that there are various ways to use those points depending on the situation. He doesn’t go over all 361 points that way, but he does explain those key ones in detail, and that made me think I should study harder so I can understand and apply that kind of reasoning myself.

Interviewer

If the purpose of the acupuncture point training class is to develop the ability to perform acupuncture safely and hygienically on patients, to what extent do you think that goal has been achieved?

Student 1

In the case of hygiene, we really achieved 100%. But regarding the correct depth and direction of needling, students still seem a bit confused, so I’d say around 60 to 70 percent of the goal has been achieved.

Interviewer

Then how did you feel about the act of needling acupuncture itself?

Student 1

You mean when I actually inserted the needle?

Interviewer

Yes — the process of disinfection, piercing the skin, and inserting the needle.

Student 1

I don’t think I’ve quite mastered how to insert needles without causing pain yet. People still tell me it hurts when I do it. I think physicians who can make it less painful usually have very good technique or just a lot of experience. So I believe this is something that requires a lot of practice to improve.

Interviewer

Do you think the time for practice you get during the class is insufficient?

Student 1

I wouldn’t say it’s insufficient, but the time is pretty limited. We just made the best use of the time we had, but I do wish we could’ve had more practice time overall.

Interviewer

If you could increase the amount of practice time, how much more would you want?

Student 1

Right now, the class is three hours long, and the professor’s demonstration takes about an hour and a half. I think it would be better if that time were reduced a bit and replaced with a format where we practice together as the professor demonstrates. If the full three hours could be spent that way, students would probably stay more focused and have more opportunities to build good practice habits.

Interviewer

So instead of having a separate demonstration session, you mean starting practice right away while the professor moves around and shows the techniques as students work?

Student 1

Not completely removing the demonstration, but balancing it better. Since there are also good ways to use video demonstrations—like the real-time ones shown through a projector during class—I think we could adjust the ratio between the video demonstrations, the professor’s live demonstrations, and the hands-on practice.

Interviewer

When you say ‘video demonstration,’ do you mean the pre-class learning videos?

Student 1

No, I mean the live video demonstrations during class — the ones filmed with a camera and projected on the screen.

Interviewer

Ah, I see.

Student 1

Yeah, so not cutting them out completely, just reducing them a little.

Interviewer

I see. Then how much time do you think students should have for hands-on practice with each other?

Student 1

I think about two hours would be enough.

Interviewer

And after taking the acupuncture training class for a semester, is there anything you’d like to see improved or added?

Student 1

Yes — as I mentioned earlier, I really hope there will be more opportunities for the professor to guide us directly, right beside us, so we can learn through that experience. There was a particular acupoint on the arm — I can’t remember the name — but it had to be needled precisely between two tendons. When we practiced on our own, we felt stuck, but when the professor held our hands and placed the needles, the needles really went in, so I think if the professor helped us more at the side, we would have gotten better at placing the needles.

Interviewer

Is there anything else you’d like to add?

Student 1

No, I think that’s about it. I just hope the demonstration part could be shortened a little.

Interviewer

How about during the practice among students? Are there any parts you think could be improved?

Student 1

Actually, yes. I thought it might be better if there were more participants acting as patients. Right now, there’s usually one person receiving the treatment while four people take turns needling. The person getting needled often ends up in pain or discomfort, while others still want more practice time. So, I think it would be nice to have more patients — to balance the ratio a bit more between those practicing and those receiving the treatment.

Interviewer

Then, how many people usually practiced needling on one acupuncture point?

Student 1

Since we took turns practicing on each point, for example, if we studied 16 acupuncture points in one day, each person would practice on about four people, doing around four points in total.

Interviewer

So, each person did it only once per point?

Student 1

Yes, just once per point. Because if we inserted the needle more than four times at the same spot, the person would be in a lot of pain. So, we usually just insert it once and finish.

Interviewer

So, one person per point, then?

Student 1

Yes, just one person per point.

Interviewer

So you’ve never tried having two people take turns as patients?

Student 1

No, I don’t think we’ve ever done that.

Interviewer

Is there any rule that says you can’t have more than one patient?

Student 1

No, there isn’t. There’s no official rule about it. But within our group, we kind of agreed to stick to having just one patient each session. Unless the practice involved a particularly sensitive body area, we usually assigned only one person per week to be the patient.

Interviewer

I’d like to ask a bit about the practice note. Were there any aspects you found helpful while writing it?

Student 1

What I liked about writing the practice note was that it gave me a chance to review what we did, and I could document more concretely what I needed to observe during the practice.
It felt like there was some kind of checklist.

Interviewer

A checklist? Could you explain what you mean?

Student 1

I felt like there was a list of items, so I thought it would be good to check these things during practice.

Interviewer

So you mean there was a list of things to check during the practice?

Student 1

Yes, exactly. It felt like it served the role of a guidebook, which I found helpful.

Interviewer

Could you tell me more specifically what kind of items were on that list?

Student 1

Well, at the very start, things like checking the vitals, taking training photos, observing the patient’s reactions, and noting the physician’s sense of ‘deqi’—if we didn’t have the practice note, I wonder if I would have even thought about paying attention to all those things.

Interviewer

So it helped by pointing out what you needed to observe?

Student 1

Yes, exactly.

Interviewer

And while writing the practice note, were there any difficulties?

Student 1

Not really major difficulties, but the volume was a bit much.

Interviewer

Why was the volume so large?

Student 1

Because there were so many items to cover.

Interviewer

Did you feel like any parts could be removed or condensed?

Student 1

Well, since ‘deqi’ and the patient’s sensations were recorded separately, there was often some overlap. I thought it would be better to combine ‘deqi’ and sensations to reduce redundancy.

Interviewer

When you wrote about ‘deqi,’ what kind of details did you usually include?

Student 1

Well, for example, when I placed the needle at a certain spot, I’d note if a stiff area suddenly felt softer, or if the needle had difficulty piercing the skin layer, things like that. Sometimes I ended up writing similar observations repeatedly, so I wondered if it would be okay to reduce that.

Interviewer

So the physician writes down the ‘deqi’?

Student 1

Yes, the physician writes it. The patient usually just gives a brief comment at the start, like ‘It’s fine.’ After that, they rarely add anything.

Interviewer

So the physician writes both their own ‘deqi’ and their impressions of the patient’s reactions?

Student 1

Yes, since the practice note is basically the physician’s assignment, they end up writing down the patient’s responses too.

Interviewer

So when the physician asks the patient about their sensations and writes it down, it’s very similar to the ‘deqi’ notes?

Student 1

Exactly.

Interviewer

So ‘deqi’ is what the physician feels, and ‘sensations’ are what the patient feels, right?

Student 1

Yes, basically three things: the patient’s sensations, the physician’s deqi during the needling, and impressions after the treatment.

Interviewer

And the post-treatment impressions are also written by the physician? So you’re saying that the physician’s deqi notes and the post-treatment impressions are similar enough that they could be combined?

Student 1

Exactly. I know the professor’s intention might be a little different, but that’s roughly how we interpreted it, so it’s just my personal opinion.

Interviewer

Do you have any other suggestions about the practice note?

Student 1

Not really, nothing else.

Interviewer

Alright. We’ve gone through all the questions. Is there anything else you’d like to add?

Student 1

I think I’ve said quite a lot already, so nothing else comes to mind.

Interviewer

Alright then. That will be all for today’s interview. Thank you for your time.

**Interview with Student 2**

Interviewer

Well, you arrived quickly. So, did you know there's a manual for the acupuncture learning class?

Student 2

Yes, I did.

Interviewer

You’re somewhat familiar with what that is, right?

Student 2

Yes, but is the manual you mentioned the A4 sheet that the professor hands out before starting the class?

Interviewer

Yes, that’s right. It includes that, as well as example practice note uploaded on Google Classroom, the note templates, and also the equipment manuals and related materials. How did you first come across the learning manual?

Student 2

At first, when we started the class, the professor explained in detail how to use the manual, how to refer to it during practice, and the proper order of steps. After that, we just referred to it on our own without any additional instructions.

Interviewer

Then, how did you use the manual during class?

Student 2

During class, I mainly paid attention to the parts written in the practice note. The A4 sheet handed out at the beginning was just a simple reference to understand the day’s class order, but I mostly referred to the practice note. Since it’s a hands-on class, we’re expected to already know the name and location of each acupuncture point, and the anatomical structures we’re targeting with needling before the class begins. In addition to that, the practice note has sections for recording the physician, the patient’s sensations, and the physician’s own impressions during the procedure. So I thought it would be good to focus on those aspects during practice, and I would often jot things down right away using a tablet during the session.

Interviewer

I see. When you first started the acupuncture point training at the beginning of the semester, did you have a good understanding of what you were supposed to do during the class sessions?

Student 2

Not really at first. I honestly didn’t know much in the beginning. But the professor explained which parts were included in the practice note and recommended that we refer to it during practice. So, following the professor’s guidance, I started referring to the practice note from the very first session.

Interviewer

So, you mainly referred to the practice note. Could you describe in more detail how you used the sheet the professor handed out at the beginning of class?

Student 2

The printed sheet we received for practice didn’t really change much each week, except for the name of the meridian — like Lung Meridian of Hand-Taiyin or Large Intestine Meridian of Hand-Yangming. Other than the first week, when we practiced CNT and needling, the contents stayed mostly the same from the second week onward. It mainly included details like checking blood sugar, temperature, and how the patient was feeling that day. So I used it at first to understand what needed to be done during the initial sessions and to get familiar with the sequence of steps during the second and third weeks. From around the fourth or fifth week, since we already knew which acupuncture points we would be practicing, I would preview them in advance and used the manual just as a reference.

Interviewer

Thank you. Did you happen to use any other materials that were helpful during the practice?

Student 2

Do you mean something like the Handbook of Standard Acupoints?

Interviewer

No, I mean materials uploaded on Google Classroom — for example, instructions for handwashing or equipment manuals. Did you happen to look at those as well?

Student 2

Ah, yes. I forgot to mention that earlier, but the professor uploaded all of those materials at once before the class started. I went through all of them beforehand to get familiar with the contents. And during the first class, the professor explained everything again, so I remember that we all practiced handwashing together in class.

Interviewer

I see. Then, regarding the checklist or table that showed the class procedures in order — the one the professor handed out — did you have any difficulties following it?

Student 2

At first, yes. We had to check blood sugar, and the equipment for that, as well as the stethoscope, were kept in different places. The items and the instructions weren’t all organized on a single page — one sheet showed where the materials were, and another showed where the others. So during the first and second sessions, I relied on those a lot. But as I got used to the routine, I organized my workflow more efficiently and was able to proceed more comfortably.

Interviewer

I see. Then, as you mentioned earlier, do you think it would be more helpful if there were a material that showed at a glance where each item is located in the acupuncture practice room?

Student 2

Actually, I remember that the professor did make something like that. I think there was already a material for it. It listed where each item was stored, but it didn’t include an overall layout — like a floor plan of the acupuncture training lab. The manual indicates where the items are, and I know that the glucose meters are in this locker, but it’s a little confusing as to where this locker is in the lab. So I think it might be helpful if a simple diagram or image showing where those things are. I wonder if we could just add a quick note that says here are these things. But honestly, after about two weeks, everyone became familiar with the setup, so it wasn’t much of a problem after that.

Interviewer

Looking back over the semester as a whole, what did you find most positive or valuable about the practice sessions?

Student 2

Actually, I think the biggest benefit of the practice is that we’ve never held a needle before, so it’s good to be able to practice the most basic acupuncture skill, and it was really good to have the opportunity to practice basic needling techniques for the first time. Of course, you could technically try it alone at home after buying needles, but through the practice sessions, we were able to work with others every week, communicating as both the physician and the patient. Through that, I learned what to be careful about when performing acupuncture, and at first, I think I was really nervous at first putting the needle on someone else’s body, but I think it’s good to be able to practice it over and over again and get a little more comfortable with it. Also, it was great to locate the acupuncture points directly rather than just studying them from textbooks, and to realize that anatomical structures vary slightly from person to person. In our practice group, for instance, the members had very different body types — some were very slender, and others were a bit plump— so it was really helpful to experience palpation on a range of body types. Of course, that might depend on the members of each group, but for us, that variety was a real advantage.

Interviewer

You mentioned earlier about practicing needling. Do you feel that you had enough opportunity to practice needling throughout the semester?

Student 2

To be honest, I don’t think it was quite enough. For example, in our group, if we practiced 20 acupuncture points in one day, one person would be the patient, and the other four of us would divide the 20 points among ourselves — so each person practiced about five points. Of course, after we got more comfortable with the process, we sometimes took turns informally and practiced needling on each other while waiting for our turn. But since we divided the points like that, there were some acupuncture points we didn’t get to try ourselves, so I felt the practice wasn’t fully sufficient. Though that’s just how our group did it — I’m not sure how the other groups or different class sections conducted their sessions.

Interviewer

So you mean that only one person performed needling per acupuncture point, right?

Student 2

Yes. For instance, if we were practicing ST1, only one person would perform it on one side — since it’s a bit risky, we did it under the professor’s supervision, and only one person tried it. But for safer points, like LI4, everyone would gather and try inserting the needle on each other’s hands.

Interviewer

So, you didn’t just practice on the assigned patients — you also practiced on each other quite a lot during the sessions, right?

Student 2

Yes. But actually, that kind of active practice came later, after we had already built some rapport and gained enough experience as both physicians and patients. In the beginning, we just strictly took turns following the order.

Interviewer

I see. So one person performed the needling, but several people participated in locating the points, right?

Student 2

Right. For example, if A was the patient and B was the physician, normally B would do both the palpation and the needling. But as time went on, C and D, who were watching from the side, would start palpating each other’s bodies, saying things like, “Is it here?” and practicing that way too.

Interviewer

You mentioned earlier that you learned to communicate with others during the practice. Was there anything you learned or found difficult in that communication process?

Student 2

I think what most people would agree on is that even if you’re in the same group with close friends, it still feels different when that friend is approaching you with a needle. So it took some time to build that kind of rapport — the sense of trust that lets you feel comfortable letting someone work on your body. For me, that process took about two or three weeks. I happened to be one of the first to take the role of patient, so I experienced it early on. And—actually, this connects to something I wanted to mention later, about suggestions for the practice sessions.

Interviewer

Yes, of course.

Student 2

Should I share it now?

Interviewer

Sure, go ahead.

Student 2

During practice, we rotate as patients, right? I think most other groups did the same. Usually, the female students practice on areas with little exposure, like the arms or legs, and the male students practice on the abdomen or chest. Because of the practice order, in the beginning, only one or two, maybe three, people in a group would get to experience being a patient.

Being a patient for the first time — especially as a first-year student who isn’t a licensed physician — comes with a certain level of tension and nervousness. If I recall correctly, the first acupuncture points we practiced were LU1 and LU2 on the Hand-Taiyin Lung Meridian. Normally, you could start from the finger area at the end of the meridian, but we began here, and since it was the first session, the professor emphasized extreme caution. The professor explained that needling near the thoracic area could be risky, which naturally made the patients very tense. The physicians know it’s risky in theory, but they don’t feel it as intensely as the patients do. From what I’ve heard from friends, this isn’t unique to just LU1 and LU2 — the same feeling occurs with other points in the following weeks. Physicians may feel slightly nervous, but mostly they just insert the needles quickly, thinking, “This person must be fine.” That little tension is always there. So for suggestions about the acupuncture practice sessions, I think it would be good if, during the second week, everyone could get a chance to be a patient at least once, even if it means dividing it by regulation. Looking at the practice notes so far, most students realize only after being a patient what their peers must have felt — the fear and nervousness. After experiencing both roles as a physician and a patient, you can observe how much someone changes.

I think if everyone could be a patient during the second or third week, it would be really valuable. That was one point I wanted to suggest.

Interviewer

Thank you. I think there were also times when students practiced needling on their own bodies. Do you think it’s more helpful to practice by being a patient and receiving needling from others?

Student 2

Definitely. When you practice on your own body, you can feel the needle entering and naturally handle it more carefully. But having someone else insert the needle feels completely different. If it were a licensed physician, you might trust them because they’re experienced and skilled. But when it’s your classmates, your peers who are going through the same training, there’s a different dynamic. That’s why I think it’s really important for everyone to experience being a patient at least once before starting. Of course, I want to add that I really trust my group members.

Interviewer

Thank you. I’d like to ask a bit more specifically about your experiences as a physician.

Student 2

You mean the difficulties and insights you gained while performing needling?

Interviewer

Yes, exactly.

Student 2

First, one thing I realized during practice was that I was one of the first in my group to be a patient, and then the following week I was practicing as a physician. Doing that, I really understood that being a patient isn’t easy — trusting someone else, who’s holding a needle, to work on your body is not simple. After that, I really recognized the importance of communication. I focused a lot on communication during practice, and I noticed that when the patient expressed themselves clearly, the tension they felt was different. From that, I understood that in future situations, whether I run a clinic or work as a salaried doctor, communication with patients is crucial — because the patient might be receiving acupuncture for the first time, and the same principles apply.

Another thing, just from my opinion, when palpating other people’s acupuncture points, I noticed that many points were in softer areas rather than rough parts. So when inserting needles, I paid special attention to those softer areas, wondering whether they were indeed acupuncture points and focusing on the characteristics of each point.

Since we only practiced each acupuncture point once, and didn’t repeat them the following week as a review, there’s some inherent risk. By the last weeks, we were getting more comfortable, but for the points themselves, it was our first time, so I tried to maintain caution — checking for potential risks, like bleeding or proximity to the thoracic cavity — and kept my focus throughout. On the other hand, I noticed some of my classmates starting to relax too much, which I couldn’t openly point out, but it was a little disappointing.

Also, since this was practice, it felt limited in that we were only learning point locations and palpation techniques, rather than actually treating a patient. But I understand that expecting real treatment would be unreasonable. Another limitation was that the professor could only monitor the whole class and couldn't work with each group individually, so we had to check each other's work—that left a lot to be desired as well.

Still, it was helpful that photos were taken and the professor provided comments in the practice notes afterwards. That part was really useful.

Interviewer

I’d like to ask a bit more specifically. You mentioned that you realized the importance of communication and that when the patient is informed, their tension is reduced. How exactly should a physician communicate to help ease the patient’s tension?

Student 2

Well, when I’ve been to an acupuncture clinic, the physician doesn’t explain everything in detail. They might just locate the muscle or point quickly. But in our practice sessions, we know the whole process in advance and are expected to record it in the practice note. So when I tell the patient, “I’m going to insert a needle at this point, and I’ll follow this process to locate it, while being aware of possible risks,” the patient can see that I’m aware and careful. That tends to reduce their tension.

Also, the physician’s calm demeanor is really important. If the physician is nervous, thinking, “Can I do this safely?” the patient can pick up on that and start thinking, “Is this person doing this correctly?” Even if I’m nervous inside, I try to remain as calm and focused as possible. This both reassures the patient and helps me perform the needling properly. I paid a lot of attention to this balance.

Interviewer

So you felt the importance of communication even more while being a patient? How did you come to realize that?

Student 2

I really noticed it while being a patient. The first time I was a patient was very early in the semester, and the next time I was a patient was much later. By then, I could clearly see how differently people behaved, and I could feel how my own tension changed in response. That was the first thing I realized.

The second thing I noticed was when I was the physician, the patient’s facial expressions immediately reflected their level of tension. For example, when I mentioned LU1 and LU2 on the Hand-Taiyin Lung Meridian, I said the patients were really tense — and I happened to be the physician for those points. Because I was tense myself, even my classmate, who was a close senior and the patient, seemed a bit nervous. At that time, we called over the teaching assistant and received guidance and comments. After adjusting my approach and adopting a calmer attitude, I could see my senior relax and feel more reassured.

Interviewer

I see. You mentioned that toward the later part of the semester, the tension tends to ease. Could you explain more specifically what you mean by that?

Student 2

At first, since we were inserting needles into someone else’s body, we could immediately sense how it might feel if it were our own body. Everyone was nervous, and even the way we handled the needle — the speed of insertion, for example — was different. Ideally, practice should take place in a relaxed atmosphere, but sometimes it can go beyond relaxed into too casual, where people start slacking off a bit.

Also, we all did some preparation before class, and that helped maintain focus. Toward the later sessions, though, some students would come less prepared. For instance, if a group was assigned four points, they might have practiced one point very carefully, but be more casual with others. I personally stayed meticulous, but I could see that some tension had eased among classmates.

It’s hard to describe exactly, but even if someone complained about pain, it sometimes seemed like they weren’t fully serious — something like, “It’s okay, just rest, and you’ll be fine,” which might be true, but it was different from the kind of reaction we saw at the beginning of the semester. So the easing of tension meant both a change in focus and in how seriously people responded to sensations.

Interviewer

In the practice lab, you’re supposed to use formal speech, right? Do you think that was generally followed?

Student 2

Well, in our group, we didn’t really use formal speech consistently. Even though the observers would leave comments in the observation notes every week — for example, “We should maintain some formality and act like we’re with actual patients” — our group had a wide range of ages. There were several transfer students, and I was the youngest, so we didn’t use formal speech very much.

Also, as the semester went on, we got closer and more familiar with each other. Personally, I think, maybe as an excuse, that being in a friendly atmosphere helped ease tension. We did try to consciously use formal speech a few times at the start, but it often felt awkward, so we ended up just speaking more casually.

Interviewer

I see. While going through the practice sessions, was there anything you felt you learned in a particularly meaningful way?

Student 2

Meaningfully……

Interviewer

Something that you feel you can actually do now?

Student 2

Well, the biggest thing I realized from the professor’s class is that I now feel confident about performing CNT. During the vacation, I had a chance to practice needling with other students — from other Korean medicine colleges, and so on — and I realized that when it comes to my CNT, I wouldn’t fall behind anyone. Honestly, I feel I can perform CNT so thoroughly that I’ll never forget it for the rest of my life.

Interviewer

What made you feel confident about CNT? Was it mainly because of how the professor taught it?

Student 2

Let me see… The professor really emphasized CNT during the first session, and it’s also covered in theory and practice, so we did a lot of repetitive learning. That was the first reason.

Second, the professor consistently stressed CNT whenever teaching, so we students could clearly understand its importance. I also remember that on the first day of practice, each student had to wash their hands for 30 seconds in rotation, which was really memorable. Everyone washed their hands thoroughly, and if washing wasn’t possible, we made sure to sanitize with hand sanitizer.

In the practice notes, there are many sections for observers to check regarding CNT. Since we rotated as observers every week, we received immediate feedback on what to pay attention to. Now I know what it takes to do a procedure on a patient in a more stable environment, I can do it if I follow the same steps as in the practice setting. Personally, checking how CNT was performed in my group every week helped me internalize it. It’s a great way to learn more about CNT.

Interviewer

Since you mentioned being an observer, I’d like to ask what you felt while performing that role.

Student 2

While observing, you can notice strengths in others’ practice that you might not yet have yourself. For example, “That person has a calm attitude and makes the patient feel comfortable,” or “This person is confident,” or “They’re using a particular needle guide tube — I should try that too,” or “They’re very thorough when there’s bleeding.”

In our group of six, if you exclude the patient, four people are physicians. Watching everyone for an hour allows you to pick up these strengths. Of course, in the first observation session, everyone was a bit inexperienced, so it could feel somewhat chaotic. But once everyone became more accustomed, observing allowed me to absorb each person’s strengths into my own practice.

Also, observing gave me a chance to reflect on my own performance: “Did I do it that way?” or “I should adjust what I did before.” It was very effective for self-feedback.

Interviewer

After observing, did you notice any changes in how you performed as a physician?

Student 2

Yes. Before, my approach wasn’t exactly calm — I mostly tried to create a friendly, comfortable atmosphere. But in our group, there was someone whose hands were really cold, yet their demeanor was very composed and precise, and they spoke in a calm, reassuring voice. I thought, “That’s effective.” So the next time I practiced as a physician, I tried to adopt that approach. I noticed that when the patient joked around, I could respond playfully to build rapport, but when performing needling, I could shift back to a calm and composed attitude. It was a valuable experience to see how my demeanor could adjust depending on the situation.

Interviewer

I see. Earlier, you mentioned feeling nervous or tense as a patient — but aside from that, were there any other things you noticed or experienced?

Student 2

Can I also talk about what was difficult as a patient?

Interviewer

Yes, of course.

Student 2

So, when I was the patient, sometimes I got needling about ten points, but on some days, it could be as many as fifteen or even twenty. Getting that many needles in one session was quite a lot. And, well, from the few times I’ve been to a clinic — I’m generally healthy, so I don’t go that often — I’ve noticed that physicians there tend to use relatively short needles. But in our class, the professor emphasized accurate targeting of anatomical structures and appropriate depth for each acupuncture point, even for deep insertions. My classmates were also very passionate, so the needles often went in quite firmly. Because of that, I think my body actually felt quite tired after each session. I’m not particularly weak — I don’t run fast, but I do have decent endurance — yet still, for about three days after practice, I’d feel quite fatigued. And it wasn’t just me — both female and male members said the same thing, that after being needled, they felt drained. That part was probably the hardest for me.

Interviewer

Then, have you ever divided the role of the patient among multiple people during a single practice session?

Student 2

Yes, I’ve tried it with up to two patients at once. At first, we practiced one-on-one, and in the early sessions — like when we started with the Lung Meridian of Hand-Taiyin — there weren’t many points to needle. But as we moved on to meridians that pass through the trunk area, the number of points increased. There was one time when one of the male members got needling more than twenty points in one session. Even though he was really sturdy and in good shape, he said it was quite exhausting. So after that, we started dividing the patient role between one or two people, especially as exam season approached. Even when there weren’t many points to practice, we still tried to share the role to make it easier for everyone.

Interviewer

And were there any other difficulties you experienced during the practice?

Student 2

I think I felt quite tense when needling points like ST1 near the eyes, or points below the sternal notch — where there’s a risk of injuring the trachea or puncturing the thoracic cavity. Those anatomical areas made me quite nervous. Also, when teaching assistants were present, I could immediately get feedback like, “That insertion angle isn’t ideal,” which helped a lot. But when that guidance wasn’t available, I often felt more anxious.

And for points that require deeper insertion — maybe not extremely deep, but still relatively deep — it’s hard to check whether there’s bleeding in the deeper tissue. If I were needling myself, that wouldn’t worry me as much, but when performing on someone else, it’s different. Since I’m not yet a professional and can’t check with ultrasound every time, I sometimes wondered, “What if there’s bleeding at deep area?” That thought weighed on me a little. But aside from that, I don’t think there were any major difficulties.

Interviewer

Some students mentioned that writing the practice notes was a bit difficult. What do you think about that?

Student 2

Writing the practice notes for every meridian is definitely not easy, because this isn’t the only course we have assignments for. When things get busy, I sometimes think, “I didn’t really have any particular realization for this session — what should I even write here?” It also takes quite a lot of time.

But still, by writing the practice notes, I went through all the required points carefully. At first, I used to keep the practice note open next to me during practice, thinking, “Ah, these are the things I need to pay attention to.” And once I got used to it, I started naturally checking all those details during the session. So even though it’s hard and time-consuming, I think it’s quite meaningful because it helps me avoid missing things and be more thorough. In the end, despite the difficulty, I believe we gain a lot by writing the practice notes.

Interviewer

I see. Then, when did you usually write your practice notes?

Student 2

Sometimes I’d keep my tablet or laptop open next to me and write them right away during the class. One of my group members even asked for the patient’s consent and recorded short voice memos — not videos, just audio — to capture their thoughts and impressions.

Since it’s easy to forget details afterward, we tried to record things as soon as possible. Usually, we’d prepare the document in advance so everyone could access it and type things in right away during practice. Then, I’d polish the wording and organize everything more neatly on Sunday before submitting it.

Interviewer

I see. Then among the practice note sections, were there any items you felt were redundant or somewhat tiring to complete?

Student 2

I think it was when I was the observer — or maybe the patient? I’m not entirely sure now — but there’s a section where we write down our impressions. That part sometimes overlapped with the final section at the bottom, where we summarize our overall reflections. So, I thought it might be okay to omit one of those since the contents were often similar.

Also, for each acupuncture point, there’s a space to write what we felt, and that often connected directly to what we wrote in the final overall reflection. It was a bit difficult to express it differently every time, and I felt that when reviewing the practice note later, it might be enough to just read the final reflection section.

And about the Deqi sensation—when the professor taught us, he said there's the physician's Deqi and the patient's Deqi. But there's also a separate field for the patient’s impression, which seems a bit redundant. The Deqi sensation section should specify whose sensation we're documenting. Even though there's a separate area, recording the Deqi I felt when needling and describing it feels like overlap. I think that section needs some reorganization.

Interviewer

Yes, besides what you mentioned earlier — that writing the practice note helped you observe things more carefully and thoroughly — was there anything else you felt you practiced or became more capable of doing through it?

Student 2

Do you mean something that came specifically from writing the practice note itself, or…?

Interviewer

Yes, I’m curious whether you think the process of writing the practice note had any particular meaning or value for you.

Student 2

Ah, I see. Would it be okay if I open the practice note form for a moment?

Interviewer

Of course, yes. You can check the one you filled out before, too.

Student 2

Okay, just a moment.

Interviewer

You mentioned earlier that although writing the practice note took a lot of time, there was still a lot to gain from it. Could you tell me a bit more specifically what those benefits are.

Student 2

Just a moment — I’m opening the practice note file right now...

The observer section has items to check regardless of whether you're the observer, patient, or physician. Sharing these observations helped us clearly identify what needs attention. The physician performance and communication sections were especially valuable because I got objective feedback on my technique. Our group members wrote lots of compliments but also gave honest, constructive criticism, which had been incredibly helpful.

And honestly, I didn't look at it much when it wasn't exam period, but when preparing for practice exams, I got through everything. Looking back, I could see how much my attitude, technical skills, and experience had improved, and which parts still needed work. I could also see others' insights, which allowed us to share insights and discuss them together — I found that really valuable.

And, um... the point locating process sections were very helpful. Sometimes when I was thinking 'What acupoint was that again?', I looked at the point location process that group members had written down, and it was like 'Right, there!' and it immediately came back to me. That was really useful. Also, writing down needle specifications and depth—'this point only needs to go this deep' or 'this one needs to go deeper'—since the professor really emphasized anatomical structures and the risks of needling, that was very helpful too.

Then for the target anatomical structures during needling, we usually previewed before coming to class, right? So you might just think 'oh, there's an acupoint here,' but the process of checking 'what anatomical structures might be related here?' was really good.

But honestly, we didn't remember everything like gathering all that information and saying 'this meridian had these effects.' That might be more meaningful later on, but anyway, since this was acupuncture point practice, I think it was most helpful in finding accurate locations, which is what the professor intended.

Earlier I mentioned that the Deqi sensation section could be reorganized — since the patient’s and physician’s sensations sometimes overlap — but apart from that, the “feeling during needling” part was quite meaningful. It helps recall what I experienced when needling that point, which is useful later when I practice at home. The photo section is good too. That said, for points that share similar parameters — like those along the Conception Vessel meridian — it can feel repetitive to write the same needle direction, angle, and depth again and again. When we study for exams, we tend to group them by body region (chest, abdomen, upper area, etc.), so organizing the practice note that way might be more efficient. But since we also added some of the sections ourselves, not everything can be standardized that easily.

Personally, the section I reflected on most was the self-reflection part. It’s where I could review what I had done that week, but also, since there were six of us in a group and everyone noticed different things, we could all see each other’s reflections in real time. So even if I had missed something, I could learn from what others noticed.

Honestly, I think the best thing about the practice note is this: if doing it alone might give you a 60 or 70 out of 100, doing it together and sharing different viewpoints could get you over 80. Sharing those insights from group members was the most valuable part of the practice note for me."

Interviewer

Thank you. Now, you briefly mentioned the exam earlier — you took it during the final week of the acupuncture training class, right? I’d like to ask what aspects you felt most confident about at that time.

Student 2

What I felt confident about... well, I’ve forgotten quite a few points by now since the exam’s over (laughs), but at that time, I could locate all the points within the exam scope right away. I remember thinking, “I can find them quickly,” especially since the professor said the time would be measured.

Also, I referred back to the practice notes a lot — particularly to the point location process. I wanted to make sure I wasn’t just guessing the points vaguely but could really identify them based on anatomical structures and landmarks. Our group practiced that together a lot, checking whether we were locating each point properly.

So I guess, I could say I was confident in my ability to accurately find and locate the acupuncture points — though it feels a bit embarrassing to say that now (laughs).

Interviewer

Not at all! Was there anything else you felt confident about?

Student 2

Hmm… what else could there be… let me think…

Interviewer

Earlier, you mentioned things like patient communication, hygiene, and CNT — those seemed to be points you were confident about.

Student 2

Actually, those would fall more under the areas I wasn’t confident about during the exam.

Interviewer

Oh, really? In what way?

Student 2

So, I mentioned before that I usually follow CNT very strictly. But when I entered the exam room — with the professor dressed neatly, all the equipment around, and the examination table in the middle — I got really nervous. I was actually the first to go among my group, and it was the first morning session, so I didn’t even get a chance to hear how it went for other students. It really felt like being thrown into the deep end.

I did wash and sanitize my hands, but it wasn’t like, “Okay, I must perform CNT carefully now.” It was more like my body just did it automatically, almost out of habit — maybe that’s a good thing in a way, since it’s become natural, but I still didn’t feel like I did it well.

As for patient communication, since we were paired with the same members we had practiced with throughout the semester — not new people from other groups — it didn’t feel awkward or nervous during the exam. I think I just did everything in a very routine, unconscious way, rather than consciously thinking, “I need to communicate like a real doctor right now.”

So, to sum up, I think the parts where I couldn’t fully show my usual performance — because of the exam tension — were mainly the CNT, the patient communication, and explaining the point-location process out loud.

Interviewer

It sounds like it was difficult to perform as you normally would, because of the tension.

Student 2

Yes, exactly. I honestly thought I wouldn’t get nervous at all, but it turned out to be much more nerve-racking than I expected. Looking back, I didn’t actually make any big mistakes — like locating the wrong point or failing to insert the needle — but when I recall the exam, I noticed something. The students in the group before mine were verbally describing every single step of the point-location process.

In our group of three, though, both I and the second person barely spoke during the point-location process. The third person did explain verbally, but her voice was trembling the whole time — I still remember that clearly.

And, for example, when you divide the abdomen into 8 cun and then keep halving it, usually we just divide it intuitively with our hands, not by actually measuring with a ruler. So, describing that process out loud in real time was quite difficult.

Personally, I think the professor might have been focusing more on our actions than on our verbal explanations — since he could probably tell whether we were palpating and locating points correctly just by watching our movements. Still, I definitely felt that explaining the process verbally during the exam was quite challenging.

Interviewer

So during the exam, did you perform both the five point locations and the needling on one point?

Student 2

Yes. In the exam, five points appeared on the screen — I explained four of them, and then performed needling on one that the professor selected. There was also a bonus question. Other students told me later that they explained all five points first and then did the needling at the end, but I thought the correct order was to explain four, perform the needling, and then move to the bonus question. Because of that, I didn’t explain the bonus one, even though I actually knew it. It was a bit disappointing.

Interviewer

I see. And later in the semester, you also did the OSCE, where you acted as an observer evaluating another student’s needling, right? I’d like to ask about that experience too — did you feel nervous then as well?

Student 2

I think I was a little nervous that time too. After all, I was evaluating someone else, and there was a time limit and specific scoring criteria — like communication with the patient and so on. So I did feel some tension, but I tried to stay as calm as possible while doing it. Still, I think there’s a big difference between when the professor is present and when they’re not.

Interviewer

When you did the OSCE with your group members, was there anything you found particularly good or helpful about that experience?

Student 2

Personally, I had actually looked at the OSCE evaluation criteria once — maybe a week or two before — because I was assigned as an observer. But before that, I hadn’t checked it at all. I felt like since OSCE was an evaluation, rather than preparing for it like an exam, I wanted to just perform as I normally would and then identify my weak points through the process. So that’s how I approached it — I did it as usual and then tried to correct what I found lacking.

Interviewer

Do you remember what kind of weak points you noticed?

Student 2

Just a moment — I actually took a photo of the feedback… Yes, it seems my main issue was that I was a bit slow. Other than not finishing within the allotted time, everything else was okay. My group members gave me feedback that I took a bit too long to check for deqi sensation or to ensure safety. So I wondered if I might have been too overly cautious about safety. I realized that while making the patient feel comfortable is important, I also need to maintain a certain level of focus on the procedure itself. After that, I tried to adjust my approach accordingly.

Interviewer

Then, is there anything you’d like to see improved or changed about the acupuncture training class in the future?

Student 2

I actually mentioned this earlier — during the second week, I think everyone should really get the chance to be the patient at least once. It could be before starting the Lung Meridian of Hand-Taiyin, or even using that meridian, but I think before we begin, we could use some of the safer acupuncture points — like those on the arm muscles, or points such as LI4 — and spend about an hour giving everyone the experience of being the patient on the bed. That first experience of lying down and feeling nervous is something everyone should have early on. Only then can people truly understand how the patient feels.

And, well… this isn’t exactly a strong request, but—

Interviewer

Oh, please go on.

Student 2

Um, actually, will the professor see this video?

Interviewer

No, no — absolutely not.

Student 2

Oh, then in that case...

Interviewer

Yes, please go ahead. When we write the paper, we only summarize the responses — like using general phrases, not direct quotes — so they won’t really see your exact words.

Student 2

During the final exam, the professor mentioned that female students, in particular, should be cautious about exposing areas like the chest or other sensitive regions — that even though it’s approached medically, exposure should be handled carefully. But in the actual exam, we only need to expose one of the five tested areas. So, I thought that maybe for that one point, a female teaching assistant could come in briefly to observe, since the professor would still be the main evaluator. It wasn’t just my idea — some classmates and I talked about it together.

When I later looked at the spreadsheet, I noticed that most of the pairs were actually same-gender — female with female, male with male. I think that’s probably related to what the professor said about exposure, especially around the chest or abdominal regions. But if you think about it, there aren’t really many risky points on the abdomen, and since in the exam we only needle one of the first four points, not the last major one, I thought it might actually be fine to have mixed-gender pairs.

Looking back, even when I practiced with female classmates, we never really had to expose any major areas. So, I started thinking that it might be okay to work in mixed-gender pairs after all. Also, during regular practice sessions, time was limited, but when we prepared for the exam, we could take more time — discussing whether our point location and needling were correct — and I think that’s when my understanding of the acupuncture points improved the most.

However, if we keep separating by gender, we miss the chance to understand differences like male muscle tone or female softness, which would actually be helpful for future clinical situations. Since in real practice we’ll be treating both men and women, I think it would be better to have mixed practice.

That said, the professor emphasized modesty and being careful about exposure, so it made me wonder — is that really still such a big issue in actual clinical settings?

Interviewer

"Do you mean to what extent the body is exposed?
Speaking a bit outside of the interview for a moment — just from my personal experience — I think many patients are actually quite comfortable with exposure.
The patients I’ve seen, for instance, even at points like CV17, didn’t really hesitate about it. Maybe it’s because I’m a woman, but even my female patients are quite open to receiving acupuncture in those areas.
And there are also female patients who don’t mind undressing in front of male physicians. Of course, it’s more common among older women, but even younger ones sometimes do so without much hesitation.

And the practice uniforms for acupuncture training classes are designed with zippers, so essential areas can be kept covered pretty well. Still, I think physicians — Korean medicine doctors — need to be mindful of that and make sure to help cover patients properly. That kind of professional attitude and awareness is important too.

But actually, I think I might not have understood what you meant earlier. When you said that the professor emphasized exposure — did you mean that the professor was saying it’s a problem if female students try too hard not to expose themselves, or that because they shouldn’t expose themselves, female students should practice only with other female students?"

Student 2

It’s closer to the first one.

Interviewer

So, you mean the professor doesn’t like it when female students try too hard not to expose themselves, because that makes the practice more limited, right?

Student 2

Right, right. Sorry, I think I explained it a bit confusingly. How should I put it... Before the exam, there was a briefing session that the professor gave. And at that time, it was quite surprising — I mean, of course, the professor wasn’t going to tell us to needle points like ST17 or anything like that.

**Interview with Student 3**

Interviewer

Okay, you took the acupuncture point training course for one semester. First, are you familiar with our acupuncture training learning manual?

Student 3

Not exactly. Before the semester started, I came across some manuals shared by the professor through Google Classroom or similar platforms — things we needed to know beforehand, like handwashing, using materials, or cleaning procedures. After the semester began, I learned about more practical topics, such as precautions during practice, safe needling techniques, checking the patient’s condition, and CNT-related guidelines. I learned about these mainly through lectures and materials uploaded on Google Classroom, as well as through the professor’s explanations during class.

Interviewer

You mentioned that you saw those materials before the semester started. You also mentioned manual for using the equipment — did you happen to read them before the class began?

Student 3

Yes. I didn’t read them thoroughly, but I did look through them.

Interviewer

While reading them, did you feel anything in particular or learn something new?

Student 3

Yes, things like waste disposal were all new to me. Since this was our first time using the acupuncture training lab, everything related to that was new. Before, I only knew about simple things like handwashing, but I wasn’t familiar with the materials or equipment used in the lab.

Interviewer

You mentioned the equipment in the lab earlier — did reading about it help you?

Student 3

Yes, I think it helped me a lot.

Interviewer

In what way?

Student 3

I got to know the locations of the equipment, and during class, I had been confused about where to dispose of certain items, like medical wastes. Reading the section about material classification helped me understand that. Also, the professor emphasized CNT a lot, especially regarding disposable needles — that they should be used once and discarded immediately — and I learned about those procedures through the manual.

Interviewer

Since we’re talking about equipment, I’d like to ask one more question. Did you use the cart next to the bed during class?

Student 3

Yes, I used it a lot. I mainly used it for items to be discarded, and also placed all the tools needed for practice on it. I often moved the cart when changing positions, and sometimes used it to place the iPad or other items while practicing, so I think I used it quite frequently.

Interviewer

Now, moving on to your overall acupuncture training — at the beginning of the semester, around September last year, did you have any difficulties?

Student 3

In terms of training, yes. It was my very first-time performing acupuncture, so when I inserted the needle and the patient reacted, I flinched as well. I couldn’t tell whether the patient was really in pain or if it was a dangerous situation, so I was very flustered at that moment. Also, during class, we learned about acupuncture points and related theories, but when I looked at the patient’s body, I realized that the structures, like bones, differed a lot from what was shown in the textbook. That was quite challenging for me.

Interviewer

How did you overcome those difficulties?

Student 3

Mainly by practicing a lot. I think repeated practice helped me the most. Besides that, I tried to study the theoretical parts in the textbook more diligently. During training class, when there was a patient, we would insert needles into the patient’s acupuncture points, and while locating points, we explained each step to the other group members. Through that process, I was able to understand the anatomical structures much better.

Interviewer

You mentioned that you explained things to the patient while locating points. In what way do you think that helped you?

Student 3

By doing that, I was able to identify acupuncture points that differed slightly in location from person to person. Also, although muscle positions varied more than I expected and I couldn’t always clearly distinguish their boundaries, I could at least understand roughly where each muscle was located.

Interviewer

When the patient reacted to the needling, you mentioned earlier that you felt some worry or anxiety as the physician. As the practice went on, how did that feeling change? Did it continue?

Student 3

Yes, I still felt that way to some extent, but during every class, the teaching assistants or the professor were always nearby. So if something happened — if the patient experienced any safety issue — they immediately noticed and handled the situation. Because of that, I felt psychologically reassured, and it was okay.

Interviewer

Did you ever receive instructions like, “If the patient shows a certain reaction or condition, you must inform the professor or the assistant”?

Student 3

Yes, they always emphasized that.

Interviewer

How were you instructed about that?

Student 3

The professor always explained in great detail what kinds of reactions a patient might have during needling. They mentioned it every time during class. I actually experienced dizziness once during needling, and at that moment, I immediately realized, “Oh, this is a sign of fainting” so I reported it right away to the professor and the teaching assistant.

Interviewer

Then, besides dizziness, in simpler terms, what kinds of symptoms or reactions did they say would require you to ask for help from the professor or the assistant?

Student 3

Usually, if the patient looked anxious, or said they didn’t want to continue the needling anymore — in cases where it seemed difficult for the physician to go on — we were told to immediately inform the professor or the assistant. Also, according to what I heard from classmates, if the patient didn’t feel very well the next day or afterward, they were told to contact the professor right away. Then the professor would explain what to do and how to manage the situation afterward.

Interviewer

During class, did you ever feel like, “I don’t know what I’m supposed to do”?

Student 3

In terms of practice, not really. Since practice sessions aren’t something we get to experience often, and at that time I was still a first-year in the main course, I actually wanted to practice as much as possible. So even when there was extra time, I would use it to try needling again on areas I was curious about. Because of that, I rarely had any idle time during the sessions.

Interviewer

At the beginning of the course, the professor gave out a kind of learning plan table — like a timetable showing what students should do and what the professor would do for each group. Did you refer to that during class?

Student 3

Yes, I did refer to it, but the practice sessions often lasted longer than we had planned. Since we already had our own goals for each session, we just continued until we met those goals. So I used the plan mostly as a reference rather than following it strictly.

Interviewer

Okay. You may or may not remember the teaching manual, but it was structured into sections like before class, during class, and after class — kind of like introduction, development, and conclusion. I’d like to ask about each of those parts in a bit more detail. Before class, the professor asked you to do some pre-learning, right? There were videos and such. Also, in the *practice note*, there’s a section to write down the *point location* procedure beforehand.

Student 3

Ah, yes.

Interviewer

I’m curious whether you actually did that — how did you do your pre-learning?

Student 3

Yes, I did the pre-learning related to the *point location* process. We always referred to that during practice, so I did prepare it in advance. I also took notes while listening to the professor’s lectures and PPT slides. Then, during needling practice, I reviewed those notes and observed how nerves responded and things like that.

Interviewer

So mainly, you watched the pre-learning videos and organized the content from them?

Student 3

Yes, I did.

Interviewer

And when you wrote down the *point location* procedures in the *practice note* beforehand, you referred to those notes a lot during practice?

Student 3

Yes.

Interviewer

When you actually practiced, did you notice anything in your notes that was different from reality, or perhaps lacking in detail?

Student 3

I didn’t find the content lacking, but one difference was the needle insertion depth. In the textbooks or pre-learning materials, the depth was generally shown as shallower, but the professor sometimes inserted the needle deeper than expected, so that was a difference.

Interviewer

So the pre-learning videos showed shallower needle depth, but during the professor’s demonstration in class, the needle was inserted deeper?

Student 3

During the lecture itself, it wasn’t shown much, but if you look at the materials, they include the insertion depth. That’s where the difference came in.

Interviewer

You mean the PPT materials from class?

Student 3

Yes, I also referred to those for guidance on depth.

Interviewer

So the PPT showed a shallower depth?

Student 3

It varied, and usually the difference wasn’t large, but occasionally there were discrepancies. In those cases, the material showed a slightly shallower depth.

Interviewer

So when you say the depth in the materials was shorter, are you comparing it to the actual practice?

Student 3

Yes. When the professor inserted the needle, they would often comment, “It looks like it’s gone in about this much.” But it actually went deeper than I expected. I thought, for a risky area, following the textbook and inserting less would be enough. However, the professor could insert it deeper without any problem, so I realized that in some cases, it’s okay to go a bit deeper than what the material shows.

Interviewer

Are you referring to what the professor demonstrated during class?

Student 3

Yes, during the class demonstration.

Interviewer

Can you think of a specific example?

Student 3

I don’t remember the exact acupuncture point, but what left an impression on me was the idea that “for certain points, don’t insert too deeply because they overlap with nerves.” But the professor actually inserted it deeper than I expected. I asked about it, and the professor explained, “In clinical practice, it’s done this way.” So I realized that the insertion depth isn’t strictly standardized and can vary depending on the patient.

Interviewer

I’m also curious — in the PPT, is the needle insertion depth shown in centimeters?

Student 3

No, it’s shown in millimeters or in *cun*. I think most of it was in *cun*.

Interviewer

Did you convert the *cun* into millimeters or centimeters?

Student 3

Yes, I did that conversion.

Interviewer

Then, how many centimeters did you use for 1 *cun*?

Student 3

I think it was around 2.78, or roughly 3 centimeters.

Interviewer

Okay, now moving on to the training class. The preparation time — that is, the time from entering the acupuncture training lab until receiving the professor’s demonstration — was set at about 15 minutes. This included bringing materials, washing hands, and so on. Was that time sufficient?

Student 3

I don’t think it was really enough. At the end of class, when everyone comes in, there’s often a long line for handwashing, so that took quite a bit of time. We also measured vital signs again after meditation, so overall, I felt the time was a bit short.

Interviewer

You mean you measured vital signs after meditation?

Student 3

Yes. Normally, it should have been completed earlier, but we didn’t have enough time.

Interviewer

During the 15 minutes of preparation, did you ever feel like, “I don’t know how to get ready”?

Student 3

At the very beginning, yes. But after practicing a few times, I got used to it, so it was fine after that.

Interviewer

Now, moving on to the professor’s demonstration. During the demonstration, what do you feel you learned?

Student 3

Rather than learning specific techniques, it was more about realizing the level we need to reach. The professor quickly finds acupuncture points and inserts needles with almost no hesitation, whereas we take a long time to locate each point and often wonder if it’s exactly right. Watching the professor, I felt that I need to practice diligently. Also, when I insert needles, I always feel nervous and a little scared, wondering how much depth is appropriate. Seeing the professor use their own method confidently made me think I should develop my own system too. That aspect left a strong impression and I feel I learned a lot from it.

Interviewer

How well did you understand the content of the demonstration?

Student 3

It wasn’t so much understanding as it was reviewing what I had studied beforehand. It gave me a sense of how things should actually be done, and I focused more on comparing the differences from what I already knew.

Interviewer

Focusing on the differences from your pre-learning, the professor’s demonstration can be divided into two parts: locating points and needling, and showing structures with ultrasound. Did you understand the ultrasound part?

Student 3

Not really. I just thought it was fascinating because I could visually see the muscles and nerves. The professor explained which were muscles and organs, but I hadn’t studied ultrasound much, so I mostly just thought, “Wow, that’s amazing.”

Interviewer

About what portion of the total demonstration time was spent on ultrasound?

Student 3

I think about a quarter of the time. During the ultrasound portion, I mostly just watched in awe.

Interviewer

I heard from another interviewee that the professor also came to individual groups and showed ultrasound. Did you feel the same sense of amazement then, or was it different?

Student 3

It felt about the same as when the professor demonstrated to the whole class.

Interviewer

Did the professor upload any ultrasound videos to Google Classroom?

Student 3

Yes, they did. But I don’t think I watched them.

Interviewer

N ow, focusing on the professor’s demonstration of locating points and needling — this was exactly what you were expected to practice during your sessions. Did the demonstration help during your group practice?

Student 3

Yes, it was helpful. During the professor’s demonstration, everyone typed notes on what the professor did, and we referred to those notes when we weren’t sure about something.

Interviewer

Was there a difference between the pre-learning videos and the demonstration?

Student 3

I think there was a difference. One thing I always found a bit disappointing was that the pre-learning videos of acupuncture points seemed a bit older, so there was quite a gap compared to the demonstration.

Interviewer

Since they were recorded quite a while ago.

Student 3

That’s right.

Interviewer

Were there any other differences in content between the pre-learning and the demonstration?

Student 3

I don’t think there were major content differences. But the demonstration was more concise. The pre-lecture focused more on theoretical aspects, and the professor marked each point with a pen in detail during the pre-learning videos. So I think that was a difference.

Interviewer

Did seeing the professor locate points directly help you during your group practice and needling? About how long did the demonstration usually last?

Student 3

It varied depending on which meridian was being shown, but I recall that for the first part, roughly one-third of the time — around 30 minutes — the professor demonstrated continuously.

Interviewer

And what was the maximum demonstration time?

Student 3

I think it was about that range. But the professor didn’t take much time for the demonstration. We actually had more time for our own practice, and the professor went through the demonstrations quickly, so it ended faster than I expected. I don’t remember the exact details.

Interviewer

Is there anything in the demonstration that you wish had been improved or supplemented?

Student 3

I don’t think there was anything that needed improvement. I felt it was sufficient.

Interviewer

Now, moving on to group practice — within the group, roles were divided into physician, observer, and patient. I’ve heard that the practice format varied slightly by group. Could you tell me how practice was conducted in your group?

Student 3

Sure. There were five of us in my group. The professor would first explain what we were going to practice that day, and then we decided who would be the patient, the physician, and the observer. The observer would watch everything and take notes. If there were about 18 acupuncture points to practice, we divided them so that each physician handled 3 to 6 points. One person would explain the details of their assigned points, and the other physicians would palpate and discuss to confirm the locations. After locating the points, the assigned physician would perform the needling.

Interviewer

You mentioned that the observer recorded everything. What exactly did the observer record?

Student 3

They recorded the needle insertion depth, the sensations, and how the patient felt in real time. The physician recorded *deqi* after each insertion. Additionally, the observer noted any shortcomings or points that needed improvement during the physician’s performance.

Interviewer

So the observer was recording all of this in real time during the practice? Did you notice anything about yourself while acting as the physician?

Student 3

Personally, I’m a bit introverted and not very talkative, so when I practiced, I tended to focus only on the needling and didn’t check the patient’s condition closely. But through this practice, I thought it was really important to relax the patient when I am doing acupuncture as a physician, so I thought a lot about the importance of communicating with the patient, so I tried to do a lot more of that.

Interviewer

How did you come to realize that communication with the patient is important?

Student 3

I noticed that most people didn’t have much experience receiving acupuncture, so they were usually very tense before needling. I was the same at first and felt nervous myself. In those situations, I realized that helping the patient relax is really important. I also experienced dizziness lightly, as a patient, which made me feel this even more.

Interviewer

So being a patient yourself helped you recognize that?

Student 3

Yes, definitely. Even when I wasn’t the physician but the patient, I observed other physicians communicating a lot with their patients. For procedures like moxibustion, which take more time, constant communication with the patient was essential. I realized it’s really necessary.

Interviewer

Did you have any other thoughts when you were a patient receiving acupuncture treatment?

Student 3

I really felt grateful whenever someone explained or guided me. At first, I was very tense, so even small needle insertions felt painful. But as I felt more at ease, it didn’t hurt as much, and I could relax. I also think I could sense the physician’s posture, mindset, and confidence. If the physician was nervous, I would feel tense too; if the physician was confident, I could trust them and feel more comfortable. Being a patient really taught me how important the physician’s role is.

Interviewer

You mentioned a confident attitude — did you notice any changes in yourself as a physician because of that?

Student 3

Yes. When we wrote self-reflections in our *practice notes*, I often wrote about that, thinking I needed to improve for next time. I practiced with that in mind, so I did notice some changes. Also, because I became closer with my group members, I felt more comfortable practicing, which may have influenced that feeling. I think next time, in another practice or opportunity, I’ll really be able to see how much I’ve improved compared to the beginning.

Interviewer

How about when you were acting as the observer?

Student 3

As an observer, the patient just stays still, so I focused more on the physician’s posture and the surrounding environment. For example, the needle disposal container was very close to the patient, so if the patient moved even slightly, it could cause a cut or fall. These were things I could notice as an observer that I wouldn’t notice as a physician.

Interviewer

When you noticed issues, like poor hygiene management, how did you act as an observer?

Student 3

I addressed it immediately, though not in a harsh way. Then, when I became the physician next time, I paid much more attention to the surroundings as well.

Interviewer

You mentioned that the observer mainly recorded the *practice notes*. Did you experience any difficulties during that process?

Student 3

Not really. As an observer, you can just focus on recording at the moment, so in that sense, it was actually easier. Physicians or patients often don’t have much time to write while practicing, so they have to fill it out afterward. Observers can record everything immediately, which made it more convenient in my opinion.

Interviewer

Since we’re talking about *practice notes*, I want to ask about the content for each acupuncture point.

Student 3

Okay.

Interviewer

Do you think writing that information is meaningful?

Student 3

Could you clarify which information specifically?

Interviewer

In the form, there’s a section to pre-write the point location process, and then for the practice, you write the needle type used, insertion depth, *deqi* sensation, and the patient’s response. Do you think recording these is meaningful, or do you feel some items are unnecessary?

Student 3

I don’t think any of the items are unnecessary. For me, the *deqi* sensation is very important, which is why recording it is crucial. However, I often didn’t feel the *deqi* much, so when I recorded it, the content sometimes became repetitive, which was a bit difficult. Still, I acknowledge that these are essential items and shouldn’t be removed; it was just challenging for me.

Interviewer

Have you ever looked back at your *practice notes* after writing them?

Student 3

Yes. I usually reviewed the notes a lot before exams. Besides that, I also read my self-reflections before practice, so I looked at them quite often at those times.

Interviewer

So you’re saying you reread the self-reflections you wrote previously?

Student 3

Yes, I read what I had written. Also, many of the parts we found difficult were recorded in the *practice notes*, so when we had extra time for practice, we referred to them a lot to improve our skills.

Interviewer

After the acupuncture training course is fully completed, do you think you’ll look back at your *practice notes* again?

Student 3

Yes, I think I will.

Interviewer

If so, what would you mainly look at?

Student 3

I think I’ll mostly look at the detailed records of the needling process we wrote at the time. Also, any feedback or points the professor highlighted are included, so I’ll refer to those a lot as well.

Interviewer

I heard that toward the end of the training course; you also did an OSCE. The observer scored the performance, and the physician needled the acupuncture points provided by the professor. Is that correct?

Student 3

Could you say that again?

Interviewer

Sure. The observer graded the physician, who performed needling on the acupuncture points given by the professor. As a physician, did you ever experience being evaluated in the OSCE?

Student 3

I mostly acted as the patient, so I never experienced being evaluated in needling. I only did it once during moxibustion. Because there was a time limit and I had to go through all the steps, I realized there were many things I hadn’t practiced, and I learned a lot then.

Interviewer

What do you mean by “things you hadn’t practiced”?

Student 3

During the OSCE evaluation, there were some steps that I didn’t complete, so they were marked as “No” on the checklist.

Interviewer

Do you remember what those were?

Student 3

Generally, we hadn’t been pre-informed about things like “what the patient might feel pain in” or “what to do afterward” in advance. So I got “No” checked mainly on those safety-related aspects. Since I was doing moxibustion and not needling, I’m not exactly sure of all the details.

Interviewer

When you took the moxibustion OSCE, did you feel nervous?

Student 3

Yes, I was very nervous.

Interviewer

Did that nervousness affect your performance, like your score or how you completed the exam?

Student 3

Yes. I’m naturally very timid, overly aware of my surroundings, and when there’s a time limit, I sometimes panic and go blank. So during the exam, it really affected me. I only experienced it once, but it helped me a lot.

Interviewer

You mean the exam. How did it help?

Student 3

Since the OSCE has a set time limit and the professor evaluates each item, experiencing a simulated OSCE before the actual exam was really helpful.

Interviewer

Because the exam formats were similar?

Student 3

Yes.

Interviewer

Since we’re on the topic, how was the exam? Do you think you performed well on locating points and needling within the 5 minutes?

Student 3

I didn’t do well. I barely managed the point location, and I couldn’t really perform needling because the time ran out just as I was disinfecting and about to needle the patient. I feel like I couldn’t fully demonstrate what I had prepared, so I was a bit disappointed.

Interviewer

What made it difficult to show what you had prepared?

Student 3

Mainly because the professor was right in front of me. My whole body trembled, and my speech became awkward. My hands shook while locating the points, so I couldn’t do it properly. Even when I wanted to explain certain content, I forgot it due to the nervousness. I had planned exactly what to say for each point, but I couldn’t express it, which led to some point deductions. That’s why I felt a bit upset.

Interviewer

During the exam, were there any parts you felt confident about?

Student 3

The parts I felt confident about were mainly CNT and how I interacted with the patient. That was my strongest area. Our professor gave us a group assignment, and in our group, we always discussed that “the professor really emphasizes CNT.” So, every time we did a group assignment, we created our own CNT manual and focused on acting according to it. Also, since we recorded videos performing tasks in a doctor–patient format, I felt confident about handling the patient and applying CNT during the exam.

Interviewer

So the group assignments were really helpful?

Student 3

Yes.

Interviewer

On that note, although it’s slightly unrelated to this study, I’m curious—did you face any difficulties while doing the group assignments?

Student 3

At first, it was really overwhelming because it was the first assignment the professor introduced. We didn’t know who to recruit, what to do, and the professor didn’t give us a very detailed manual. The instructions were just points 1 through 7, but they were too abstract, so it was really hard to figure out what to do. Also, the professor didn’t specify how many times we should practice; he just said, “Do it until you get better.” So, we often wondered, “How many times should we do it?” Analyzing the results was also tricky because sometimes things went well and sometimes not, so we had to pay a lot of attention and effort.

Interviewer

During that process, were you able to get help from senior students smoothly?

Student 3

Yes. For things we hadn’t learned, like unfamiliar acupuncture point locations, we got a lot of help. We also received a lot of guidance from senior students in terms of diagnosis since we couldn’t perform the diagnoses ourselves.

Interviewer

You mentioned that the assignment took a lot of time. Do you feel it was worth the effort?

Student 3

Since the assignment was ultimately a form of practice, it helped us gain more hands-on experience. For our group, practicing from the patient’s perspective was really beneficial. I’m not sure about other groups, but for us, it was definitely helpful.

Interviewer

So group assignments' content wasn't shared between groups, only the evaluation results, correct? I see. I think I’ve asked quite a lot of questions already. Is there anything else you would have liked in the class? Any parts you think should be reinforced, or any additional materials that could be helpful?

Student 3

Not really. For acupuncture point location, there are already anatomical references, and with the lecture and PPT, I felt it was enough to study. I was generally satisfied with the training class itself. Nonetheless, I think we were a little more pressed for time because we were discussing and trying to figure out where to put the needle. I think we're running out of time. So I think it would be good to have a bit more time for practice. Other than that, the materials were sufficient for study related to practice.

Interviewer

How much more practice time do you think would be ideal?

Student 3

Honestly, even just a little reduction in the professor’s individual explanations during demonstration might be enough. But if more time were given, maybe 20 minutes more would suffice. Or extra time after the class could also work. One thing we were conscious of was that even if we weren’t done, the group responsible for cleaning had to start, so we felt a bit rushed. It might be okay to let that group handle cleaning even if we finish later.

Interviewer

If we consider the objective of this acupuncture point practice class as *“to be able to safely and hygienically perform needling on the fourteen meridians while considering the patient”*, how much do you think this goal was achieved, on a scale of 0 to 100%?

Student 3

I think it really depends on the individual. For me, I haven’t done many other practical classes, but for this acupuncture training, I tried really hard and prepared well. So, at that time, I’d say I achieved about 90%.

Interviewer

So you think it’s possible for students to reach 100%?

Student 3

I think it could be possible.

Interviewer

I see.

Student 3

But it’s not easy, of course.

Interviewer

If an average student—not exceptionally diligent, but not careless—takes this class, how much of the intended goal do you think they would achieve?

Student 3

I think at least more than half of the goal would be achieved.

Interviewer

Understood. Is there anything else you’d like to add?

Student 3

I don’t think so. Nothing comes to mind.

Interviewer

Okay. I just quickly went through the prepared questions, but you answered in depth, so I hope everything was conveyed properly.

Student 3

Yes, it was conveyed well. Thank you very much. You can finish recording now.

**Interview with Student 4**

Interviewer

Let’s begin the interview. Are you familiar with the manual for the acupuncture training class? What do you know about it?

Student 4

Yes. I know it as the manual that describes how to use the acupuncture training room and the guidelines to follow during class. I learned about it through the materials uploaded to the Google Classroom for the Acupuncture Point and Acupuncture training class.

Interviewer

I see. How did you use those materials during your class?

Student 4

First of all, the acupuncture training course itself was something I was taking for the first time, and it was also my first time entering the training lab. When I first received the manual, I hadn’t been inside the lab yet and didn’t know what the class would be like, so at first, I thought, “What does this mean?” But once I entered the lab and read the manual, I realized it clearly laid out what I needed to do and in what order. Thanks to that, I was able to adapt more easily and feel less lost during the first class. Usually, when entering a training lab for the first time, you don’t know where things are placed or what you’re supposed to do at that moment. But since everything in written form, even though there were many unfamiliar terms at first, I think the manual was quite helpful once I was actually in the training lab.

Interviewer

You mentioned earlier that there were some unfamiliar terms at first. Do you remember any specific terms that were difficult for you?

Student 4

It wasn’t exactly that the terms themselves were complicated, but rather that I didn’t know what the training lab looked like yet. For example, when the manual said “wash your hands,” I didn’t know whether that meant to go to the restroom or if there was a sink inside the practice room. Once I actually entered, I saw that there were hand sanitizers and paper towels provided, so I could understand what the manual meant. Also, there were terms referring to things like the equipment cabinets or the carts we used per group. When I first read the manual, I wasn’t sure what those words meant, but once I saw them in person, I realized, “Ah, this is what I’m supposed to use!”

Interviewer

So before the class started, it was hard to picture the training lab, but once the sessions began, things became clearer?

Student 4

Yes. I’m actually part of the Acupuncture and Moxibustion Study club, so I had been inside the lab once or twice before, but it wasn’t for my own class, and since you need permission to enter, I didn’t really look around or touch anything. So I didn’t know much about the layout. But once it became my class and I knew I had to get used to the space, I made a conscious effort to adapt quickly. I was also serving as a vice-departmental student representative at the time, so my classmates often asked me questions. That made me pay even more attention and really put effort into understanding how everything in the lab was set up.

Interviewer

Then, while getting to know the conditions inside the lab, did you ever think that having additional materials would be helpful?

Student 4

Yes. In other labs, you can figure out where the supplies are located just by looking around, but there were often problems because we didn’t check beforehand. However, in the acupuncture training la b—well, since we only attended the professor’s class, I’ll speak based on that—the professor conducted an orientation during the first class, where we all cleaned the lab together and he demonstrated how to use the training lab. That was really helpful. So, when I first saw the manual, I thought it would be nice if there were a floor plan of the room. But after actually going through that orientation and simulation, I realized it might not be necessary. It’s nice to have written guidance in advance, but once you see and experience it directly, you naturally pick it up.

Interviewer

Then, do you think it’s necessary for the professor to show how to use the equipment during the first class like that?

Student 4

I think it’s definitely a useful session. The professor spends a lot of class time doing that for the students’ convenience, which is something I really appreciate. But if the written manual included a detailed floor plan and clear explanations, then maybe a demonstration wouldn’t be absolutely necessary—though, to be honest, students rarely read written materials carefully. So, I think there’s still a lot of value in experiencing it firsthand. The demonstration itself is great; it’s just that it takes quite a bit of class time.

Interviewer

You said you don’t read written materials much — roughly what percentage of the manual did you read while doing the practice?

Student 4

For me, I read it thoroughly through about week 4, since it was handed out each week. From week 5 on, because there weren’t many changes, the routine was already ingrained, so I only skimmed it to check for updates. In the first week I really studied it carefully, but there were many classmates who didn’t even know the manual existed. Those who read it read carefully, and those who didn’t just didn’t, so we ended up telling each other, “You can just look at it like this

Interviewer

Did you follow the weekly learning manual when you practiced?

Student 4

Yes. The manual shows the professor’s teaching schedule for the week, so we tried to complete the point-location and needling according to that. Usually the weeks are divided by meridian — for example, the Conception Vessel or Spleen Meridian are scheduled across particular weeks. If a topic spanned two weeks, we checked carefully how far the professor wanted us to go and tried to match that. In the first week I reviewed the whole syllabus, but after that it was repetitive, so I mainly checked progress. And, CNT? No that’s not… A test that started after the midterm test, what was it?

Interviewer

OSCE.

Student 4

Yes, OSCE. Also, when there was an OSCE after midterms, I checked the OSCE scope. We also had the weekly plan as a handout and the professor displayed a PPT before class, so we used both to preview the week’s scope.

Interviewer

Then, to ask more specifically about the practice — did you do any pre-learning before each session?

Student 4

Yes, there were pre-learning videos uploaded before class, so I watched those. But those videos mainly focused on the acupuncture procedure, so I felt they didn’t include enough theoretical explanation. So, I also studied the materials uploaded for acupuncture point theory together. Usually, the target structures or cautions for needling were covered more in the theory class, so I actually studied the theoretical materials first, and then watched the pre-learning videos to see how the needling was done in real situations. Also, when conditions allowed — for example, when I went home on weekends or had time with friends — I practiced the parts I was assigned to in advance, thinking, “If I find the point this way, I can locate it faster,” and studied together with them.

Interviewer

Did you practice point location before the class?

Student 4

Yes. We were told that it’s good to try point location on various people, since doing it only on one patient could limit understanding human’s body type. Usually, male students practiced point location on the trunk area, but I practiced with female classmates at home, comparing differences and realizing “It really varies from person to person.” For the arms and legs, we often practiced casually even outside of class.

Interviewer

What materials did you mainly refer to when you practiced point location?

Student 4

I referred to both the materials uploaded in the Acupuncture Point Theory course and the pre-learning videos uploaded for the Acupuncture Point Practice course. When I got confused about the positions of muscles, I also reviewed the anatomy materials I had studied the previous year and used anatomy apps to check muscle locations for reference.

Interviewer

Then, when you actually practiced point location during class, did you have any major difficulties?

Student 4

The most difficult part for me was finding the intercostal spaces. Since there were many areas that female students couldn’t practice on, I mainly practiced on male classmates as patients. But depending on how developed the pectoralis major muscle was, sometimes I couldn’t palpate the space at all, so I really struggled with that. When I practiced on myself, I could feel it and thought, “Oh, it’s palpable,” but on others, when the muscle was firm, I couldn’t find it well. That was the hardest part. Also, in the case of the scapula, its position varied a lot — for some people it was very prominent in the front, and for others, it was positioned much farther back — so I realized that I couldn’t rely just on visual observation, and that I really had to confirm by palpation.

Interviewer

Then, did you have any other difficulties when performing palpation?

Student 4

It wasn’t exactly a difficulty, but more something I felt sorry about. Some group memeber said it hurt when I palpated — especially near the origin or insertion of muscles. When I touched lightly, I couldn’t feel the structure well, but if I pressed deeper, they said it hurt. So it was difficult to find a balance between being gentle and accurately palpating.

Interviewer

When you couldn’t find a point easily during class, what did you do?

Student 4

During the class, the teaching assistants were always present, so whenever I had trouble locating a point or wasn’t sure about the correct needle direction — especially when it wasn’t clearly described in the learning materials — I asked them for help. They would explain, for example, “If you can’t palpate this structure clearly, you can locate the point based on another nearby anatomical landmark,” or sometimes they’d say, “If the stimulation area covers the general target region, it’s okay to use a slightly broader area.” The professor was usually busy supervising other groups, so I mostly asked the TAs. Usually, my group members and I tried to figure things out together first — discussing, “Is this right? Or maybe that?” — and when we couldn’t reach a conclusion, we asked the teaching assistant for guidance.

Interviewer

Then, not just when you couldn’t find a point — but in general, when you were doing point location, did you feel like you could tell whether you were finding the right point or not?

Student 4

Yes. When I could locate and needle a point without confusion, it was usually because the anatomical landmarks were really clear. For example, when I palpated and thought, “This feels exactly like what the professor described,” or when I could feel “the gap between two ligaments on the arm where the professor said the point is,” I felt confident. Also, for the intercostal spaces, when I counted down — first rib, second, third, fourth — and could trace them clearly, it wasn’t too difficult because there were definite landmarks. But for areas like the back or the scapular region, where the spine of the scapula was hard to palpate, or on the thigh where the muscles weren’t easily felt, I felt much less confident. In those cases, I often relied on bone proportional measurements to estimate the location, or I asked the teaching assistant, “Is this the right spot?” And they’d often say, “Not quite — it’s actually here,” and show me the correct location. That’s how I learned through correction.

Interviewer

And during the pre-learning process, you were originally supposed to write down the point location methods in your practice note. Did you actually do that?

Student 4

Honestly, I didn’t write the practice note in advance, but I used both the handbook and my own practice note. For the point I should locating, I made sure to study it in as much detail as possible. For other points that my group member were responsible for, I at least reviewed the professor’s materials before class. During the practice, when someone misplaced a point — like if someone said, “It’s 2 cun,” but another said, “No, it’s 3 cun,” — we’d discuss and correct each other.

So, for point location methods, I referred mainly to the professor’s explanation s and the handbook, then rewrote the steps in my own words so I could understand them better. For example, if the handbook said “fourth intercostal space,” I couldn’t find it right away just from that, so I marked it in advance and planned how to locate it. Or when locating LU2 near the scapula, I’d note, “If I can’t find this, I’ll need to ask for help.” And for LI16, I’d write reminders like, “It’s more anterior than expected, so palpate this muscle first and then move toward that muscle.” I tried to make a plan in my own way — though I wasn’t sure if it was exactly right.

Interviewer

You said you rewrote the point location methods in a way that made sense to you. Did that mean the professor’s explanations or the handbook sometimes weren’t helpful enough?

Student 4

Not exactly. Most of the time, the professor’s explanations matched the handbook, but sometimes they were different. Occasionally, the professor would say, “The standard location is written like this, but for better therapeutic effect, it should be located this way.” In those cases, I organized my notes accordingly. Also, if the handbook’s description of the method was too brief for me to understand, I looked up the surrounding anatomical structures to make sure I could find the point correctly.

Interviewer

I see. Then, at the beginning of the class, according to the learning manual, there’s about 15 minutes allocated for preparation — washing hands and getting materials ready. Did you feel that was enough time?

Student 4

Actually, since there’s only one place to wash hands, it would be impossible for everyone to finish washing within that time if we all started at once. But we all realized that there was only one sink and that time could be tight, so most of us came about five minutes early to wash our hands and get ready. So I think 15 minutes was fine. The students adjusted well on their own. Even without coming too early, just five minutes in advance was enough time for everyone to wash up and prepare.

Interviewer

So, were you also able to get the patient consent forms within that time?

Student 4

Yes, I think 15 minutes was enough to handle the consent forms as well. A few times, there were slight delays — for example, when we couldn’t quickly find the thermometer — but aside from those rare cases, it went smoothly. Since the thermometers and glucometers were limited in number, we had to take turns, so sometimes it took a little longer to locate them. But using the thermometer is quick, so it didn’t really cause any issues.

Interviewer

And did you follow the manual, with the physician checking the patient’s vital signs and obtaining consent forms?

Student 4

Yes. In our group, we tried to have the physician do as much as possible. Since there were multiple physicians, no single person did everything. One measured temperature, another measured blood pressure, another measured blood sugar, and someone handled the consent forms. Each week, four people rotated roles — four physicians, one patient, one observer — so the tasks were divided, with physicians performing the measurements and observers filling out the checklists.

Interviewer

I’d like to ask about obtaining consent — did the physician explain the contents while going through the consent form with the patient?

Student 4

We went through the full explanation in the first week. From the second week onward, everyone was already familiar with the content, so instead of explaining in detail, we mostly just confirmed that the patient understood. We focused more on checking any individual health concerns that day — for example, if someone’s blood pressure was high, especially for older patients, we noted any personal considerations. The rest of the repeated content was just quickly confirmed.

Interviewer

Did you check which areas could be practiced on that day?

Student 4

Yes, for the specific meridian areas — since they extend from the arm to the shoulder and back — we always asked, “Do you consent to exposure in this area?” and got confirmation. In practice, the consent had already been obtained in advance, so in the lab we just reconfirmed, asking “Do you really consent today?” and always made sure to get agreement.

Interviewer

When dividing up the patients within your group, were there any conflicts or difficulties?

Student 4

The male students discussed among themselves and decided that, generally, female students would handle the limbs and shoulders, while the males would take the trunk. So, it was organized in advance, and there weren’t any major conflicts. There were three male students, but I’m not sure if any issues arose among them. For female students practicing on limbs, the male students tended to take on more of the work themselves, so things were managed cooperatively. For example, if the male student was patient during Stomach meridian practice, then for the face or legs, a female student would do it. Overall, I didn’t notice any significant disputes, though I can’t speak for the others’ perspectives.

Interviewer

Besides deciding who would be the patient, were there any difficult situations or conflicts within your group during the semester’s practice sessions?

Student 4

Not particularly. Everyone just had an attitude like, “Good job,” and things went smoothly. Maybe it’s because many of the students were older, but the atmosphere was always polite. When we uploaded observation photos or other materials, everyone thanked each other, so there wasn’t any criticism or tension. Overall, it ended peacefully.

Interviewer

During the professor’s demonstration sessions, it seemed there were two main parts: point location and needling, and then the ultrasound demonstration. Were the point location and needling demonstrations helpful for your practice?

Student 4

Yes, I think they were very helpful. The pre-learning videos we used for learning were filmed quite a while ago, so sometimes they didn’t fully reflect the professor’s current teaching focus. But during the demonstration sessions, we could clearly see what the professor emphasized. He also explained which type of needle to use and verbally described the target anatomical structures for needling. So we could confirm whether what we had studied beforehand was accurate, and we could observe exactly how to perform the needling in practice. That was really helpful.

Interviewer

The demonstration screen is transmitted through the GoPro, right? Were you able to see it clearly?

Student 4

Yes, the screen was clear, but the professor was carrying it around like a magic wand—it sometimes looked a bit cumbersome. It often seemed like the camera wasn’t capturing exactly what the professor wanted, so he appeared uncomfortable. From our perspective, we could see well because the professor tried hard to adjust the angle for us, but setting it up seemed to take him a long time. It looked inconvenient—he’d be doing something, then have to move the GoPro again because it got in the way. I thought it would be nice if there were a way to record from a first-person view, but since that wasn’t possible, it looked somewhat uncomfortable for him.

Interviewer

During the ultrasound demonstration, were you able to understand what you were seeing on the screen? Could you tell which anatomical structures the ultrasound image was showing?

Student 4

Just by looking at the screen, honestly, it was a bit hard to tell. I could guess—like, “That must be a blood vessel because it’s pulsating,” or “That must be a muscle.” But I couldn’t tell exactly which muscle or which blood vessel it was. When the professor explained along with the image, then I could understand things like, “Oh, that’s a nerve—it’s the median nerve,” or “That’s a structure we should avoid,” or “That’s the target muscle.” But if I were just looking at the screen without explanation, I don’t think I would have known.

Interviewer

During class, the professor sometimes came to your group and showed the ultrasound directly, right? How was that experience? Was it different from watching the demonstration in front of the class?

Student 4

During the main demonstration, we watched from a distance, so depending on how the ultrasound handle—what do you call it, the handle?—was positioned, the image would look different, but we couldn’t see exactly how the professor was holding it. When he came to our group and showed it up close, I could see, “Oh, when it’s held like this, the image looks like that.” After that, I could sort of imagine the handle’s position when watching the screen, which was helpful. Since it was my first time seeing ultrasound images in an acupuncture training class, it had felt unfamiliar, but seeing it up close made it feel more familiar. Other than that, there wasn’t a big difference.

Interviewer

Have you watched the ultrasound learning videos uploaded on Google Classroom?

Student 4

I did see that they were uploaded, but I didn’t actually watch them.

Interviewer

Was there a reason you didn’t check them?

Student 4

Honestly, I didn’t have enough capacity to study the ultrasound materials as well. There were already a lot of acupuncture training pre-learning videos and practice materials.

Interviewer

Was the learning workload heavy for you?

Student 4

Yes. Some of my classmates said, “Even if you watch it, you can’t really understand it,” so I guess I felt like I might as well give up on that part.

Interviewer

So your classmates also said they couldn’t really understand the videos, right?

Student 4

Right, right. The ultrasound videos were uploaded, but I think they were posted a bit later—unlike the other videos that were uploaded before the semester started, the ultrasound ones were uploaded after classes began. So quite a few people watched them, but everyone was like, “I can tell that’s a muscle, but I don’t know which one.” So we said, “Then you and I are the same,” and I thought, “Well, I won’t bother watching either.” (laughs) It’s kind of embarrassing to admit that.

Interviewer

How long did the demonstration sessions usually last?

Student 4

I remember they were sometimes longer than scheduled. The sessions where we also watched the ultrasound videos were a bit longer, and the ones with only demonstration were shorter—it varied from week to week. The longest one I remember lasted about 30 minutes, and that felt quite long. The others were around 20 minutes, which just felt normal. But when it went over 30 minutes, I remember thinking, “Oh, today’s session is a bit long.”

Interviewer

Then, do you remember around what time the practice sessions usually started?

Student 4

Usually, I think we started around 40 or 50 minutes past the hour. But on days when things took longer, I remember thinking, “Oh, it’s already almost on the hour.” That’s probably why it stuck in my mind. Actually, though, I didn’t really check the clock much in the lab. There was a schedule in the manual, but I just kind of went with the flow—like, “It ends when it ends.” I think it’s also because I’m just not someone who checks the time rarely.

Interviewer

I see. Then do you think the group practice time was sufficient?

Student 4

Most of the time, it was fine—we could finish within the allotted time. But there were occasionally days when we had a lot to do, and on those days, we kind of came prepared knowing it would run late. The class was scheduled to end at 7 p.m., but sometimes it went to about 7:30. Still, that was manageable. You can’t really expect to finish at the same time when you have to do 10 points one day and 30 another. Overall, the time was generally sufficient, and on the busier days, we just accepted it and worked through. Even then, 7:30 was about the latest it went.

Interviewer

Then, during point location practice, was one person mainly in charge of a specific acupuncture point and also responsible for needling it?

Student 4

In our group, for example, if we had 20 points to locate that day, we’d divide them up among the four physicians—so each person would take about five points. I’d do five, the next person would do five, and so on. Having one person handle all the points would’ve been too much to study at once, and dividing them helped us learn together and stay focused. It also felt like we could observe and check each other’s work more carefully that way. So rather than one person doing all 20, we each did five points.

Interviewer

I see. Then did the person in charge of a point mainly take the lead while others observed?

Student 4

Yes, others would observe and also check how the patient felt—like whether there were any abnormal reactions. For example, they’d point out if someone was crossing the sterile field. So the observers were actively watching too. The main physicians would also give each other feedback, like “Your point location seems off,” or “Try angling the needle that way—it might go in better.” Sometimes we’d notice someone was needling too close to the trunk and say, “You should go more outward.” We also discussed things like, “This area seems sensitive—maybe use a thinner needle here.” So in that sense, the observers were a bit more detached, but the physicians actively helped and corrected each other.

Interviewer

Then what kind of tasks did the observers mainly do during class?

Student 4

The observers mostly focused on CNT-related aspects, and also checked whether communication between the physician and the patient was going smoothly—for example, whether the physician was properly obtaining consent before point location or needling. So observers mainly monitored those parts, while the other physicians next to the needler checked the needling technique itself. But the observers were usually the ones who did the record-keeping. If there was a serious mistake in point location or something looked off, the observer would note it down in the practice note while writing their own observations.

Interviewer

So, did the physician and observer fill out the practice note together?

Student 4

Yes. The patient wrote about how they felt during needling, and the observer filled in items like whether the procedure was appropriate, whether communication was proper, and so on. For each physician, the observer wrote their name and detailed comments like “During point X, physician A communicated appropriately” or “During point Y, something was lacking.” The physicians wrote about their assigned acupuncture points—such as pre-study notes, needle specifications, and even the sensation of needling (deqi). The patient wrote only about how the needling felt, and at the top section—there was one more item to fill in, I can’t quite remember what it was—but that part too. The observer also recorded vital signs like body temperature. Every week, all group members had to sign their practice note.

Interviewer

Since the patient can’t write during class, did they fill it out afterward?

Student 4

Yes. During practice, the physician would ask the patient how they felt during needling, and we’d either film or record audio so we could keep that as a reference. Then the patient filled in their part later, using those recordings. Since we sometimes did around 40 points in one day, many people couldn’t remember details afterward, so we tried to leave as much record as possible to help with writing later.

Interviewer

Then, while writing the practice note, did you find anything difficult?

Student 4

It wasn’t exactly difficult, but for the section labeled “pre-study content,” I often didn’t know what to write, so it felt a bit superficial. I wasn’t sure if I should write what I personally studied, or if I should write information about the acupuncture point itself. Of course, both are probably needed, but I didn’t really have the energy for that. So I just wrote down the most basic things there, and the things I actually studied, I just scribbled in my own notebook. That’s why writing the “pre-study content” part was a bit of a concern for me—I wanted to write it better, but it felt ambiguous.

Interviewer

I see. Then, have you ever seen an example file of a practice note?

Student 4

I don’t think I saw a real example file. I think there was just an example shown once, but it only had very formal, rigid content. So, when I tried to follow that, I wondered, “If this is what I’m supposed to write, what’s the point of writing it?” I mean, I wished I could write more about what I actually studied, but the example was so stiffly written that I just ended up writing it that way. Looking back now, I wish I could have written more of my own study notes instead.

Interviewer

I see. While performing as the physician during the class, did you have any particular reflection or realizations?

Student 4

In what sense do you mean?

Interviewer

For example, things like “I should study this more,” or “I need to be careful about that,” or “I realized this is important for the future”—anything like that.

Student 4

I mentioned this earlier, but during the pre-study, I realized that I really need to practice on a variety of people. When I palpated my father, mother, and younger sibling, each of them felt completely different. I usually practiced on myself the most, but the feeling of touching myself and touching someone else was totally different. And even among different people, everyone feels distinct. So, I realized that just because I could locate a point on one person doesn’t mean I can find it the same way on another. I thought I should prepare multiple approaches—Plan A, B, C, and D—so that if one method doesn’t work, I can try another. Also, when it came to needling, I felt that the sensation of inserting a needle into myself and into another person was completely different. It was actually quite scary at first to needle someone else. But I realized that fear comes from ignorance. Once I fully understand what structures are located in a particular area, that fear will go away. So, I felt that I really need to study the surrounding anatomical structures much more thoroughly.

Interviewer

After realizing that, did you study more about the surrounding anatomical structures?

Student 4

I couldn’t study all the acupuncture points, but for the areas I was in charge of that week, I used an anatomy app to look closely at the surrounding structures. There were many structures that weren’t clearly described, so I just kept looking at that specific area and thought, “Oh, there’s a nerve here. There’s a blood vessel here. But I still don’t really know where exactly they are.” I would realize, “I don’t know enough,” but still kept studying. So, I tried to needle more carefully. Even when the professor said, “You can insert it this deep,” I couldn’t do it because I was scared. I just couldn’t see inside. But I think I started to understand a bit of what it means to “know by touch.” The professor said, “If you palpate like this, you can feel what’s underneath.” I’m not at that level yet, but I could tell that the feeling changes depending on where I press. So I would think, “Then what’s located here?” and look it up. That’s about as far as I got—I’m not sure if that’s the right way, though.

Interviewer

There was a section in the practice note for self-reflection of your learning. Do you remember what kinds of things you usually wrote there?

Student 4

I usually wrote about reflections or things I newly realized. It was often something like, “Today, too, things were different from what I expected.”

Interviewer

In what way were they different from what you expected?

Student 4

Like I mentioned earlier about palpating the ribs—I thought, “If I touch it, I should be able to feel it,” but I realized, “Even if I touch, sometimes I can’t.” And for acupuncture points located between the ribs, the textbook says they “target the intercostal space,” but I learned that in modern practice, they’re not actually used that way. Or I’d write things like, “The professor explained that since the textbook says it’s for skin disorders, we can treat it with shallow needling.” Or, “When I couldn’t find a certain acupuncture point during point location, I asked for help, and the professor told me how to locate it.” I wrote things like that.

Interviewer

Did you write down the feedback you received?

Student 4

Yes. There wasn’t a designated space for feedback, so I often wrote it in the reflection section. I also noted things I felt during the point location process, like “the muscle is really hard,” stuff like that.

Interviewer

You mentioned writing reflections. What kind of things did you reflect on?

Student 4

The professor often says, “If you insert the needle quickly, it won’t hurt.” But I was always scared, so I inserted slowly. Then the patient would feel pain, and I’d think, “Next time, I should do it faster.” But the next week, I’d still go slowly. If I had five points to do, the first one would be slow, then the second a bit faster, and by the fourth, a bit quicker. So I kept reflecting, “I need to do it faster,” and noting things like, “I keep doing this even though I’ve been told,” or just writing, “I was scared.”

Interviewer

Even as the practice continued, did you still feel scared?

Student 4

As time pass, I became calm, and I got better over time. But the first time in the training lab, even when I consciously thought, “I need to insert quickly,” my hands still moved slowly. It’s like knowing what to do but being unable to act—like trying to exercise without warming up. I reflected on that every time.

Interviewer

When you took the acupuncture practice exam, which parts did you feel confident about?

Student 4

Well… the parts I felt confident about… I really wasn’t confident about needling itself. I was okay with point location and explaining how I located the point—like describing the reference landmarks I used—but for needling, there are many additional considerations. With point location, I only needed to know the surrounding structures and the method, but for needling, I had to consider the direction, depth, needle thickness, and other factors. I hadn’t fully mastered all that, so I wasn’t confident—just for locating the point and explaining it.

Interviewer

What kind of practice do you think would help you gain more confidence in needling?

Student 4

In needling.

Interviewer

Yes.

Student 4

I gained confidence in point location and explaining it because we practiced together repeatedly. But for needling, I couldn’t really practice on others—it wouldn’t be appropriate to keep inserting needles into other people—so I didn’t get much opportunity to practice. I did practice on my own body, but there weren’t enough chances to practice on others. I think to gain confidence, practice is the only way. Even with sufficient pre-study, you still need a lot of hands-on experience.

Interviewer

So, your lack of confidence in needling—was that mainly because there was so much to memorize, or because actually inserting a needle into someone felt intimidating?

Student 4

Inserting a needle into someone wasn’t scary in itself. It’s just that I didn’t feel fully prepared. I felt a bit guilty thinking, “I’m inserting a needle into someone while I’m still inexperienced.” I studied, of course, but I still didn’t feel very competent. During class, for the 5–7 points I was responsible for, I studied the most among my peers, so I could manage those. But if someone asked me to needle a random person on the spot, I’d say, “I can’t do that.”

Interviewer

If the educational objective of this acupuncture practice is “to be able to safely and hygienically needle the learned points on the meridians,” how much do you feel the acupuncture training course achieved that goal, on a scale of 0 to 100%?

Student 4

About 70%. I still feel a lot of pressure with actual needling. But for safety and hygiene, we’ve done so much in terms of safety and hygiene that I would say that’s really the whole thing. Needling itself, though, still has a long way to go, so I’d rate it around 70%.

Interviewer

So, if there were more opportunities to practice needling during the class, do you think that would help improve this?

Student 4

Yes. I’ve heard that everyone practices on their own at home. But during this semester, with other professors’ courses, we also have to do portfolios, so we end up practicing at home autonomously. The problem is, if something goes wrong, there’s no one to guide us, so we have to be very cautious. In the practice lab, though, the professor and assistants are present. So if we could practice needling more under that kind of supervision, I think it would really help.

Interviewer

Do you have any suggestions or things you think could be improved in the class?

Student 4

Some of the pre-learning videos uploaded for learning are a bit outdated, so they don’t fully match what the professor emphasizes nowadays. When there’s a discrepancy, it can be confusing. It would be helpful if those videos were updated, or if at least the videos recorded during our current class demonstrations could be used. Also, during the professor’s demonstrations, handling the camera seems cumbersome. If there were a way to mount it on the head or chest, it might make things easier.

Regarding the acupuncture training lab, the lack of desks can be inconvenient, though adding them might feel awkward in the lab setup. In terms of clean zones, sometimes there’s confusion between where to place the used needles versus the needles in use. About the clean zones—the sponge for used needles and the one for discarded needles were right next to each other, which got confusing sometimes. Those should be more clearly separated. And even though CNT aims for sterile conditions, we weren't really maintaining that. Plus, the practice supply boxes were really dirty, so those things could definitely be improved.

Interviewer

Are you referring to the baskets where the materials are gathered?

Student 4

Yes. For example, after washing and disinfecting our hands, we’re not always sure if the needle packets are in a clean zone. When we use the sponge to hold needles during practice, we sometimes wonder if it’s really clean, because it’s not replaced each time. We also ask ourselves whether it’s okay to store the needle packets together with the sponge. So, I think improving these aspects would be helpful.

Interviewer

Thank you. You’ve done a great job today.

**Interview with Student 5**

Interviewer

First, may I ask how the atmosphere was in your group during the *acupuncture point* practice class? I know I shared the interview questions with you beforehand, but I might ask them a little differently as we go.

Student 5

In our group, there were many people who worked really hard. We had a assignment, and everyone participated enthusiastically. One of our group members actually suffered from insomnia and dyspepsia, which we thought were quite common conditions among modern people. Since musculoskeletal disorders are often treated at Korean medicine clinics, we thought that if we were to deal with something other than those, it would be conditions like these. So, everyone was very committed to the project. However, since musculoskeletal disorders can be measured with more objective indicators like ROM, while insomnia relies only on what the patient says, we wanted to make the evaluation a bit more objective. So we worked hard on designing questionnaires and such. Overall, it was a very diligent group, and everyone actively participated during the practice sessions as well.

Interviewer

You must have worked really hard. Then, I’d like to ask you a bit about the usual content covered in your *acupuncture training* classes. First, are you familiar with the manual that was used during the *class*?

Student 5

When you say “manual,” do you mean the process where we first come in, prepare, listen to the professor’s lecture, and then start practicing? Or do you mean something else…

Interviewer

So, it seems like nothing specific comes to mind when you think of the manual, right?

Student 5

Well, yes. Do you mean things like the *lab* manual or consent forms from the *patient*, then writing the practice consent form, and so on?

Interviewer

Okay, great. Actually, what you just said already covers most of what I wanted to ask. But let me check — have you ever looked at the equipment instruction files that the professor uploaded on Google Drive or Google Classroom?

Student 5

Yes, I have.

Interviewer

Then, let me ask about that in more detail. Did those manuals help you during the acupuncture training? Or did you actually use them while practicing?

Student 5

I did use them, but… well, on days when I checked Google Classroom in advance before coming to the *lab*, I remembered the content better. But by the next session, I’d kind of forget again. So I think I should’ve reviewed them more regularly.

Interviewer

And during each class, the professor either displayed or handed out a printed schedule showing how the class would proceed. Did you happen to refer to that during your acupuncture training?

Student 5

Yes, but only one copy was usually handed out, so instead of looking at the paper itself, I mostly checked the version uploaded on Google Classroom. Since we all had to share the printed copy, I reviewed the online one individually before class.

Interviewer

Ah, so you reviewed that content before the practice?

Student 5

Yes, yes — pretty much right before the practice, when I arrived at the *lab* and was getting ready.

Interviewer

I see. Then, did you find that information helpful while you were practicing? Or — let me ask more specifically — what was it like referring to that material during the session?

Student 5

It helped me see how far we had progressed in that day’s class. It also listed the *acupuncture points* or *extra points* we needed to locate and practice, so during the session I could check which ones we had already covered and which ones were still left.

Interviewer

I see. So there was a schedule laid out for each session — did the class usually follow that schedule as planned?

Student 5

The demosnstration part tended to run a bit long, so I think we sometimes didn’t have enough time for practice. The professor explained a lot of things in detail, but since there were quite a lot of *acupuncture points* we had to practice as second-year students, we often felt a bit rushed compared to what was written in the schedule. Sometimes the practice time even went beyond the planned hours, so overall it felt like things moved a little hurriedly.

Interviewer

Then, roughly how long do you remember the professor’s demonstration part lasting?

Student 5

I think it was usually about an hour and a half.

Interviewer

And when the professor gave those lectures, how did he usually conduct them?

Student 5

He usually started the class with a short meditation, then introduced which body area we’d be working on that day. After that, he demonstrated *point location* directly, showing us the process, and toward the end, he often used the ultrasound to show and explain the anatomical structures.

Interviewer

Then, did the professor demonstrate *point location* and needling during class?

Student 5

He didn’t demonstrate *point location* that much. As for needling, he usually showed one or two points during class. He often focused more on areas like Jianjing, or other higher-risk points. So rather than performing a lot of needling, he usually demonstrated *point location* using Betadine swabs and explained the surrounding anatomical structures. That was mostly how he did it.

Interviewer

I see. Then, were those *point location* demonstrations helpful later when your group practiced locating points on your own?

Student 5

Yes, they were really helpful. The *Handbook of Standard Acupoints* lists where each point is located, but it doesn’t explain much about *how* to actually find it. So during our practice, I relied a lot on what the professor had said during class, as well as the demonstration videos he uploaded to Google Classroom.

Interviewer

Between the pre-learning videos uploaded on Google Classroom and the live demonstrations the professor did in front of the class, which one do you think was more helpful for you?

Student 5

I think the pre-learning videos were a bit more helpful. The videos were shorter than the in-class demonstrations, so it was easier to stay focused. And during class, when the professor carried the camera around while demonstrating, the angle sometimes wasn’t quite right, so it was hard to see clearly. When I was close to the front, I could see the professor directly instead of through the camera, but even then, I found that watching the video helped me concentrate better — it was easier to see everything in detail.

Interviewer

I see. You mentioned earlier that the professor didn’t demonstrate needling very often, but when he did show it, did that help you understand how to perform the needling yourself?

Student 5

Yes, it definitely helped to some extent. By watching, I could get a sense of how deep the needle should go. That was really helpful for getting a feel for it — you shouldn’t insert it too deeply, but also not too shallowly. So seeing how deeply the professor inserted the needle was quite important for understanding the right balance.

Interviewer

I see. So, for the needling procedure, there are several steps — from hand washing, opening the needle, holding it, inserting, withdrawing, and disinfecting. Among all those steps, when you watched the professor’s demonstrations, did you pay the most attention to the depth or position of the needle insertion?

Student 5

Yes, that’s what I focused on the most.

Interviewer

The depth of insertion, right?

Student 5

Yes, yes. I’ve always felt a bit unsure about how deep to insert the needle, so I paid a lot of attention to that part during class.

Interviewer

What about the needling location? Did you also feel less confident about that?

Student 5

The location itself — I was fairly confident in *point location*, so that part was okay. But I often worried about inserting the needle in risky areas, so I found the professor’s explanations and demonstrations on those parts really helpful.

Interviewer

But could you actually see how deep the professor inserted the needle through the camera?

Student 5

Only roughly — I could get a general idea.

Interviewer

Then what about the pre-learning videos uploaded on Google Classroom? Could you see the needling depth clearly in those?

Student 5

Not really. The videos were a bit dark, so it was hard to see clearly.

Interviewer

If those pre-learning videos on Google Classroom were to be re-recorded, how do you think they should be filmed?

Student 5

Well…

Interviewer

So they’d be easier to see?

Student 5

Yes, it would be nice if the videos were brighter. They were pretty dark, so while the *point location* part was clear, the needling wasn’t very visible — since the needles are so thin. The videos were really good for watching the *point location*, though.

Interviewer

So you mean the *point location* part was easy to see?

Student 5

Yes, because the professor used a pen to draw on the patient and brought model while explaining, so that part was clear.

Interviewer

Other than making the videos brighter, is there anything else you’d like to see added or improved?

Student 5

Overall, I think they’re fine. But if I remember correctly, I don’t think there was any content related to ultrasound — or at least, I don’t recall seeing it. I think that part might’ve been missing.

Interviewer

Do you mean there wasn’t any footage showing the professor actually using the ultrasound on a student?

Student 5

Yes, that’s right. As far as I remember, there wasn’t. I think it would be nice to include that.

Interviewer

So you mean it would be helpful if the video showed how the professor scans with the ultrasound?

Student 5

Yes, exactly.

Interviewer

I see. I heard that the theoretical content about ultrasound was uploaded to Google Classroom. Have you studied that material?

Student 5

No, I haven’t.

Interviewer

May I ask why?

Student 5

Honestly, there was just too much to study already. And since the professor covered a lot of the ultrasound material during class anyway, I didn’t really have the time or energy to go through it again on my own.

Interviewer

Then, when the professor showed the ultrasound images during practice, were you able to understand what the video was showing — like which anatomical structures were being displayed?

Student 5

At first, honestly, all I could see were black and white shapes. But as I listened to the professor’s explanations, I started to understand a bit — like, “Oh, that’s a blood vessel,” or “If it’s pulsing, it must be a vessel,” or “There’s something located here.” So I could roughly follow along, but I still can’t interpret the images on my own yet. I can follow the explanation when the professor describes it, but without that, it’s still difficult.

Interviewer

So, it’s still difficult for you to look at the position of the ultrasound probe and identify the structures on the screen by yourself, right?

Student 5

Yes, that’s still quite hard for me.

Interviewer

So, when it comes to ultrasound, sometimes you all watched together with the screen split like that, and other times, when each group wass doing the practice, the professor came by and showed you individually. Between those two, which way did you feel helped you understand better?

Student 5

I think it was better when the professor came over and showed us directly. You could see whether they held the probe horizontally or vertically, and exactly where on the patient’s body they placed it. So, I guess that made it a bit easier to understand.

Interviewer

So, if we were recording ultrasound demonstration videos, like you mentioned, and uploading them to Google Classroom, what kind of content do you think would be most helpful? For example, showing the needle depth through the structure, explaining what happens with the anatomical structures, or other types of explanations—what do you think would help the most during practice?

Student 5

I think explaining the anatomical structures would be the most important. Also, a bit of explanation about things like blood vessels, nerves, and muscles would be really helpful.

Interviewer

So, you can actually see anatomical structures by illustrations in the handbook or other books, right? Even with that, does seeing them through ultrasound feel a bit different to you?

Student 5

Yeah, seeing it on ultrasound feels really unfamiliar. It’s hard to immediately recognize what I’m looking at. With the books, I’m used to them, so I can tell what’s what. But with ultrasound, even after looking, it’s kind of tricky to figure out. For blood vessels, I can tell—they’re just moving, so I know that’s a vessel. But for muscles and stuff, you have to apply what you already know to what you’re seeing there. So, it’s a bit harder to piece it all together. I think it would be helpful if that part was reinforced a bit more.

Interviewer

So, let’s move on to the group practice, with the roles of physician, observer, and patient, did you get to experience all three?

Student 5

Yes, I did.

Interviewer

And when you first acted as the physician, were there any points you found difficult?

Student 5

Yeah. At first, when I was the physician, I felt really nervous about whether I was doing it correctly. I was extra careful because I didn’t want to make a mistake. I disinfected really thoroughly, checked multiple times if I was doing it right, and kept making sure I was inserting the needle at the right depth. So at the beginning, being the physician took a lot of time. But now, it’s a bit faster—much more manageable.

Interviewer

So, you mentioned disinfecting, locating points, and checking the needle depth—how did you actually do that?

Student 5

Well, when I was locating points, I got a lot of help from my group members. They’d check if I was doing it correctly, and I also compared the muscles and tendons with the handbook to make sure I was on the right point before inserting the needle.

Interviewer

So you checked the handbook before inserting the needle. And how did you check the needle depth?

Student 5

At first, I mostly went very shallow because I was a bit scared to go deeper. But as time went on, especially with the arms and legs, I realized I could go a bit deeper. I learned by watching how others did it and listening to some advice, so gradually I got more comfortable.

Interviewer

Through your group members, then.

Student 5

Yeah.

Interviewer

So when you were practicing with your group, did you usually study a bit beforehand?

Student 5

You mean, did we study together?

Interviewer

No, I mean just generally—did members tend to study on their own before practice?

Student 5

Some did, some didn’t. But this semester, since we have to write the practice notes, we have to watch the videos before class, so I think most people ended up studying beforehand.

Interviewer

You mean the theory lectures, not the acupuncture training videos or practice notes, right?

Student 5

Yes.

Interviewer

Since you have to write the practice notes and watch the videos beforehand, did you already have some knowledge about the acupuncture points?

Student 5

I mean, generally I had a rough idea of where the points are, so I guess that helped.

Interviewer

And what about the pre-learning videos on Google Classroom that you’re supposed to watch before class—how much do you think your group actually watched?

Student 5

I’d say about half of it.

Interviewer

So, for the pre-learning videos you studied beforehand, did you organize the content anywhere separately?

Student 5

Yeah, for the parts I thought I needed to remember during practice, I jotted them down a bit in the handbook.

Interviewer

So you didn’t write them in the practice notes in advance?

Student 5

Actually, I did write some of the pre-study content in the practice notes too. I’d look at that while practicing.

Interviewer

Was it helpful to refer to that while practicing?

Student 5

Yeah, it helped a bit. Since the videos don’t really explain how to locate points in detail and just show the positions, having that note was useful.

Interviewer

So you mainly organized it around the point locating methods?

Student 5

Yes, exactly.

Interviewer

When you were the patient, were there any parts that were difficult or challenging?

Student 5

At first, I was really scared. So I was super sensitive to any pressure—I’d feel a lot of pain and stuff. But mostly, as I got used to it and my group members got more skilled, being the patient became much easier.

Interviewer

When you say you were scared at first, was it mainly worrying about any possible harm from the needles?

Student 5

Partly, but I think it was mostly just being a little scared of the needles themselves. That was the biggest thing.

Interviewer

So, on a typical training class, how many needles would you get?

Student 5

Well, we had to practice all the acupuncture points, so I got all of those. And even for the extra points in the meridian system, we practiced those too. Plus, if a mistake happened—like the needle didn’t stay in properly—we had to redo it. So all in all, I think I got more than 30 needles in a day.

Interviewer

More than 30 in one day?

Student 5

Yeah, because some points were done multiple times.

Interviewer

And usually, was it one physician per acupuncture point, or did several people work on the same point?

Student 5

Usually, it was just one physician per point.

Interviewer

While being a patient, did you notice anything else?

Student 5

Yeah, I think I realized a lot about what a physician can do to make the patient more comfortable. If the physician seems scared of inserting the needle, it actually makes the patient more nervous. So it’s important to show confidence and professionalism. Also, seeing the physician wash their hands properly, disinfect well, and follow proper procedures gives the patient a sense of security. I noticed that was really helpful.

Interviewer

And the next time you were the physician, did you remember those points?

Student 5

Yes. When I had only been the physician before, I didn’t really notice. But after being the patient once, I could understand those things much better.

Interviewer

And when you were the observer, did you notice anything in particular?

Student 5

Yeah, as an observer, I could watch both the physician and the patient, so I got a much clearer view of how the physician was working. When I was the physician, I focused on how I was doing, so I didn’t really notice. And when I was the patient, I focused on receiving the needle, so I couldn’t really see either. But as an observer, I could see things like, “The physician missed a step here” or “They were a bit sloppy with locating points there.” That made it easier to give feedback to my group members and also helped me improve myself later.

Interviewer

And as an observer, did you notice anything that should have been done properly but wasn’t?

Student 5

Yeah. For example, after inserting and removing the needle, sometimes people didn’t press with a cotton ball to prevent minor bleeding. Also, things like disinfecting again or applying a bandage were often skipped. During the actual needle insertion, there weren’t many big mistakes, but after insertion and removal, those steps were often neglected.

Also, when locating points, sometimes people would just measure a few “cun” and stop, without really checking if it was the correct indentation point. They mostly relied on the measurement itself, using methods like “proportional bone cun” or “finger width mesurement” so I felt that part was a bit lacking.

Interviewer

So what do you think is needed to improve those parts?

Student 5

I think it helps a lot when your group members keep pointing out what you’re doing wrong, because you often don’t notice it yourself. Whether it’s the observer, or another physician in the group, hearing it a few times really helps you correct it. That was pretty useful.

Interviewer

And did your group ever get stuck together, not knowing what to do?

Student 5

Yeah, when locating points on the back, like figuring out which thoracic vertebra it was, we sometimes got a bit confused. In those cases, we all got stuck, and the professor had to help us. Yeah, that happened.

Interviewer

So how did you usually handle writing the practice notes? Who was responsible for what?

Student 5

Well, first we divided up the pre-study content. There were six of us, so we split it and each wrote our part. After practice, the physician would write about the acupuncture points they needled, the patient would write about their experience, and the observer would write their observations. Each person wrote about their assigned role. For example, the observer’s notes went in the section before the practice description, and the patient’s notes were included in their section. Basically, everyone just wrote about the role they took.

Interviewer

And when did you usually write the practice notes?

Student 5

I think the deadline was Sunday, but we usually finished writing everything by Saturday.

Interviewer

So during practice, what kind of content did you usually write down?

Student 5

The needle depth, for one. And since the patient’s feelings might be hard to remember later, the observer would record that. Also, things like how it felt from the physician’s side—the observer would jot down just enough so you could remember roughly later.

Interviewer

So during practice, the observer mainly handled the recording?

Student 5

Yeah, that’s how it was during class.

Interviewer

And to write that kind of content, did the observer have enough opportunity to watch what the others were doing?

Student 5

Yeah, the observer had to watch carefully to be able to write it down, so I think I paid more attention when I was the observer.

Interviewer

So, I heard that you started doing the OSCE around the time the midterm ended. Did you get to experience that?

Student 5

Yes, I did.

Interviewer

And were you taking the exam, or were you one of the graders?

Student 5

I was taking the exam. Yeah, the one taking the exam.

Interviewer

Right, so you were the one who had to take the exam. Do you think having the OSCE made any difference in the way you approached the practice itself?

Student 5

Yeah, because there were clear tasks that had to be done within about five minutes, it tested whether I could focus and get everything done efficiently. And since there was someone grading me, I was a bit more nervous. During regular practice, I don’t think I focused as much. Honestly, the OSCE was a good experience to practice more intensively.

Interviewer

So comparing practice before the OSCE was introduced and after, did you notice any difference?

Student 5

Well, the OSCE has clear standards, so it made it easier to know which points I really needed to pay attention to. Even during training class, I started thinking more carefully about what I should focus on.

Interviewer

And during the OSCE, how was the nervousness? Were you really nervous?

Student 5

Not overly nervous, but I was a bit more tense since I had to finish everything within the time limit and there was someone watching and grading me.

Interviewer

But you wouldn’t say you were very nervous?

Student 5

No.

Interviewer

So I understand you took a exam at the end of the semester. How was the tension or nervousness during that exam?

Student 5

We had to locate five acupuncture points and insert a needle into one of them. I felt the time was really tight, so I was more nervous. It took me a bit too long just to locate the points, and sometimes the time ran out before I even got to needling. So the time pressure made me more tense. For the OSCE, I think we only had to handle two or three points, so the pressure wasn’t as intense there.

Interviewer

During the exam, were there parts you felt confident about?

Student 5

Yeah, for example, the face area—the points are really clear, so I felt confident there. I was also somewhat confident with the limbs, but from the neck down to the back, I wasn’t really confident.

Interviewer

You mean for locating the points?

Student 5

Yes, for example finding C7—I wasn’t really sure if I was doing it correctly.

Interviewer

Aside from locating points, was there anything else you felt confident about?

Student 5

Yeah, I was confident about needling too, but I didn’t get to do it because of the time limit.

Interviewer

Were there any parts you found difficult during the exam?

Student 5

First, before the exam starts, we get about a minute to plan the order of the acupuncture points we’re going to locate, and it was a bit tricky to decide that quickly and communicate it well. And during the point location itself, it was a little hard to be confident that I was doing it correctly.

Interviewer

You mentioned you didn’t have enough time just for point location. Why do you think that was?

Student 5

As I said, it was hard to feel sure about each point, so I ended up checking the same point several times. That took up a lot of time. If I had just moved on more quickly, I probably would’ve had time left for needling, but since I kept double-checking, I ran out of time.

Interviewer

Other than locating points and needling, was there anything else you found difficult?

Student 5

Not really. I don’t think there was anything else that felt particularly hard.

Interviewer

Then, would you say you felt confident about the CNT procedures?

Student 5

Yeah, our professor emphasized that part a lot during class, so I think I really got used to it. Even when we filmed the assignment procedures, we practiced things like proper handwashing steps, so I feel like I really internalized those procedures.

Interviewer

This might be a bit of a careful question, but since you’ve taken two semesters, I understand that each professor has a different teaching style, and the class requirements and the way they run the sessions also differ. Could you share what you think were the strengths and weaknesses of each, or which approach you found better?

Student 5

In Professor A’s class, there was a lot to learn — a lot of content to study — which was great, but at the same time, it felt a bit overwhelming because there was just so much. In Professor B’s class, the content was more compact, so it was clearer what to focus on. But compared to that, we didn’t learn CNT or ultrasound-related content from Professor B. Professor B also followed CNT well, but he didn’t emphasize it as much as Professor A did, so I think I learned those aspects more thoroughly from Professor A.

Interviewer

I see. Then, in terms of how the practice sessions were conducted, were there any differences between the two professors? Which approach did you find more helpful?

Student 5

We had much more time to actually practice during Professor B’s class. The demonstrations were kept short — within about 30 minutes — so we had plenty of time for hands-on practice, and that was really helpful.

Interviewer

So you think it’s better to have about 30 minutes of demostration and then move on to practice right away?

Student

Yes. That way, there’s no need to rush the point location or needle insertion. We could take our time to check things together with our group members, and the overall flow of practice felt more relaxed and smooth. But in Professor A’s class, we had to move a bit faster, so that part was a bit difficult.

Interviewer

I see. During the practice sessions, were there any unexpected incidents or accidents?

Student

No, I don’t think there were any particular incidents like that.

Interviewer

Did you observe any bleeding during the practice sessions?

Student

Yes, there was a fair amount of bleeding. But it wasn’t to the extent that hemostasis couldn’t be achieved — most of the time it was just a small amount of bleeding, so it never really caused any serious problems.

Interviewer

I see. During class, for example, when you first started practicing — if you didn’t know where the materials were, or what you were supposed to be doing at that moment — what did you usually do?

Student

I would ask other group members, or sometimes students from other groups. And I also asked the teaching assistants in the practice room when I wasn’t sure about something.

Interviewer

Then, what do you think could be improved to make the acupuncture training more effective?

Student

I think if there was an assignment to make sure that everyone had done their pre-class learning, it would have made the practice go smoother, all the group members would have come to class better prepared. Sometimes I couldn't watch the video and I was a little bit unprepared, so I wish there was something to do pre-class learning before the practice.

Interviewer

During class, when you didn’t understand something, was it easy to approach the professors or teaching assistants?

Student

Yes, they were always present in the lab. I don’t think there was ever a time when the professor or assistants weren’t there.

Interviewer

So, you mean asking questions went smoothly?

Student

Yes, that’s right.

Interviewer

There are several learning objectives for the acupuncture point practice course — are you familiar with them?

Student

No, I don’t think I really know them.

Interviewer

Ah, for example, one of the objectives is to “locate and perform needling on acupuncture points of the twelve meridians and the conception and governing vessels.” Another one is to “communicate effectively.” Do you think you had opportunities to practice communication during the sessions?

Student

Yes, I think I practiced quite a lot of communication between the physician and the patient. After needling, the physician would keep asking if the patient was okay, so that became a kind of communication practice. And when I was in the patient’s position, I’d tell the physician things like “this part feels uncomfortable” or “that spot hurt a bit,” so that also helped me practice communicating.

Interviewer

Then, let’s say you’re going on a medical volunteer work this summer, and you have to perform acupuncture on patients. How confident do you think you would feel? If 100% means complete confidence, what percentage would you give yourself?

Student

I think maybe around 60 or 70 percent. I think I could do it to that extent.

Interviewer

Then how do you think you could fill in the remaining 30 to 40 percent?

Student

I think I’d need to gain more hands-on experience with people around me — taking patient histories, thinking about which areas or meridians would be appropriate for needling, and practicing actual needling and follow-ups. I think repeating that kind of practical training would help.

Interviewer

Ah, maybe I didn’t phrase my question clearly — you wouldn’t be doing diagnosis yourself yet, since you haven’t learned that part. Suppose a senior student has already examined the patient and decided which acupuncture points should be needled. Then how confident would you feel performing the needling in that situation?

Student

In that case too, I think about 60 to 70 percent confident.

Interviewer

I see. So it’s about the same? Even if it’s not an actual treatment, you’d feel about that level of confidence when needling at designated acupuncture points?

Student

Yeah, that’s right. I feel pretty confident with the limbs, but I’m still a bit nervous about the trunk area. I think I need more practice there.

Interviewer

When you say you need more practice on trunk, what do you mean exactly?

Student

I think I need to overcome the fear a little more.

Interviewer

Ah, I see. Is that because the trunk has more high-risk areas?

Student

Yes, that’s right. So I’m still being very cautious about it.

Interviewer

And lastly, I’d like to ask about the *practice note*. How burdensome was it for you to write those notes?

Student

Well, since we had to write them every week, it was a bit of a burden. But since not just one person had to do all the writing, it wasn’t too bad. And because the content was mainly about what we did during class, it didn’t feel like a big burden overall.

Interviewer

While you were writing, did you ever feel like some of the content was repetitive?

Student

Yes, at the end of the practice note, there’s a section where each person writes what they realized or felt during the class. That part sometimes overlapped with the section above where we wrote about the *deqi* sensation. So I did feel there was some repetition between what we wrote above and what we wrote below. But since we had to write it for each acupuncture point, it wasn’t too repetitive overall—still, there were a few overlapping parts.

Interviewer

Did you understand what kind of content you needed to write in the practice note from the beginning?

Student

Yes, that was fine.

Interviewer

Did you ever refer to an example of how to write the practice note?

Student

Yes, I did refer to one.

Interviewer

Thank you so much for your time today. What you shared will be really helpful in improving the training class through this study. I really appreciate it.

**Interview with Student 6**

Interviewer

We’re going to start recording the interview now. Before we begin, I’d like to send you a small token of thanks for participating.

Student 6

Okay.

Interviewer

The interview will take about an hour. First, you took the acupuncture training course last semester with Professor A. Can you tell me about the atmosphere in your group? Was the practice conducted actively and enthusiastically?

Student 6

I think it was quite active. If someone didn’t know something, others would explain it. And since everyone had different areas of interest, some students who were interested in classical texts had already studied things like the **『**Huangdi Neijing**』** or related literature and would explain points based on that. Others who were interested in anatomy would explain acupuncture points in relation to anatomical structures. So we were constantly discussing and helping each other.

Interviewer

Sorry, but your voice sounds a bit echoey right now. It’s hard to hear clearly. Could you try saying something again, just anything, so we can test it?

Student 6

You can’t hear me well?

Interviewer

I can hear you, but it’s a bit echoey. YK, how about you?

Interviewer (YK)

I think it might be because I’m using the Bluetooth earbud microphone.

Interviewer

Ah, that’s why?

Interviewer (YK)

I think so.

Interviewer

Can you understand what he’s saying now? YK, can you hear him?

Student 6

Not really.

Interviewer

I can barely hear YK too, but since you’re closer now, it’s a bit easier.

Student 6

Is that so?

Interviewer

How about you, YK?

Interviewer (YK)

I think we need to hear a longer sentence to tell. Some parts get a little muffled in the middle.

Student 6

Should I try using the laptop’s built-in volume instead? I hope it’s not too loud.

Interviewer

Sure, can you give it a try?

Student 6

Okay. I can still hear my voice, so…

Interviewer

Yes, yes, that seems a bit better.

Student 6

Got it.

Interviewer

Great, thank you.

Student 6

Yes.

Interviewer

Okay, let’s start with the main questions. Are you familiar with the acupuncture training manual?

Student 6

I think we experienced it more practically than just as a text. It’s something we absorbed while actually doing the practice and writing the practice notes. Through the feedback we received during the class, we could check which parts of the manual were missing or unclear, and I think that helped us internalize it a bit.

Interviewer

When you say feedback, do you mean the feedback from the professor? What kind of feedback are you referring to?

Student 6

Yes, I’m talking about the feedback provided by the professor.

Interviewer

When and how do you receive the feedback from the professor?

Student 6

During the acupuncture training class, while we’re writing our practice notes, even if we’re needling the same acupuncture point, how we target the underlying structure can change the outcome. The professor points out the exact spots and also gives feedback on hygiene or other situations we might not have noticed. Sometimes this happens in class, and sometimes we get feedback through Google Classroom. We take that feedback into account for the next training class and update our practice notes accordingly. That’s basically how we approached it.

Interviewer

Have you ever seen the handwashing procedure?

Student 6

Yes, I have. We received manual posted in the acupuncture lab about handwashing, and during the very first class, the professor emphasized it as the most important part of CNT. Of course, needling the points is important, but more broadly, we learned how to approach needling safely, starting with handwashing.

Interviewer

I see. Have you ever seen the waste disposal manual?

Student 6

Yes, I have. We received some instructions verbally, and I’ve also seen it in the written manual. Those rules were enforced very strictly, so I remember them well.

Interviewer

And what about the equipment instruction manuals? Have you seen those?

Student 6

Yes, I have seen them too. But honestly, we use the equipment so routinely that it’s hard to recall the exact instructions verbally. I think I probably learned them as part of the habits we follow.

Interviewer

Then, at the beginning of the course, were there any moments when you weren’t sure how the training class were supposed to go, or what exactly you were supposed to be doing?

Student 6

Do you mean during the class?

Interviewer

Yes, that’s right.

Student 6

Are you referring to academic content, or…?

Interviewer

No, I mean, for example, during the first or second week—after the professor’s demonstration, when students started practicing on their own—were there times you didn’t know what to do, where to find materials, or how to proceed?

Student 6

Of course, at the beginning everything felt unfamiliar, so there were some awkward moments. I hadn’t fully memorized the manual yet, and since needling itself was a completely new experience, it didn’t come naturally at first. Even though I had read about it, it’s quite different to actually perform it. So at the beginning, I remember feeling a bit flustered. But as the time went on, whenever I was unsure, I’d look back at the manual, or ask the professor or the teaching assistants. We also helped each other out within the group, filling in what others didn’t know, so things went smoothly overall.

Interviewer

Alright. In each class, the professor would display the day’s schedule, learning objectives, and other information on the screen at the front, right? Did you see those materials?

Student 6

Yes, I did.

Interviewer

Then, during the training class, did you actually refer to those materials? Did you find them useful, or did you mostly just proceed without looking at them?

Student 6

As I recall, in the early part of the semester the professor mainly showed them through PowerPoint slides, but from around the middle of the semester, we were also given printed A4 handouts. Those sheets listed which acupuncture points we would be practicing that day, and what aspects of each point we should pay particular attention to. I remember referring to those handouts quite often. Even though we always had the **『**Handbook of Standard Acupuncture**』** or the textbook with us, it was difficult to grasp the overall scope of that day’s session at a glance. Sometimes while needling, we’d lose track of which meridian we were working on or forget key anatomical structures we needed to be mindful of. For example, when practicing on the Pericardium meridian, we had to constantly keep in mind the median nerve, or on the Spleen meridian, the tibial nerve. It was easy to overlook those general anatomical considerations.

So, having those printed materials or the PowerPoint at the beginning of class was very helpful. They allowed us to check which points we were supposed to needle, and what structures we should be focusing on for each point. It made it easier to review and confirm things on the spot. Overall, I think those materials were quite helpful.

Interviewer

Then, during the practice sessions, what kind of materials did you mainly refer to? You mentioned the handbook earlier — when you came across something you didn’t know, what sources did you usually consult?

Student 6

Mostly, I referred to the lecture materials provided by the professor. I wanted to make sure I was needling the exact location that the professor indicated as the target point, so I relied a lot on the notes I had taken directly on those lecture slides. I usually reviewed those notes on my tablet during practice. In addition to that, I also used the Acupuncture Points Handbook and other supplementary materials from time to time.

Interviewer

Among the notes you took, which parts were the most helpful during practice?

Student 6

It was especially helpful when the professor explained, for example, that a particular point lies “between muscles,” and then specified exactly which muscles it was between, or demonstrated the precise method for locating that point on the body. Those details were very useful.

Even though the textbook and lecture material slides describe the points in writing, it’s often difficult to grasp their exact anatomical locations just from text. The professor’s explanations during class — the conversational descriptions — helped clarify those parts. I wrote those explanations down and sometimes drew simple diagrams so that I could understand them more intuitively later. Those kinds of notes were particularly helpful during practice sessions.

Interviewer

Oh, so you also used illustrations when taking notes?

Student 6

Yes. There are some things that are hard to describe just in words, so I often drew simple diagrams — for example, sketches of the arm to mark the exact locations, or drawings showing the ulna and radius, the tibia and fibula, or even the pelvic bones, depending on which points I needed to locate.

I also referred to various materials — such as additional 3D anatomy resources that the professor introduced, or other anatomy textbooks — to find the most appropriate diagrams for each situation. I would then copy or adapt those illustrations into my notes so I could use them as references later.

Interviewer

Hmm, then approximately what percentage of your group members studied in advance before the class?

Student 6

In my opinion, well—this isn’t to sound like I’m exaggerating—but our group members were quite academic and diligent. Most of them studied in advance before each practice. Some even went further and did additional self-study on top of the assigned material. So I’d say almost everyone in our group had prepared beforehand. Honestly, there was also a bit of social pressure. If I showed up without reviewing, while others had already studied and were helping me, it felt like I wasn’t contributing equally. Since we all wanted to keep a good give-and-take dynamic, we kind of kept each other accountable in that sense. Overall, it created a positive atmosphere — we started off well and ended well.

Interviewer

Then, when you were preparing in advance, did you watch the videos uploaded on Google Classroom?

Student 6

I knew that we were supposed to refer to them, but I personally didn’t end up watching those videos.

Interviewer

Hmm, so what did you mainly use for your pre-study then?

Student 6

Mostly the lecture materials, I think.

Interviewer

The materials, I see. Are you at the train station right now? When do you have to leave?

Student 6

Oh, no — my train’s at 11, so I still have plenty of time.

Interviewer

I see. Then, if the pre-class videos on Google Classroom were to be newly produced, what kind of content do you think would be the most helpful?

Student 6

Hmm… if they were to be remade… Actually, from what I know, many of my classmates already refer quite a lot to the videos uploaded on the acupuncture point (meridian) theory Google Classroom. So even if the content doesn’t change drastically, I think it would still be very helpful. From my perspective, what would probably help the most is content that focuses on identifying acupuncture points based on the actual anatomical structures. I think that would be especially useful. Since theory and practice aren’t completely separate, it would also be nice if the videos could connect the two — like explaining why certain theories were developed or why certain indications and therapeutic effects are associated with particular points. But honestly, those parts are already well covered in the lecture sessions, so I think if we could just combine those two approaches effectively, it would be more than sufficient.

Interviewer

Then, since you’ve taken the practicum for two semesters, was there anything in particular that you found especially good about this semester’s practicum?

Student 6

When you say “good,” do you mean between Acupuncture Training 1 and 2?

Interviewer

Yes, since you’ve done the acupuncture training course over two semesters, and different professors conducted it, I assume the style and content were a bit different. What do you think were the strengths of the practicum style you experienced this semester?

Student 6

If I were to point out its strengths, I’d say that during the practicum, the professor always made it very clear what we should focus on, and what we need to prepare and practice to be able to perform properly in clinical settings. That clarity of purpose really helped us stay organized and consistent with our classmates, so we could all have a more standardized — and in a sense, optimal — practicum experience. I felt very strongly that everything was programmed in a very structured way, so I think that was a huge benefit. I think that (the manual) allowed us to do more standardized and optimal practice in a way.

Interviewer

Could you tell me a bit more specifically about what you meant when you said the program felt very structured?

Student 6

Sure. So, generally speaking, when we attend practical class—whether it’s Acupuncture Points or Herbal Medicine—it could just end with something simple, like in Herbal Medicine: “Try tasting this herb,” or in Acupuncture Points: “Here’s the point, now insert the needle here.” But in this training course, everything felt much more systematic. It wasn’t just about the needling itself — we learned the entire process step by step, as if we were actually in a clinical setting. For example, before needling, we first practiced handwashing and disinfection, then disinfecting the patient’s skin, and even how to help the patient feel more at ease during the procedure. We also learned about maintaining rapport with the patient and managing the whole flow — from preparation to needling to finishing the procedure. So the practicum really gave us a clear structure and direction for what we, as future physicians, should do from start to finish. That systematic approach was very helpful.

Interviewer

In the training class, students take turns performing different roles. When you were the physician, did you notice anything particular about your own performance — for example, things you thought you did well or areas you felt you needed to improve?

Student 6

Hmm… The biggest thing I realized was that following the CNT manual was actually much harder than I expected. If I had gone straight into a local clinic without experiencing this practicum first, I might have caused some kind of medical accident — and that thought really hit me.

I came to understand how easily we can be exposed to unhygienic conditions during needling, and how challenging it actually is to check and maintain all those steps while performing acupuncture. It made me realize that this kind of training is absolutely necessary.

So for me, at the beginning, that was probably the most difficult part of the acupuncture training course — keeping up with the hygiene standards and doing everything correctly. But as the time went on, I gradually became more familiar with it, and I think it was ultimately very helpful.

Interviewer

Do you feel there are any particular difficulties when it comes to locating acupuncture points or performing needling?

Student 6

As I mentioned earlier, in the beginning, it was challenging to complete the entire process — following the CNT hygiene manual, performing the needling, interacting with the patient, and referring to the lecture materials — all within the allotted time.

Other than that, I think if we hadn’t done any pre-study beforehand, it would have been even more difficult.

Interviewer

You said it was hard to finish within the given time. What do you think was the main reason for that?

Student 6

The main reason is that in the early stages of the training class, our basic knowledge of the acupuncture manual or the points themselves was somewhat insufficient. Because of this lack of foundational understanding, it was naturally more difficult to complete the tasks efficiently.

Interviewer

As you progressed, did you become able to complete the tasks within the allotted time?

Student 6

Yes. At the very beginning, we often ran out of time, and sometimes stayed after the acupuncture practicum ended to finish the parts we hadn’t completed. But as time went on and we became more accustomed to the procedures, it became easier to complete everything efficiently.

Also, as the training course continued, it became clearer which aspects the professor emphasized and which parts of acupuncture training were considered most important. This made pre-study more efficient.

Additionally, the professor gave group assignment as a sort of midterm evaluation. Our group’s approach was to simulate a real clinic: perform diagnosis, explain to the patient why we chose specific points, and justify our selection. Practicing in this way helped us become fully comfortable by the end of the semester, allowing us to save time without compromising the quality of our practice.

Interviewer

Regarding the group assignment, did you encounter any difficulties while working on it?

Student 6

Yes. Our project involved selecting a specific condition, choosing five acupuncture points to treat it, and performing needling on those points. Although we approached it earnestly with the knowledge we had up to our second-year coursework, we still lacked some of the additional knowledge needed—like from diagnostics or acupuncture medicine courses that come later.

So, trying to identify a condition and determine its treatment with the limited knowledge from second year made the project somewhat challenging. However, we were able to get support from fourth-year students or graduates who had experience, which made the process somewhat easier and more manageable.

Interviewer

Do you think that group projects like this will be helpful for you in your future clinical practice after graduation?

Student 6

I think any experience like this is helpful. Especially since Wonkwang University College of Korean Medicine aims to train students with strong clinical skills, the process of practicing these skills is essential. Activities like CPX or OSCE align well with that goal, so in that sense, these projects are definitely valuable. Moreover, even with the knowledge of acupuncture points I already have, being able to explain it to someone else—even a simulated patient—helps me practice persuading and communicating what I know. This process clarifies the direction for future study and highlights what I should focus on, so I believe it will be very helpful going forward.

Interviewer

I see. During the group assignment, were there moments when you weren’t sure how to proceed or had questions about the assignment format?

Student 6

There might have been a few questions at the beginning, but as we went along, I asked the professor and also teaching assistant, and almost all of them were resolved.

Interviewer

I see. And during the training class, have you also taken on the role of the patient?

Student 6

Yes, I have.

Interviewer

While acting as the patient, did you have any experiences that made you embarrassed?

Student 6

Yes, at the very first time, I was nervous. My muscles were a bit tense, with my sympathetic nervous system more activated than usual, so when the needle was inserted, some muscles reacted and slightly went into spasm. That situation was a little surprising. From this experience, I realized the importance of reassuring the patient or patient. Even if the procedure is routine for the physician, it can be unfamiliar or stressful for the person receiving it. So I learned that communication is crucial, and helping the patient relax—shifting from sympathetic to parasympathetic activation—is important.

Interviewer

When your group members practiced acupuncture on you as the patient, did you notice any points where they did well or areas where they were lacking?

Student 6

Other group members… as I mentioned before, in the early stages of our class, everyone was inexperienced. We were pressed for time, so sometimes the physician would insert the needle quickly without properly reassuring the patient, which could startle them. Although we tried to follow hygiene protocols as best as we could, there were naturally some shortcomings early on. Over time, through feedback—both written in the practice notes and verbal—we gradually corrected these issues. So as the semester progressed, the quality of practice improved noticeably.

Interviewer

Was the atmosphere for giving and receiving feedback among your group comfortable?

Student 6

Yes, it was very comfortable. Our group had a good relationship, so there was no burden at all in sharing feedback.

Interviewer

How did you form your group? Did you choose people you were already close with?

Student 6

Yes. Usually, when attending classes, we sat near each other and helped each other understand difficult points, forming a sort of study group. So when we created the acupuncture practice group this year, we continued that dynamic from last year. Because of this, there were no conflicts or complaints during practice or feedback sessions. Everyone approached things logically and respectfully.

Interviewer

So, overall, you formed your group entirely voluntarily?

Student 6

Yes, exactly.

Interviewer

I see. So regarding the exam at the end of the semester, which parts did you feel confident about?

Student 6

The parts I felt confident about were mainly those I had focused on most during acupuncture practice. Specifically, identifying the exact locations of each acupuncture point. Rather than just measuring distances—like saying “3 cun from this joint”—I relied on anatomical landmarks as reference points. Being able to explain these landmarks to the professor while locating the points was the aspect I felt confident in.

Interviewer

When you actually took the exam, how much were you able to complete within the 5-minute limit?

Student 6

I felt quite confident explaining the points, so I spent a lot of time on explanations. Because of that, I wasn’t able to complete the final needling and withdrawal within the time limit. That was a bit disappointing, but overall, I felt fairly satisfied with my performance.

Interviewer

Were there any parts you felt were lacking during the exam?

Student 6

If I had to point out shortcomings, I think I could have managed my time better. Also, when marking the acupuncture points with the Betadine cotton, my fingers slightly missed the exact spot, so although my explanations were clear, the marking wasn’t perfectly accurate. Some of that may have been due to nervousness, but I think these are issues that could also happen during actual needling, so it’s something I’d like to improve.

Interviewer

Now that the course is over, suppose this summer you go on a medical volunteering and need to perform acupuncture on a patient.

Student 6

Since we hadn't learned clinical courses yet, a fourth-year senior selected the treatment points for me.

Interviewer

Yes, that’s right.

Student 6

I think I could perform the needling with about 90% confidence. Assuming I don’t forget the locations of the points, I’d feel around 90% confident.

Interviewer

So, thinking back to last semester’s acupuncture training course, is there anything you think could be improved?

Student 6

If I had to suggest an improvement… I think it would be helpful if the practice sessions were a bit more flexible with time. In the beginning it can be time consuming with the CNTs, but we get more comfortable with the CNTs and the phases of practice towards the end of the course, so I would like them to be a little bit flexible and elastic with the time, for example reducing the number of acupuncture points at the beginning of the course and increasing it a little bit towards the end of the course.

Interviewer

During the practice sessions, do you think the time your group had for practicing was sufficient? Out of the total three hours, do you feel there was enough time for group practice? After the professor’s demostration, the group practice begins—how would you describe the absolute amount of time you had for that?

Student 6

If we’re talking about absolute time, towards the later sessions, there was actually plenty of time, maybe even a bit extra. But in the early sessions, the schedule felt very tight. Because of that, our group sometimes felt pressured to finish within the allotted time, which made us rush through certain parts without even realizing it.

Interviewer

Understood. I’d like to ask just one more thing. I understand that you were shown ultrasound images during class—while looking at the ultrasound, were you able to understand which area was being scanned and what the structures were?

Student 6

Are you referring to what’s displayed on the scanning screen?

Interviewer

Yes, yes. I’m curious whether you were able to understand, from the ultrasound image, which area was being scanned and what the structure was.

Student 6

At first, since we had basically no prior knowledge of ultrasound, even though the professor briefly explained things—like what to look for, how different levels of penetration show up on the scan, and so on—it was still difficult for us. Being new to ultrasound, we could only interpret it at a very basic level: “If the probe is placed here, there are probably muscles underneath, and maybe nerves and blood vessels.” Beyond that, we didn’t have the ability to immediately understand the structures just by looking at the scan, so initially it was a bit challenging.

However, the professor also demonstrated directly using the ultrasound device and explained, “This is a muscle, this is a nerve, this is a blood vessel,” which helped us understand what we were seeing.

Through this, I realized just how little I knew about ultrasound, and I also thought that I need to become more familiar with ultrasound techniques so that I can use them diagnostically in the future—especially now, given that recent court rulings emphasize its importance. This made me recognize the need to acquire hands-on skills with ultrasound.

Interviewer

Alright, got it. Have you watched any of the ultrasound lectures on Google Classroom?

Student 6

As part of my personal self-study, I realized, as I mentioned earlier, that I was quite lacking in knowledge about ultrasound. Feeling my own ignorance, I went through a self-study process using videos created by the professor that briefly explained the principles of ultrasound and how to interpret it for the first time. I based my learning on those videos, and I think that, even if only minimally, they were helpful.

Interviewer

I see, thank you. I think that covers all my questions for today. Thank you so much for participating in the interview—it will be very helpful in further improving the acupuncture training course. I really appreciate you taking the time today.

Student 6

Yes, thank you.

**Interview with Student 7**

Interviewer

Today, I’d like to ask you a few questions about the *Acupuncture Training* class.

Student 7

Sure, of course.

Interviewer

I might go a bit differently from the written questionnaire. Could you tell me briefly what the atmosphere was like in your *group* during the *practice note* sessions?

Student 7

In our group, when we were doing the practice, like for example with meridians such as the Liver meridian or the Bladder meridian—which have a lot of acupuncture points—we divided things up a bit. So we kind of split the work, and I think overall the practice went pretty smoothly within the time we had.

Interviewer

So you mean the role assignments worked out well, right?

Student 7

Yeah, yeah, definitely.

Interviewer

So, did you have any conflicts or uncomfortable moments within your group?

Student 7

Well, when we were writing our *practice notes*, we divided the work among group members. But honestly, there were a few times when it was hard to collect everyone’s parts and put them together on time. There were a few moments when it felt like things might get tense, but we managed to talk it out and resolve it pretty well as a group.

Interviewer

I see. Then, do you know what the *Acupuncture Learning Manual* is?

Student 7

From what I remember, the professor really emphasized the importance of CNT and how we communicate with the *subject*. That lesson actually came in really handy. I recently went to a medical volunteer activity through our alumni association, and I realized how important that communication aspect was. A friend of mine who also took acupuncture training class last semester said the same thing — that following the manual really helped a lot in practice.

Interviewer

You mentioned that what you learned had a big impact during the volunteer work with the alumni association. Could you share a specific example of that?

Student 7

Sure. For instance, when I volunteered before—well, I can’t say exactly which club it was—but back then, people weren’t really that sensitive about how to sort medical waste or anything like that. But this time, during the medical volunteer service, even though a lot of *patient* came in all at once for treatment, everyone still tried hard to maintain hygiene. They made sure to separate waste properly, kept the beds and treatment areas clean, and paid attention to sanitation overall. Watching that, I realized that in a real clinical situation, those habits really help prevent potential problems like infection. And actually, the *patients’* satisfaction level was really high—many of them said the place felt clean and pleasant.

Interviewer

Let me share my screen for a moment.

Student 7

Sure.

Interviewer

Okay, just a second. I’d like to show you the 2023 Google Classroom.

Student 7

Alright.

Interviewer

Can you see my screen now?

Student 7

Yes, I can see it.

Interviewer

So here, you can see the lesson plans—

Student 7

Yeah.

Interviewer

They include the topic, objectives, and the overall flow of the class.

Student 7

Right.

Interviewer

And then, here—

Student 7

Yes.

Interviewer

Yeah, on this part of the screen—

Student 7

Okay.

Interviewer

Here, it shows things like how to use the equipment, how to wash hands properly, and the waste disposal manual. Have you ever actually looked through these materials yourself?

Student 7

Yeah, I think I did when we were working on the professor’s group assignment. For example, with handwashing—we know there are six steps, but honestly, we didn’t always follow them strictly before practice sessions. But because of that assignment, it kind of gave us a reason to review those things again—like the proper handwashing steps, how to handle materials, and how to dispose of waste correctly.

Interviewer

I see. Do you remember around when you looked at those materials?

Student 7

I think it was around mid-April, maybe right before or after the midterm exams.

Interviewer

Got it. Were there any parts of the materials that you found hard to understand?

Student 7

Not really. I thought they were pretty well-organized and clear, so nothing felt confusing or difficult.

Interviewer

Then have you also seen this equipment manual before?

Student 7

Yes, I think we looked at it once during the orientation at the beginning. We didn’t really go over it in Professor A’s class, but I remember we did when we had Professor B before.

Interviewer

So this same material was available back then too?

Student 7

Yes, I’m pretty sure it was the same one. I remember Professor B explaining it using the same PowerPoint slides that Professor A had originally made.

Interviewer

I see. And about the lesson plan you just looked at—was that kind of material provided for every class session?

Student 7

Yes, it was. Every week, the professor handed out printed lesson plans for us to follow.

Interviewer

I see. So when you were doing the practice, did you actually refer to these materials while you worked?

Student 7

Um… I can’t say we looked at them every single time, but we did when we hit a bit of a wall. You know, there are so many *acupuncture points*, including the standard extra points and other extra points, so sometimes we weren’t sure which ones to focus on first. In those moments, the materials kind of served as a guideline—like, “Okay, this is the one we should prioritize checking.”

Interviewer

Were there ever times when you started a session in the *lab* without being totally sure about what range or area you were supposed to cover that day?

Student 7

Yeah, that did happen once or twice. Since the class moved at a pretty fast pace, there were a few times when we weren’t completely clear on what we needed to cover before starting.

Interviewer

So when you weren’t quite sure what you were supposed to do, do you mean that you referred to the lesson plan for guidance?

Student 7

Yes, exactly. We used it kind of as a guideline—especially when there were a lot of *acupuncture points* to go through, like in the Bladder or Gallbladder meridians. In those cases, since we couldn’t cover everything, we focused on the essential ones, like the extra points or the standard extra points. The lesson plan didn’t list every single point, though. So when deciding which ones to prioritize—mainly because of time constraints—we often referred to that material for guidance.

Interviewer

But that document didn’t show each *acupuncture point* in detail, right?

Student 7

I think it only listed the names.

Interviewer

What about the meridian names?

Student 7

I’m pretty sure those were included too—the names of each *acupuncture point* and the meridian it belonged to.

Interviewer

I see. So, do you usually cover the extra points during practice? Did you practice them as well?

Student 7

At first, we actually tried to go through all the extra points. But for meridians with a lot of *acupuncture points*, like the Bladder meridian, we decided to focus only on the standard extra points. Covering all the extra points just wasn’t feasible time-wise. Since we weren’t that experienced with the practice yet, and we weren’t very comfortable with needling, it was really hard to get everything done within the time available.

Interviewer

You mentioned that it was hard to finish within the time. Why do you think there wasn’t enough time?

Student 7

Well, first, our practice sessions were in the evening, so maybe our focus wasn’t at its best. Also, some of us weren’t very skilled at needling yet, which slowed things down. Another big factor was locating the points. For example, in textbooks, you’re supposed to be able to feel certain bony landmarks when palpating, but in reality, depending on the patient’s subcutaneous fat or other differences, it’s not always easy to feel them. Spending a lot of time just trying to locate the points often left us with little time to actually do the needling. It took way more time than we expected.

Interviewer

When you practiced with your group, how much time did you actually have for the hands-on work, excluding the professor’s demonstration?

Student 7

Including the professor’s explanation, I think the class was about three hours long. So we had roughly two hours for practice, after subtracting about an hour. But sometimes, if the professor explained a bit longer, it could be closer to an hour and a half.

Interviewer

I see. Do you think that hour and a half was enough time for the group practice?

Student 7

I felt it was a bit short. For topics with fewer points, we could finish quickly, but when there were a lot of points, we needed a little more time. Honestly, we were always kind of rushed. It also depends on the group. Some groups finished faster, but in our group, we wanted to be precise with point location and such, so it actually took us longer than expected.

Interviewer

So for the other groups that finished faster, why do you think they were able to finish quickly?

Student 7

I think they just located the points and needled right away—point, needle, point, needle. In our group, we spent a bit more time on point location, discussing, like, “Is this the right spot?” We’d ask each other, “Does this feel right to you?” or “I’m not sure, what do you think?” That took extra time. For the faster groups, I guess they just palpated the point and immediately did the needling. Actually, there was one time in class when he got a bit upset because we were taking extra time on points like GB20 and other points around the neck, and other groups probably just needled right away. So yeah, I think that’s why they finished faster.

Interviewer

Now I’d like to ask about the main learning objectives of the *acupuncture training* class. There are five in total. I’ll show you the lesson plan again—can you see it on your screen?

Student 7

Yes, I can.

Interviewer

One of the objectives is, *“Be able to measure the patient’s vital signs.”* Do you feel like you’ve achieved this goal yourself?

Student 7

I think so, to some extent. Since we practiced every week, I feel like I got the hang of it. But for things like blood pressure, since we were using a stethoscope, we weren’t always sure if we were measuring it accurately. For the other vital signs, I don’t think there was much problem measuring them.

Interviewer

How confident are you in this skill?

Student 7

Hmm… for blood pressure measurement, if full confidence is 10, I’d say maybe a 6. I practice it every time, but I can’t say I’m completely accurate. I sometimes doubt whether I’m doing it exactly right.

Interviewer

Next, the second objective is, *“Be able to accurately locate the acupuncture points on the meridian.”* If we consider this for the 14 meridians, how would you rate your achievement on a scale of 1 to 10?

Student 7

I think… well, I’m not completely sure if the points I locate are 100% accurate, but I’d say around 8 or 9.

Interviewer

Then the third objective is, *“Perform acupuncture treatment in a hygienic and safe manner.”* This relates a bit to CNT. How do you feel about this one?

Student 7

I’d say this is almost a 10. I really learned a lot here.

Interviewer

What do you think is the reason you became so good at this?

Student 7

Honestly, the professor emphasized this as really important, and we practiced it every session. I think it became a habit. Even recently, during the medical volunteer work, I noticed that many people didn’t properly separate medical waste or sharps from general trash. Seeing that just kept reminding me of what we practiced. I probably wouldn’t have thought much about it before, but now it sticks with me. So I guess practicing it every session made it a personal habit.

Interviewer

I see, because you practiced it every session. Got it.
Next, the fourth objective is, *“Be able to appropriately use the necessary medical devices during the procedure.”* This includes acupuncture needling, electroacupuncture, scalp acupuncture, and other techniques you learned, as well as the various tools you used in the *lab*. How do you feel about this one?

Student 7

I’d say around 8 or 9. The professor showed us so many different devices, which was really impressive. For example, for ear acupuncture, there was an acupuncture detector that gives a small electrical signal when you touch the points. We also learned about cupping, dry cupping, and Gua Sha therapy with specific tools. We got to see a lot of devices and learned how to use them properly. I especially enjoyed learning about scalp acupuncture—it was really interesting. The professor explained not just how to perform the techniques, but also the criteria and principles behind them, which made it fun and engaging for me.

Interviewer

So, thinking about what you just said, how confident do you feel about doing it yourself?"

Student 7

But since we’ve seen them once already, I’d say my confidence is probably above a 6.

Interviewer

Did you actually get to try those devices yourself?

Student 7

Yes. Whenever there was a new or interesting device, we’d always ask the professor to let us try it. He even said our group seemed really enthusiastic and hardworking. We’re the type that just has to try everything before leaving, especially the interesting ones.

Interviewer

Then, the fifth objective — *“Can accurately record the details of the procedure.”* This refers to whether you were able to accurately write down what you did in your practice note. How do you feel about that?

Student 7

I think there weren’t really any problems with recording accurately, especially after doing this last semester and again this semester. All of our group members took turns acting as the observer and practiced writing the practice notes themselves. Since I was the group leader, I would always review the notes afterward — for example, I’d look over the week’s records once they were submitted. And I noticed that, definitely, compared to last semester, we all improved a lot. Even within this semester, everyone — including myself — got noticeably better toward the end.

Interviewer

Could you give an example of what made you feel that your recording skills had improved?

Student 7

For instance, we’ve started noting specific things like, *“This part of the practice could have been risky,”* or *“This was a situation where we should have followed CNT principles more carefully.”* Before, our writing was kind of vague and general, but now we identify what was lacking and suggest what could have been done better. Seeing that kind of progress made me think, *“Oh, we’ve really improved.”*

Interviewer

So, was it difficult for you to record the details of the procedures you performed?

Student 7

It actually was difficult at first. In the beginning, I wasn’t sure what kind of content should be included. But later on, I found myself reflecting more on my own actions — things like, *“This kind of procedure isn’t appropriate,”* or *“This could be burdensome for the patient.”* Over time, my writing shifted in that direction. At first, the way I wrote was quite vague, even when I looked back at it myself.

Interviewer

Have you ever seen an example of a practice note before?

Student 7

Yes, I have.

Interviewer

Then, did that example help you when writing your own practice notes?

Student 7

It helped a lot. I think it was during our very first orientation session for acupuncture practice that we looked at an example. I remember thinking, *“Wow, it’s possible to write this much detail and recall every part of the process so thoroughly.”* So at the beginning, I tried to model my own notes after that example as much as possible. We even shared it in our group chat for the practice members, saying, *“They wrote it like this, so let’s try to write at least this thoroughly, too.”*

Interviewer

Then, among the contents of the practice note, was there anything you felt was repetitive or unnecessary?

Student 7

Actually, for each acupuncture point, the pre-learning content was quite helpful. But in the sections on CNT or hygienic acupuncture techniques, I did wonder if it was really necessary to repeat the theoretical content there.

Interviewer

Anything else?

Student 7

Other than that, I think everything else was in its proper place and necessary. So I don’t think there were any other issues.

Interviewer

Then, about the items written by the observer — honestly, it’d be great if they were all checked properly. Do you think those checks were actually carried out each time? Or was it more of a routine “checked” without much review?

Student 7

To be honest, depending on the time and situation, we often did it rather routinely. But one of my group member was always very thorough with those things, always checking carefully. So at first, I was also doing it pretty routinely, but later I tried to be more attentive after seeing that person. I actually learned from them. But yes, to be honest, I think we did it routinely quite a lot of the time.

Interviewer

Then, did you understand what kind of content you were supposed to write in the observation and feedback section?

Student 7

Yes. When we wrote it, we usually focused on things like whether hemostasis was done properly, if disinfection was missed, or if communication and posture guidance for the patient were appropriate. We’d write things like, “This could have been uncomfortable for the patient,” or “This wasn’t a desirable practice.”

Interviewer

I see. Then do you think that overlaps a bit with the part about “the patient’s sensation” and “what you learned or felt from the experience as a patient”?

Student 7

Yes, that part does overlap. For example, if the patient said it hurt a lot, then in the “self-reflection” section, we’d write something like, “Acupuncture around this point can be painful, so I should be more careful when performing it in the future.” We often wrote it that way.

Interviewer

Originally, the plan was for the physician to fill out the *physician* section, the patient to fill out the *patient* section, and the observer to fill out the *observer* section. For this *procedure details* part, it was supposed to be written by the physician. In your group, did the physician actually write this section during practice?

Student 7

In our group, while performing the acupuncture, the observer would briefly ask questions and take notes — like, “Which needle did you use?” “How did you locate the point?” “What was the insertion angle?” It wasn’t possible to record everything during the session, so afterward, each person who performed acupuncture on a specific point wrote the section for that point themselves. So yes, each physician wrote their own parts after the session.

Interviewer

Ah, so during the practice, the observer would briefly write this section, and then afterward the physician would fill in the rest — is that right?

Student 7

Yes, that way it fit better with the time available, and we were able to cover more acupuncture points within the session.

Interviewer

I see. But since the observer has to write this part, were you able to properly observe whether the physician was performing the procedure correctly while writing?

Student 7

For me, it was actually visible while watching. I think it depends on the person, but for me and a few other classmates, we could see what was going on even while writing. So I didn’t really find it difficult. For example, I could notice when the insertion angle should have been perpendicular but was tilted, or when an oblique insertion was done incorrectly, or if there was bleeding but hemostasis wasn’t performed properly, or when someone grabbed the cotton with bare hands — those kinds of things were just noticeable.

Interviewer

Right. So, the original intent of that part actually relates to one of the course objectives — “to accurately record the procedure performed.” You know how Korean medicine doctors have to write their own medical charts, right?

Student 7

Yes, yes.

Interviewer

Since record-keeping is something the physician must do directly, we wanted you to think of this as a kind of medical record — to document what you did during needling. Doctors usually use laptops now, and in actual clinics, they record notes on the computer in real time rather than by hand. So the idea was for the physician who performed the needling to write it themselves after finishing, while others continued with other acupuncture points. The record-keeping and needling would rotate — one person needling, one person charting, and so on. Do you think that method would be difficult to do during practice?

Student 7

So, does that mean that multiple students would practice on the same acupuncture point?

Interviewer

Not exactly — each acupuncture point would be needled by one physician. For example, if one person needled *LI4*, they would ask the patient afterward, “How did it feel when you received the needle?” Then, based on that response, the physician would write the note themselves — not the observer or the patient. After finishing *LI4*, that physician would move to the computer to record it, while another student performed needling at the next point, say *LI5*. It would rotate like that. How do you think that method would work?

Student 7

We actually tried something like that — a rotation system where one person needled while another wrote — and sometimes it worked, but sometimes it didn’t. When we were writing, we always asked the patient about their impression after needling — like how they felt or whether there was any sensation. But the problem was that when we tried to do that rotation in real time, it got hard to focus on the next point. For example, while someone was still writing or talking to the patient, the next person couldn’t fully concentrate on locating the next point or feeling for *deqi* properly, because their attention was divided. So it was a bit difficult to do consistently. It worked at times, but not always — that was our experience.

Interviewer

So, you mentioned the patient’s impressions earlier — how do you think it would be if you didn’t have to write that part down?

Student 7

Then I think the rotation system would actually work. Because the physician could just talk briefly about their own experience, and the next person could immediately start locating and needling the next point. In that case, rotation would be more feasible.

Interviewer

I see. Now, about the patient’s impressions — for example, if we consider unusually strong pain, excitement, or other notable responses — out of roughly 180 acupuncture points practiced in a semester, how many do you think showed meaningful reactions like that?

Student 7

I can’t remember all of them exactly, but I think there were maybe two or three such cases during a session — sometimes three or four at most, and sometimes none at all.

Interviewer

What kind of reactions do you remember most clearly?

Student 7

Mostly pain responses — patients feeling sharp pain or sudden discomfort, or reflexive movements like a leg or arm suddenly jerking upward. I think it happened because the needle stimulated a nerve. Those kinds of responses were the ones I remember.

Interviewer

When the patient reported strong pain, how did you usually respond?

Student 7

We usually stopped needling right away. Then we applied pressure to the area — pressing with cotton for hemostasis — and gently massaged around the point while continuously asking if the patient was okay. After a short break, we would start locating the point again.

Interviewer

And after a short rest, did the patient usually feel better?

Student 7

Yes, yes. There were no serious or critical side effects.

Interviewer

What about when you thought you might have hit a nerve and there was a reflex reaction?

Student 7

In those cases, we would just check right away and ask if the person was okay. I actually experienced that myself once — when I was needling a point, the patient’s leg suddenly jerked backward, which surprised everyone. But it didn’t really hurt much, so we just checked in, made sure everything was fine, and moved on. There wasn’t any real problem.

Interviewer

Then, in the case of bleeding, was it usually resolved just by applying pressure?

Student 7

Yes. We applied pressure carefully, and bleeding was never really a problem for us.

Interviewer

Aside from mild reactions, have you ever heard what to do in a more serious situation — for example, if the patient faints or dizziness during treatment?

Student 7

Yes, we were told about that briefly during class.

Interviewer

What kind of instructions did you receive?

Student 7

We were told to have the patient lie down comfortably, and if the condition seemed serious, we should send them to the hospital.

Interviewer

I see. While you were filling out the practice note, were there any parts you thought were unnecessary?

Student 7

Actually, I felt that all of it was necessary. While writing down what I had done during needling, I sometimes realized things — like, “Oh, I should’ve inserted the needle vertically, but I actually did it obliquely.” So I don’t think there were any unnecessary parts.

Interviewer

Did you usually fill out the section on the point location method — the pre-learning part?

Student 7

I think I mostly just wrote down the same content, like what was on the PowerPoint or from the study notes we had to submit.

Interviewer

Oh, did you usually refer to that part during practice, or did you just write it down?

Student 7

When I hadn’t studied enough beforehand, it was helpful to refer to it. But when I had already studied, I didn’t really need to look at it.

Interviewer

Then, you know the pre-learning videos—did you watch those?

Student 7

We always planned to watch them, but usually ended up not having time. Because of things like the practice notes and other coursework, we kept saying we’d watch them later but mostly didn’t. I did watch one once, though—for example, the bloodletting therapy video. I watched it while writing my practice note, and I remember it being quite well-structured.

Interviewer

I see. I heard that from midterms onward, you started doing the OSCE. Did you participate in that? As a test-taker, an evaluator, or in some other role?

Student 7

I participated in it. I took the test, and I also helped with it.

Interviewer

Right. So, as a test-taker, you had to perform the given task within five minutes—how was that? Did you feel nervous?

Student 7

I did—quite a lot, actually. And when I watched my group members while scoring them, I noticed the same thing. Since everyone only had five minutes and they were being watched by their group members, they tended to stutter a bit or their hands would tremble. So, it definitely felt like a pressured situation, but everyone tried to do their best under that pressure.

Interviewer

Then, if you compare before midterms, when you didn’t have the OSCE, and after midterms, when you did—did you notice any difference in your attitude or concentration during practice?

Student 7

Definitely. During the OSCE, both the examiner and the practitioner were really focused.

Interviewer

You mean during the regular practice sessions?

Student 7

Yes, yes. During the usual practice sessions, there wasn’t much difference in attitude, but during the OSCE itself, everyone was clearly much more focused.

Interviewer

So, there wasn’t much difference in your regular practice sessions before that. Then, were you able to receive feedback properly as well?

Student 7

You mean feedback from the professor during class?

Interviewer

No, no — I mean after the OSCE, did the observer give you feedback?

Student 7

Yes. We went through each item one by one, reading them aloud, saying which parts were done well and which weren’t. And for the parts that weren’t done well, they also explained why they weren’t done properly.

Interviewer

Then, when you participated as a *patient*, did you have any particular impressions or realizations?

Student 7

When I participated as a patient… ah, yes, there was something like that. For example, I realized that points on the face are quite painful, so I should be more careful when I perform needling myself — I should think about the pain the patient might feel. I think that was the main thing. Actually getting needled and feeling, “Oh, this area really hurts,” made me realize a lot.

Interviewer

When you participated as a patient, did the physicians make sure you were in a comfortable position during the procedure?

Student 7

Yes. Even if someone started in an uncomfortable position at first, someone in the group would always point it out — like, “This looks uncomfortable,” or “Let’s give them a pillow.” I think I was lucky to be in a good group; someone always made sure to address things like that.

Interviewer

I’d also like to ask about the ultrasound part. When the professor demonstrated using the ultrasound, you could see the image on the screen, right? Out of 100%, how much of what was shown do you think you understood?

Student 7

Honestly speaking, I have to confess — when I was focused, I understood really well, but when I was tired or exhausted, I often couldn’t follow along. Since the class was in the evening, there were times I just couldn’t take it all in. If I had to put it in numbers, I’d say maybe around half of the time I was really paying attention. And when I did, I actually understood it pretty well. But I noticed that some of my classmates couldn’t understand it as much. I think that difference came from how much anatomical knowledge we already had. If you had some background knowledge, you could kind of follow what the professor was saying — like, “Ah, that structure must be over there,” even if you didn’t understand everything precisely. But for those who didn’t have that anatomy foundation, I think it just felt like listening without really grasping what was going on.

Interviewer

Then, when you look at the professor’s hand holding the probe and the ultrasound image on the screen, are you able to tell what anatomical structure you’re seeing?

Student 7

When I’m in class, yes, I can usually tell. When the professor places the probe and then moves it to another spot, I can get a general sense of what structure is being shown. It might not be completely accurate, but I can roughly understand what I’m looking at.

Interviewer

Which do you think helps your understanding more — when the professor demonstrates in front of the whole class, or when they come to each group and demonstrate directly in front of you?

Student 7

I think when the professor demonstrates in front of our group, the focus and engagement are definitely higher. Usually, the professor explains everything to the entire class first, and then walks around with a tablet, letting each group take turns holding the probe. So, since we hear the explanation once and then actually do it ourselves, I think that repetition helps a lot with understanding.

Interviewer

So you feel that being able to hear the explanation once and then do it again helps you understand better?

Student 7

Yes, I think so. The professor usually explains once, and then personally comes around to let each group handle the probe. So at first, I only have a vague understanding from the explanation, but when it’s demonstrated again right in front of me, it becomes much clearer.

Interviewer

Then, when you took the final exam, which part do you think you did well? Did you feel confident about anything?

Student 7

I think I felt confident about locating the acupuncture points. I practiced a lot, so I knew where things were. If I remember correctly, out of the five questions on the exam, three were *extra points*. Usually, when one or two extra points appear, many students panic. I was nervous too — at one point, I forgot to locate a point, and the professor asked, “Aren’t you going to locate this one?” That’s when I realized I hadn’t done it and did it right away. But even then, I didn’t really panic about having three extra points. I think I had a little bit of confidence in finding points because I practiced a lot. I realized that if I practice, I can do this, and at first I thought I couldn’t do it at all, but then I did it.

Interviewer

Then, when you entered the exam room, was there any part you felt less confident about?

Student 7

I think I wasn’t very confident about the anatomical structures — their exact locations and accuracy.

Interviewer

How about the hygienic aspects, like handwashing or disinfection?

Student 7

I was actually a bit surprised by myself. For example, during practice, many people just disinfect once per acupuncture point and then toss the swab aside without much thought. But during the exam, I checked each step carefully, and that surprised me. As far as I remember, I didn’t lose any points for hygiene. I realized that your attitude during regular practice sessions really matters. People who, even if it’s tedious, consistently organized materials properly, disinfected carefully, and applied pressure well didn’t lose points in that area. But those who were used to rushing — skipping disinfection or not pressing firmly — had those habits show up during the exam, and it affected their performance.

Interviewer

So, you mean you disinfected and disposed of each item properly for every point, right?

Student 7

Yes, that’s right.

Interviewer

You’ve completed a full semester of the acupuncture training course. Is there anything you think could be improved?

Student 7

I heard that the acupuncture training course will be reorganized into four semesters later on, which I think will make things better.

Interviewer

Into four semesters?

Student 7

Yes, I heard it’ll be divided into four semesters, combined with practice sessions. I also heard that *herbal medicine and prescription* will be organized that way too — into four semesters. The total time per class will be reduced, so I think that’s a good change. In acupuncture theory II and III, the workload was quite heavy, so honestly, it was a bit hard to keep up — especially with the practice hours. During acupuncture theory I, I was able to come prepared, already knowing the material before entering the lab, which made it possible to review and check my understanding during practice. But in acupuncture theory II and III, there was just too much content. I also had to study for other subjects and write study diaries, so it felt like a big load. Sometimes I’d enter the lab without properly studying beforehand and feel like, “What am I even doing right now?” Especially when I was tired — if I hadn’t studied enough, I couldn’t participate as actively. There were definitely week-to-week differences in my engagement. Still, every time that happened, there was always at least one member in my group who had studied the theory thoroughly, so the practice itself could continue smoothly.

Interviewer

Then how do you think this part could be improved?

Student 7

Well, since I heard the acupuncture theory course will be divided into multiple semesters, I think that change alone will improve things quite a bit — especially with the new curriculum being introduced.

Interviewer

Aside from the curriculum change, if the course continues as it is now, how do you think it could be improved?

Student 7

In that case, I think it would help to balance the number of acupuncture points covered in each session. Right now, even though the sessions are divided by body region, some regions still include a lot more points than others. So if the distribution of acupoints across sessions were adjusted to be more even, I think the practice sessions would be much more manageable.

Interviewer

When you say it was divided by body region, do you mean that different meridians were mixed together by region?

Student 7

No, in our case, for example, when we studied the Bladder Meridian, we would do the back part one day and the lower part another day — so it was divided within the same meridian. But I think Professor B divided them by region instead. So maybe if the number of acupuncture points could be distributed more evenly — either by dividing within a single meridian by region, or sometimes by combining points from several meridians — that would make things better.

For example, as I remember, when we practiced the Lung Meridian, there weren’t many acupoints since it was one of the first sessions, so we finished pretty quickly. But later on, as the number of acupoints increased, there were more cases where we ran out of time. Especially in acupuncture theory II and III, the Bladder Meridian and Gallbladder Meridian had such a heavy load that it was really hard to finish within the class time.

Interviewer

You mentioned that you also practiced by body region. When the meridians were mixed by region, did that make things confusing compared to practicing by meridian?

Student 7

At first, it was confusing. But later, when we actually took the exam with Professor B, it turned out to be more convenient. Since we had already practiced recognizing which side or area belonged to which meridian during class, that prior experience helped during the exam.

On the other hand, with Professor A, since we practiced by region, when preparing for the exam, I first had to think about which meridian each point belonged to. I had to figure out the line or location first, and then connect it to what I had learned.

So I think it really depends on preference — whether you want to struggle a bit at first or have to integrate everything later on.

Interviewer

I think we’ve covered everything I wanted to ask. Is there anything else you’d like to add?

Student 7

No, nothing in particular.

Interviewer

Alright, thank you for participating today.

Student 7

Thank you, good work.

Interviewer

Okay, I’ll end the interview here.
